# Phase-changing citrate macromolecule combats oxidative pancreatic islet damage, enables islet engraftment and function in the omentum

**DOI:** 10.1126/sciadv.adk3081

**Published:** 2024-06-07

**Authors:** Jacqueline A. Burke, Yunxiao Zhu, Xiaomin Zhang, Peter D. Rios, Ira Joshi, Daisy Lopez, Hafsa Nasir, Sharon Roberts, Quetzalli Rodriguez, James McGarrigle, David Cook, Jose Oberholzer, Xunrong Luo, Guillermo A. Ameer

**Affiliations:** ^1^ Department of Biomedical Engineering, Northwestern University, Evanston, IL 60208, USA.; ^2^ Center for Advanced Regenerative Engineering, Northwestern University, Evanston, IL 60208, USA.; ^3^ Department of Surgery, Feinberg School of Medicine, Northwestern University, Chicago, IL 60611, USA.; ^4^ CellTrans Inc., Chicago, IL 60612, USA.; ^5^ Duke Transplant Center, Duke University School of Medicine, Durham, NC 27710, USA.; ^6^ Chemistry of Life Processes Institute, Northwestern University, Evanston, IL 60208 USA.; ^7^ Simpson Querrey Institute, Northwestern University, Chicago, IL 60611, USA.; ^8^ International Institute for Nanotechnology, Northwestern University, Evanston, IL 60208, USA.

## Abstract

Clinical outcomes for total-pancreatectomy followed by intraportal islet autotransplantation (TP-IAT) to treat chronic pancreatitis (CP) are suboptimal due to pancreas inflammation, oxidative stress during islet isolation, and harsh engraftment conditions in the liver’s vasculature. We describe a thermoresponsive, antioxidant macromolecule poly(polyethylene glycol citrate-*co*-*N*-isopropylacrylamide) (PPCN) to protect islet redox status and function and to enable extrahepatic omentum islet engraftment. PPCN solution transitions from a liquid to a hydrogel at body temperature. Islets entrapped in PPCN and exposed to oxidative stress remain functional and support long-term euglycemia, in contrast to islets entrapped in a plasma-thrombin biologic scaffold. In the nonhuman primate (NHP) omentum, PPCN is well-tolerated and mostly resorbed without fibrosis at 3 months after implantation. In NHPs, autologous omentum islet transplantation using PPCN restores normoglycemia with minimal exogenous insulin requirements for >100 days. This preclinical study supports TP-IAT with PPCN in patients with CP and highlights antioxidant properties as a mechanism for islet function preservation.

## Teaser

A thermoresponsive, antioxidative polymer enables autologous omentum islet transplantation in nonhuman primates.

## INTRODUCTION

Chronic pancreatitis (CP) is a disease characterized by the progressive inflammation of the pancreatic acinar tissue (*1*). The etiology of this inflammation is varied and can include smoking, alcohol use, gallstones, and genetic variants (*1*). Patients with CP suffer from chronic abdominal pain, often leading to hospitalization and disability. As the inflammation persists, pancreatic tissue is destroyed as apoptotic acinar cells increase 10-fold and the insulin-producing beta cell mass is reduced by 29% (*2*, *3*). There is no all-encompassing treatment for this disease. Management includes lifestyle modifications, optimization of pain medications, and nutrition. Surgical intervention is the last resort for relieving pain when pharmacological options no longer work (*1*, *4*).

Patients with CP that are unable to alleviate symptoms via less invasive measures often undergo total pancreatectomy (TP) followed by intrahepatic islet autologous transplantation (IAT), a procedure referred to as TP-IAT (*5*). Although islet transplantation has been improved through standardized islet isolation procedures, long-term outcomes remain suboptimal. Currently, islets are transplanted to the liver via intraportal infusion. Deleterious conditions such as liver thrombosis, the instant blood-mediated inflammatory reaction (IBMIR), and oxidative stress are reported to contribute to substantial damage to the transplanted islets (*6*–*8*). Approximately, 50 to 80% of the islets are destroyed via isolation and infusion (*9*–*11*). Given that patients with CP have reduced islet masses due to inflammatory conditions, this additional islet loss renders one-third of patients undergoing TP-IAT diabetic after the surgery (*12*). These findings highlight the importance of the transplant site and the need to support islet survival throughout the transplantation process. Therefore, there is a need for an alternate, extrahepatic islet transplant site and new islet delivery methods that provide a supportive microenvironment for islet function to improve the outcome of TP-IAT for patients with CP.

The successful engraftment of islets at an extrahepatic site requires a microenvironment that can provide adequate vascularization and protection against oxidative stress conditions (*13*, *14*). Extrahepatic locations for islet transplantation have traditionally been limited to organ capsules, which have failed to be clinically adopted due to the invasive nature of the procedure (*15*). The omentum has recently been investigated as a transplantation site in animals and humans due to its easy access, high vascularity, and potential to localize islets using preformed, solid scaffolds (*16*). However, the use of solid scaffolds makes it difficult to implement minimally invasive techniques, requires additional considerations for the placement and distribution of the islets within the scaffold, and can exacerbate inflammatory responses leading to fibrosis, limiting the widespread application of the procedure (*17*). To address this issue, a recent clinical trial is investigating the feasibility of using autologous plasma and recombinant thrombin in a two-step endoscope-enabled procedure to deliver and secure allogeneic islets to the omentum for the treatment of type 1 diabetes (T1D) (*18*–*20*). The trial is still ongoing with two out of the three patients meeting goals for glycemic control without hypoglycemic episodes. However, the authors of this trial cite a need for improved “oxygen delivery and neovascularization” at the omentum site and enhanced strategies to “minimize immunosuppression” given the allogeneic nature of the transplantation (*19*). While the omentum has several advantages as a site for islet transplantation, including being highly vascularized and confined, the omentum has a physiological immunoregulatory function (*21*). Lymphoid aggregates known as “milky spots” reside within the omentum (*22*). These “spots” are primarily composed of mononuclear phagocytes, macrophages, B cells, and T cells. The omentum has been shown to activate and migrate to contain inflammation. The volume of the omentum can expand, producing a large number of inflammation cells, to contain injury or infection. These inflammatory cells have stem cell markers that enhance their regenerative potential. More investigation is required to determine how the immunoregulatory function of the omentum may affect islet transplantation. Different effects may be observed for autologous and allogeneic transplantation given that immune cell populations are site-specific and that the cell types involved in autoimmune and allogeneic immune responses may differ. Given that alloreactive cell populations do not pose a threat to autologous transplantation, we set out to investigate whether the omentum could potentially be a viable transplantation site for patients with CP if a scaffold can provide the proper microenvironment to protect and support the islets.

**Fig. 1.**
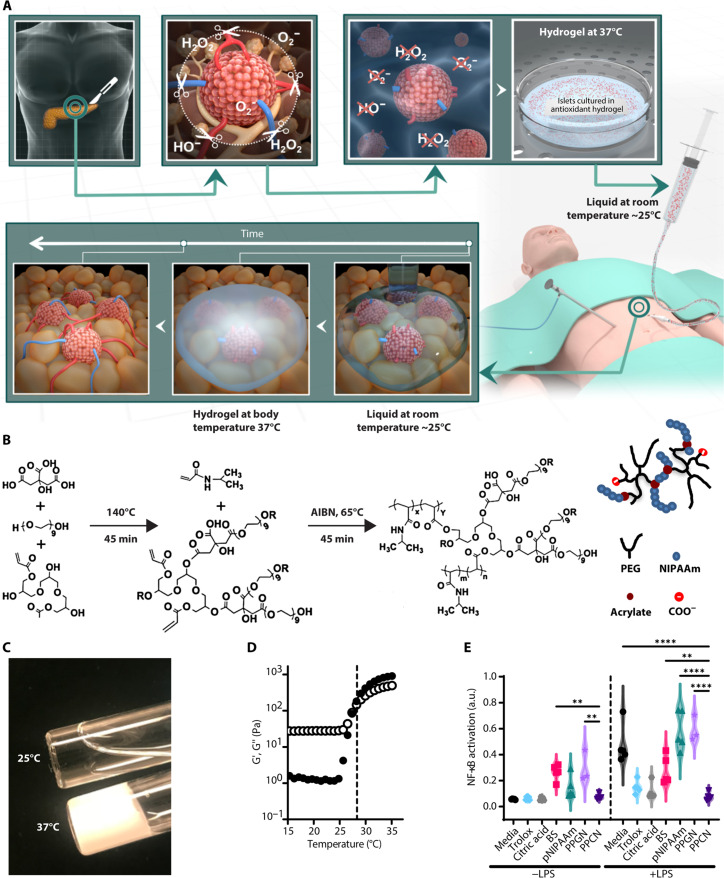
PPCN’s thermoresponsive and antioxidative properties are suitable for islet preservation. (**A**) Schematic of PPCN-mediated islet protection against oxidative stress to preserve islet function throughout omentum transplantation. Top: Organ removal, islet tissue isolation, and transfer to a room temperature (25°C) islet culture media containing the antioxidant thermoresponsive macromolecule PPCN that protects islets against oxidative damage during culture. Bottom: The thermoresponsive, phase-changing property of PPCN allows easy delivery of the islets in the liquid, localization through body temperature-induced (37°C) gelation, and engraftment of islets into the omentum using laparoscopic surgery. (**B**) Schematic illustrating the synthesis of PPCN. (**C**) Digital photo showing the thermoresponsive transition of PPCN from a liquid (25°C) to a hydrogel (37°C). All samples were prepared in phosphate-buffered saline (PBS) at a concentration of 100 mg/ml and neutralized to pH 7.4. (**D**) Rheological determination of the lower critical solution temperature of the PPCN (black marker, storage modulus G′; white marker, loss modulus G″). (**E**) Assessment of antioxidative properties for protection of RAW-Blue macrophage cells against lipopolysaccharide (LPS)-induced nuclear factor κB (NF-κB) activation via Quanti-Blue cell–based assay. All data are presented as mean NF-κB activation (a.u., arbitrary units) ± SD with **P < 0.01; ****P < 0.0001 relative to PPCN. Treatments without LPS are compared to PPCN without LPS. Treatments with LPS are compared to PPCN with LPS. Statistical significance was determined by two-way analysis of variance (ANOVA) with Tukey’s multiple comparisons test (*n* = 5).

We hypothesized that body temperature-induced phase change of an antioxidant, water-soluble, degradable macromolecule would (i) facilitate easy entrapment of islets and transportation to the omentum, (ii) enable islet localization and engraftment on the target tissue upon delivery, and (iii) significantly counter the negative effects of oxidative stress on islets after enduring pancreatitis, enzymatic isolation, and transplantation (Fig. 1A). Poly(polyethylene glycol citrate-*co*-*N*-isopropylacrylamide) (PPCN) is a citrate-containing macromolecule with a lower critical solution temperature (LCST) that allows the transition from a liquid to a gel at physiological body temperature and has intrinsic antioxidant properties that mitigate oxidative damage to tissues (*23*–*27*). In this study, we report that PPCN protects mouse and human islets from oxidative stress–induced damage during the in vitro culture process as well as in vivo upon transplantation to the mouse fat pad. We also show that the application of PPCN to the omentum of nonhuman primates (NHPs) is safe and does not induce a deleterious foreign body response as the hydrogel is bioresorbable. Last, we demonstrate that PPCN can support IAT in an NHP model for over 100 days, resulting in vascularized islets and minimal exogenous insulin requirements. As oxidative stress plays a pivotal role in the pathogenesis of pancreatitis, agents that can ameliorate oxidative stress should improve islet transplantation outcomes.

## RESULTS

### PPCN is anti-inflammatory, maintains islet viability, and supports insulin secretion in culture (Fig. 1A)

PPCN was prepared via a two-step synthesis starting with a polycondensation reaction comprising citric acid, polyethylene glycol (PEG), and glycerol 1,3-diglycerolate diacrylate followed by free-radical polymerization with *N*-isopropylacrylamide (NIPAAm) (Fig. 1B). PPCN dissolved in phosphate-buffered saline (PBS) exhibits a reversible liquid to solid phase transition at the LCST of 28°C, which is lower than that of the homopolymer poly(*N*-isopropylacrylamide) (pNIPAAm) (32°C) (Fig. 1, B to D). Successful synthesis was confirmed using proton nuclear magnetic resonance (^H^NMR), Fourier transform infrared spectroscopy (FTIR) (fig. S1), and rheology (Fig. 1D). The aforementioned LCST enables PPCN to be easily applied as a liquid to target tissues in the body along with the islets at room temperature (~25°C) and to facilitate islet entrapment at these locations via gelation at 37°C (Fig. 1, A, C, and D) (*28*).

The anti-inflammatory properties of PPCN were confirmed in vitro as per the inhibition of nuclear factor κB (NF-κB) activation in the RAW-Blue cell line (Fig. 1E). RAW-Blue cells are engineered RAW264.7 macrophages that are used to evaluate the intracellular antioxidant response due to lipopolysaccharide (LPS)-induced NF-κB expression. Antioxidants are known to suppress NF-κB activation and the subsequent transcription of inflammation-related genes (*18*, *29*). Cells exposed to LPS in the presence of the antioxidant Trolox, an analog of vitamin E, reduced NF-κB expression by 68% relative to cells exposed to LPS in cell culture media. PPCN exhibited an 84% reduction in NF-κB expression (Fig. 1E). Cells exposed to a hydrogel formed from a biologic scaffold (BS), a clinically used plasma-thrombin hydrogel for islet transplantation to the omentum in humans (*18*), effected a 41% inhibition of NF-κB expression (Fig. 1E). A non-antioxidant version of PPCN made with glutaric acid, instead of citric acid (fig. S2), referred to as poly(polyethylene glycol glutarate-*co*-*N*-isopropylacrylamide) (PPGN), did not elicit the protective effects of PPCN on cells. Of note, we have previously shown that PPCN confers antioxidative properties due to the combination of citric acid and diols (*25*, *30*–*32*). These intrinsic antioxidant properties include free-radical scavenging, iron chelation, and lipid peroxidation inhibition. The replacement of citric acid with glutaric acid eliminates these antioxidative properties.

**Fig. 2.**
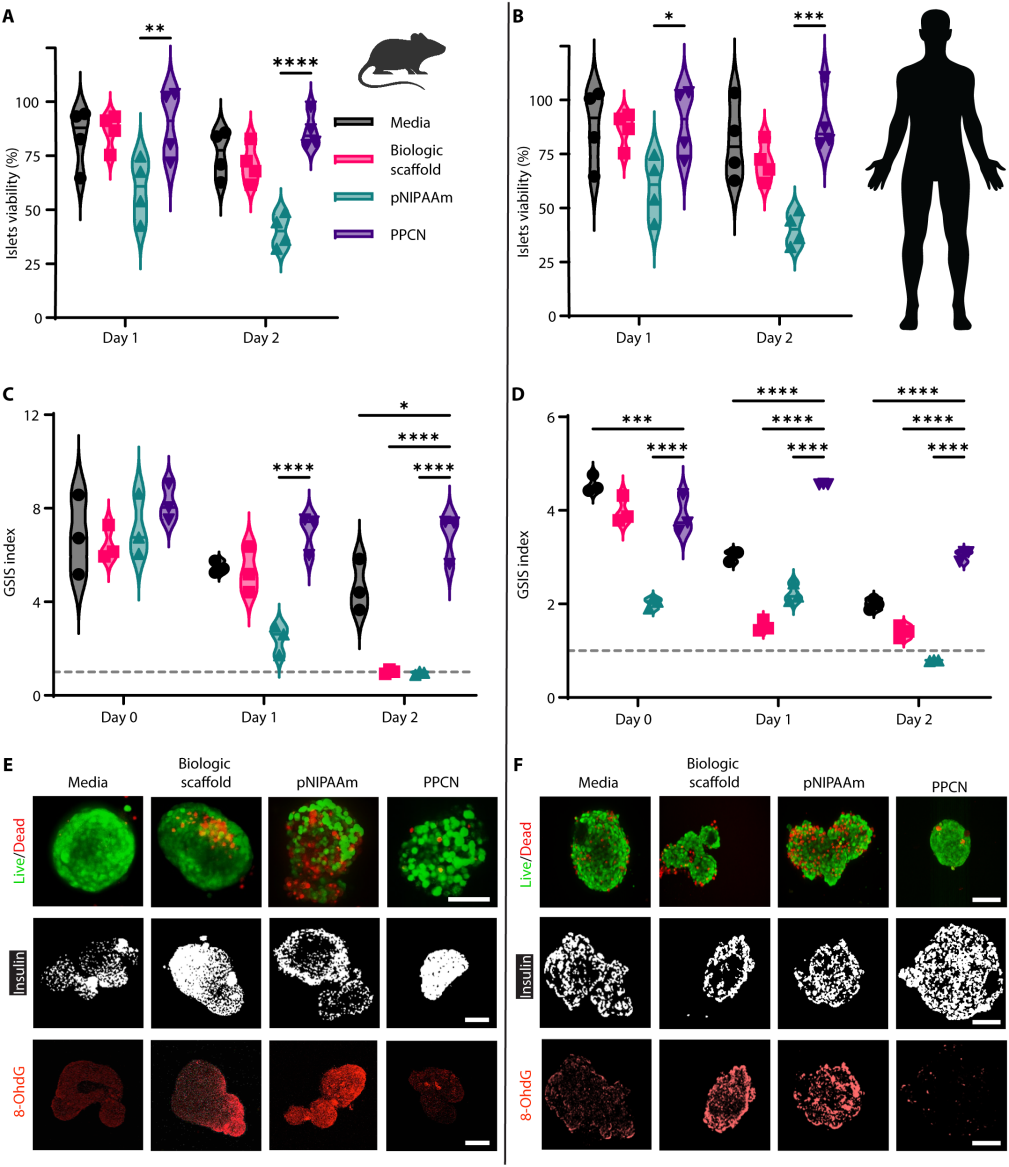
PPCN facilitates the preservation of both mouse and human islet viability and insulin secretion function in vitro. Human islet characteristics after isolation can be found in table S1. (**A** and **B**) Mouse and human islet viability as measured fluorescently by resazurin reduction after prolonged culture under various conditions. (**C** and **D**) Corresponding glucose-stimulated insulin secretion (GSIS) index of the cultured islets. The dashed gray line indicates a GSIS index of 1. All data are presented as means ± SD with *P < 0.05; **P < 0.01; ***P < 0.001; ****P < 0.0001 relative to PPCN. Statistical significance was determined by two-way ANOVA with Tukey’s multiple comparisons test (*n* = 3 to 5). Secreted insulin concentrations from islet exposure to low (2.8 mM) and high (28 mM) glucose concentrations can be found in figs. S4 and S5. (**E** and **F**) Live/Dead and immunostaining of mouse islets for insulin and 8-oxo-2′-deoxyguanosine (8-OHdG) after 2 days of ex vivo culture under various conditions. Scale bars, 100 μm.

To assess the impact of PPCN on islet function, the viability and insulin secretion ability of mouse (Fig. 2, A, C, and E) and human (Fig. 2, B, D, and F) islets were evaluated in vitro both in standard suspension culture and after entrapment in BS, PPCN, or pNIPAAm. Freshly isolated islets were either cultured on tissue culture plastic or mixed with autologous BS, PPCN, or pNIPAAm solutions at room temperature. Islets were successfully entrapped in each hydrogel by adding thrombin to the BS or incubating the islet-PPCN or islet-pNIPAAm mixtures at 37°C. The viability of the entrapped islets was monitored over time with the resazurin assay (Fig. 2). For both mouse (Fig. 2A) and human (Fig. 2B) islets, viability was maintained at 48 hours of in vitro culture except for the pNIPAAm-treated group. Islets from both species entrapped in pNIPAAm experienced a viability loss of 40% at 24 hours and 60% at 48 hours (Fig. 2, A and B).

Islet functionality was measured by insulin secretion in response to glucose stimulation using the in vitro glucose-stimulated insulin secretion (GSIS) test, which reports a stimulation index (the GSIS index) (Fig. 2, C and D, and figs. S4 and S5). A GSIS index of 1 or below indicates the complete loss of glucose response (indicated by the dashed gray line in Fig. 2, C and D) (*33*). During in vitro culture, at day 0, mouse islets in all four groups have an average GSIS index of 7.15 ± 1.23 (Fig. 2C and fig. S4). For mouse islets, the GSIS index of the pNIPAAm group drops by 65% at 24 hours of culture, which is consistent with the loss of islet viability observed in this group (Fig. 2C and fig. S4). A relatively smaller drop in the GSIS index was measured for the other three groups (26% for suspension culture, 17.5% for BS, and 14.8% for PPCN) (Fig. 2C and fig. S4). Islets cultured in BS for 48 hours also lost their glucose responsiveness as their GSIS index dropped from 6.45 ± 0.72 to 1.01 ± 0.09 (Fig. 2C and fig. S4). Partial disassembly of the BS-entrapped islets was also observed after 2 days in culture. Within 24 to 48 hours of culture, a 25 and 7.3% decrease in GSIS index was observed for islets in suspension culture and islets in PPCN, respectively, confirming the protective role of PPCN (Fig. 2C and fig. S4). Similar results were observed for human islets (Fig. 2, B and D, and fig. S5).

Live/Dead staining of the mouse and human islets after 24 hours of culture confirmed the presence of many dead cells in the pNIPAAm-treated group, whereas islets in suspension culture, BS, or PPCN exhibited similarly high viabilities (Fig. 2, E and F). Intracellular insulin staining also revealed that murine islets cultured in pNIPAAm and human islets cultured in BS showed signs of an insulin-deficient core, whereas islets cultured in PPCN showed uniform insulin expression across the entire islet structure. To assess whether the protective effects of PPCN were due to its antioxidant properties, we probed for the nuclear DNA oxidation marker 8-oxo-2′-deoxyguanosine (8-OHdG) on both mouse and human islets after 48 hours in culture. Staining for 8-OHdG revealed significantly more oxidized residues in islets with lower function as measured by the GSIS index (Fig. 2, C to F). In conclusion, culturing islets in PPCN preserve sensitivity to glucose and associated insulin secretion function, likely by minimizing oxidation.

### PPCN protects islets against induced oxidative stress, thereby preserving function during culture

To further understand whether the antioxidant property of PPCN would preserve islet viability and function, redox-sensitive islets were created by expressing the transgene for the redox-sensitive green fluorescent protein (roGFP) gene in the cytosol of freshly harvested islets via a lentiviral vector. The incorporation of one disulfide bond between cysteine-A147 and -A204 in the protein structure of roGFP enables its use as a redox reporter that can indicate the oxidation status through the quantification of the relative fluorescence intensity at two excitation wavelengths 405 and 488 nm (fig. S3, A and B) (*34*). Under normal reduced conditions, the protein is excited at 488 nm; however, upon exposure to an oxidizing environment, the disulfide bond formation leads to a shift in the excitation wavelength that peaks at 405 nm. This shift provides a signal difference that can be used to monitor and quantify the islet’s redox status within scaffolds by measuring the ratio between the fluorescence intensity emission after excitation at 405 or 488 nm. Transgene expression of roGFP in the cytosol did not affect the viability or insulin secretion function of both human and mouse islets (fig. S3, C to F).

**Fig. 3.**
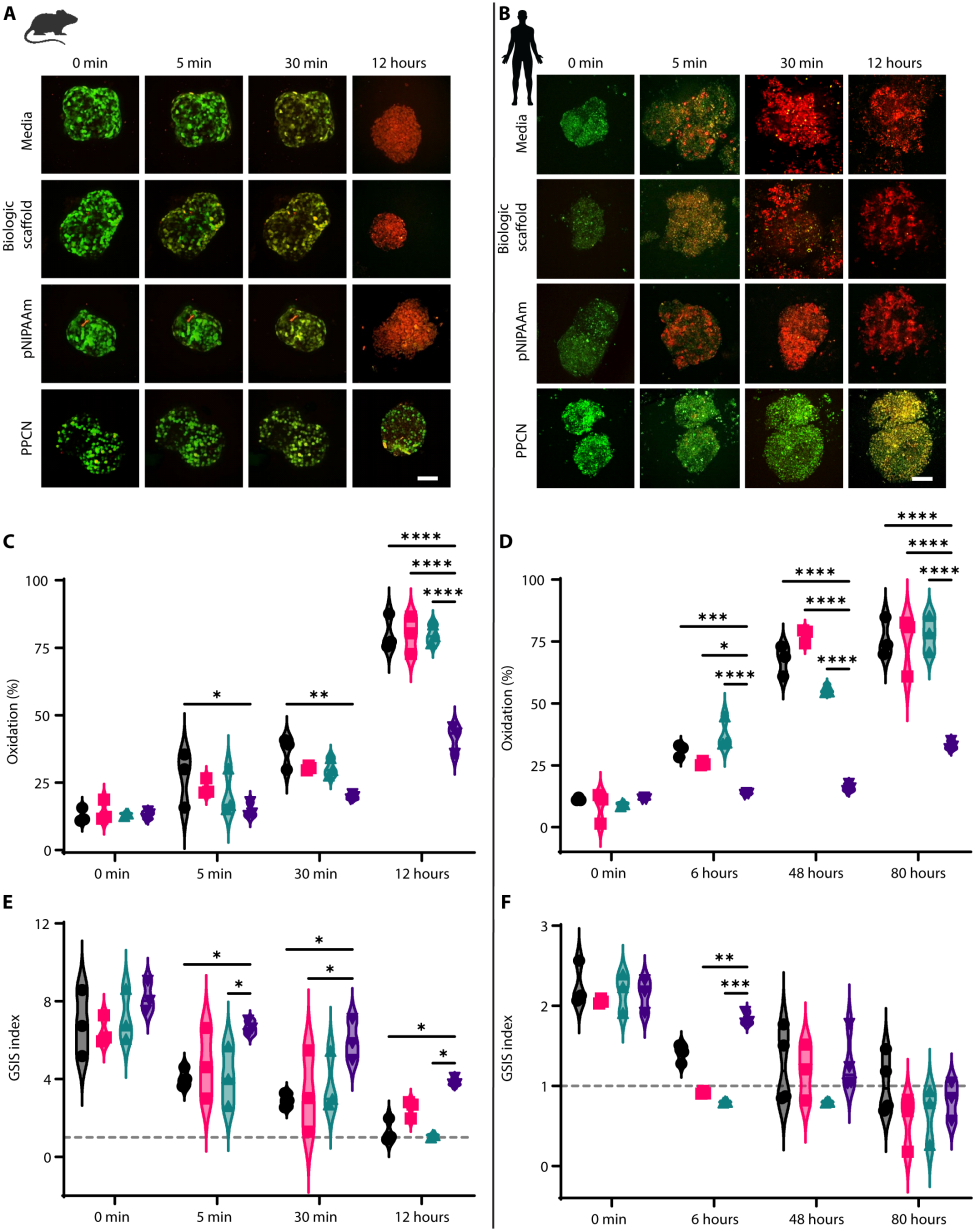
PPCN protects mouse and human islets against physiologic oxidative stress levels induced in vitro. Human islet characteristics after isolation can be found in table S1. (**A** and **B**) Oxidation rate of mouse and human islets, respectively, overexpressing roGFP under various culture conditions. H_2_O_2_ (10 μM) was used to induce oxidative damage (green = 488 nm excitation signal, reduced status; red = 405 nm excitation signal, oxidized status). Scale bars, 100 μm. (**C** and **D**) Quantification of oxidation (%) in roGFP-overexpressing islets. (**E** and **F**) GSIS index of islets stressed with H_2_O_2_. The dashed gray line indicates a GSIS index of 1. All data are presented as means ± SD with *P < 0.05; **P < 0.01; ***P < 0.001; ****P < 0.0001 relative to PPCN. Statistical significance was determined by two-way ANOVA with Tukey’s multiple comparisons test (*n* = 3 to 5). Secreted insulin concentrations from islet exposure to low (2.8 mM) and high (28 mM) glucose concentrations following H_2_O_2_ can be found in figs. S6 and S7.

To mimic oxidative damage to islets associated with CP in vitro, 10 μM hydrogen peroxide (H_2_O_2_) was added to each of the islet culture environments described in the aforementioned paragraph. This H_2_O_2_ concentration was chosen to mimic physiological H_2_O_2_ concentrations produced by acinar cells in experimental models of pancreatitis (*35*). Confocal microscopy imaging was used to monitor the progression of oxidative damage in both mouse and human islets (Fig. 3, A and B). At time 0, before the introduction of the H_2_O_2_, a dominant signal at 488 nm (reduced form shown as green) was observed in islets from all four groups with a baseline average oxidation percentage of 13% for mouse islets and 10% for human islets (Fig. 3, A to D). Five minutes after introducing H_2_O_2_, an increase in oxidation of 27.0, 23.1, 20.6, and 14.6% was measured for mouse islets in the control media suspension culture, BS, pNIPAAm, and PPCN, respectively. At 30 min, the oxidation significantly increased for islets cultured in media suspension (36.5%), BS (30.4%), and pNIPAAm (30.4%), whereas oxidation of islets entrapped in PPCN increased to 19.7%. At 12 hours, 44% oxidation was observed in the PPCN group, whereas approximately 80% oxidation was observed in the remaining groups. A similar trend was also observed for human islets (Fig. 3, B and D); however, human islets appeared more resistant to oxidation overall.

To study the correlation between the progression of oxidative damage and loss of islet function, the roGFP-measured oxidation was compared to the GSIS index (Fig. 3, A to F). Six hours after introducing H_2_O_2_, an increase in oxidation to 31.1, 25.8, 37.6, and 13.5% was measured for human islets in control media suspension culture, BS, pNIPAAm, and PPCN, respectively (Fig. 3, B and D). PPCN’s protective effect is more evident at the 80-hour time point, as 76.3% of the human islets cultured in the other three environments showed signs of oxidation compared to 34.0% of those cultured in PPCN (Fig. 3D). Significant islet disaggregation was observed in the control media suspension culture, BS, and pNIPAAm groups, whereas the morphology of the islets entrapped in PPCN remained intact. GSIS index of both mouse and human islets were measured at similar time points relative to oxidation studies (Fig. 3, E and F). For the mouse islets, a decrease in the GSIS index was observed as early as 5 min after introducing the H_2_O_2_. Relative to *t* = 0, at 5 min, the GSIS index for islets suspended in cell culture media, BS, pNIPAAm, and PPCN decreased by 40.2, 26.5, 42.7and 18.9% to 4.08, 4.74, 4.09, and 6.64, respectively. At 30 min, the GSIS index for islets in media, BS, pNIPAAm, and PPCN decreased to 2.90, 2.93, 3.70, and 5.97, respectively. At 12 hours, islets in media, BS, and pNIPAAm lost glucose responsiveness, as indicated by GSIS index of approximately 1 (indicated by a dashed gray line). A GSIS index equal to or less than 1 specifies that the islets are not properly sensing glucose concentration and responding with the appropriate insulin secretion (*33*). In contrast, at 12 hours, islets in PPCN were able to maintain a GSIS stimulation index of 3.84, confirming the protective properties of PPCN (Fig. 3E and fig. S6). To assess whether the results obtained with murine cells would also translate to human cells, human islets were evaluated using the same experimental setup (Fig. 3F and fig. S7). The results demonstrated a significant loss of function for the human islets cultured in media suspension at the 6-hour time point. Loss of function comes markedly early for the human islets, as compared to murine islets, which showed functional loss after 12 hours when in media suspension culture. At the 6-hour time point, the GSIS index dropped from 2.15 to 1.40, 2.06 to 0.92, 2.18 to 0.80, and 2.13 to1.83 for islets in media suspension culture, BS, pNIPAAm, and PPCN, respectively. Of note, human islets in media suspension culture, BS, and pNIPAAm completely lost insulin secretion response to glucose. In contrast, PPCN reduced islet function loss as per the GSIS index, demonstrating superiority to the other test materials (Fig. 3F).

### PPCN is a versatile islet delivery vehicle that preserves islet function in vivo

**Fig. 4.**
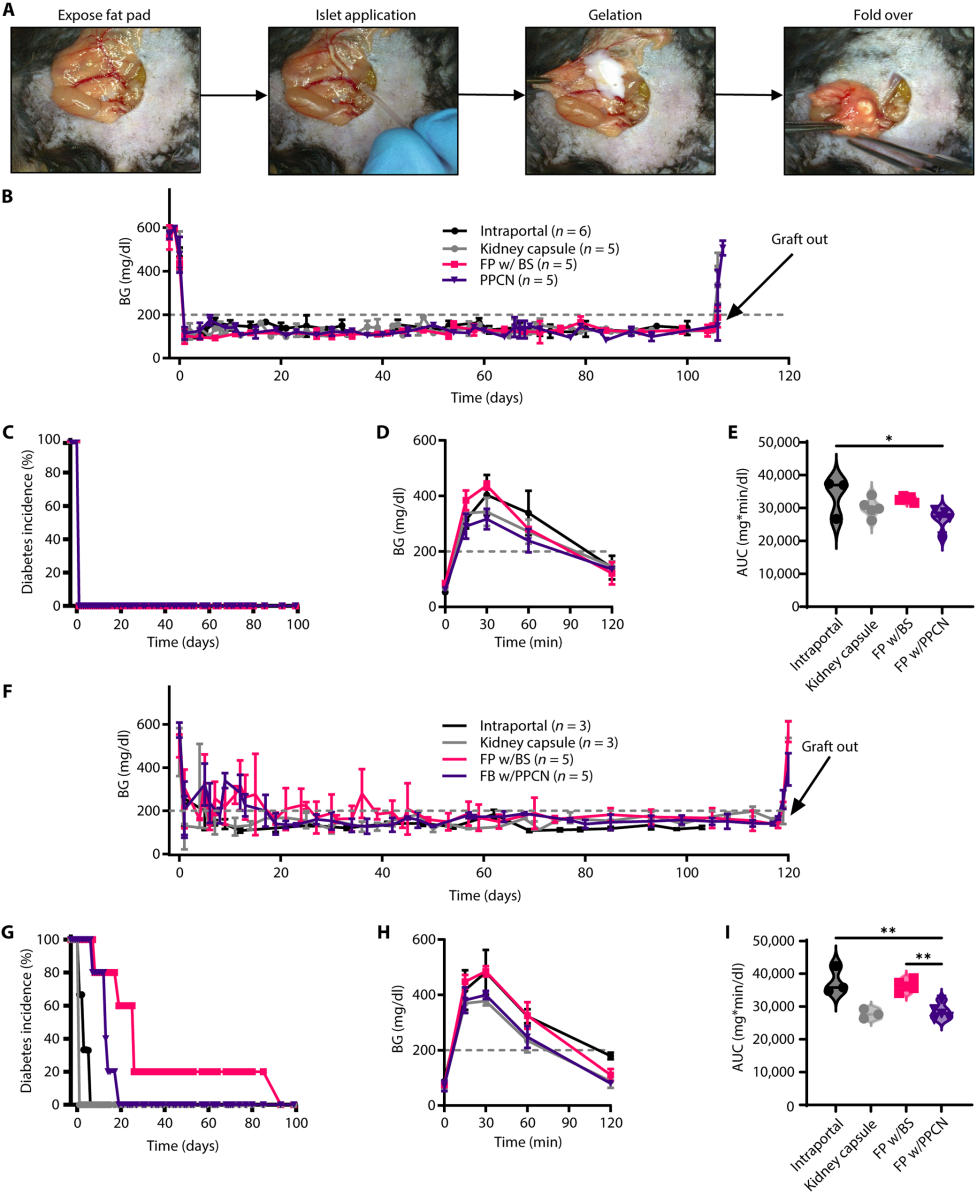
PPCN localizes islets to a target transplantation site and restores normoglycemia with marginal islet mass. (**A**) PPCN facilitates syngeneic (C57BL/6 to C57BL/6) islet transplantation to the mouse fat pad. Digital photos depict the fat pad islet transplant procedure in which from left to right: (i) the fat pad is removed from the peritoneal cavity and laid out flat, (ii) packed islets are applied on a blood vessel, (iii) PPCN is applied and forms a gel to secure the islets to the fat pad, and (iv) the fat pad is folded over upon itself and returned to the peritoneal cavity. (**B**) Nonfasting blood glucose (BG) concentration (in milligrams per deciliter) of mice transplanted with approximately 8200 IEQ/kg body weight (BW) of islets to the liver (intraportal), kidney capsule (KC), or on the fat pad with biologic scaffold (FP w/BS) or PPCN (FP w/PPCN). (**C**) Diabetes incidence (%) (BG concentration greater than 200 mg/dl) by treatment group. (**D** and **E**) Glycemic profile (D) and area under the curve of the profile (AUC; mg * min * dl^−1^) (E) during the intraperitoneal glucose tolerance test (IPGTT) study performed 1 month after the transplantation. (**F**) Nonfasting BG measurements of mice transplanted with a marginal islet mass (approximately 4100 IEQ/kg BW). (**G**) Diabetes incidence (%) (BG concentration greater than 200 mg/dl) by treatment group. (**H** and **I**) Glycemic profile (H) and AUC (I) of the profile during the IPGTT study performed 1 month after the transplantation. All data are presented as means ± SD with *P < 0.05, **P < 0.01 relative to PPCN.

To evaluate whether PPCN could be used to deliver islets to an extrahepatic site, the abdominal fat pad of the mouse was used to mimic islet transplantation to the omentum in humans. This model was selected because both structures are well-vascularized fat tissue located in the intraperitoneal cavity (*36*). A syngeneic C57BL/6 to C57BL/6 mouse model was chosen to mimic autologous islet transplantation and avoid complicating immune responses associated with allogeneic transplant. The key steps for the islet transplantation procedure are summarized in Fig. 4A. Upon application to the fat pad, complete gelation of the PPCN occurred within seconds of contact with the tissue, securing all the islets on the fat pad. No suturing or tissue glue was required for this step due to the tissue-adhesive nature of PPCN. The entire procedure was accomplished within 5 min. In contrast, BS took longer to solidify via the thrombin crosslinking reaction, making it difficult to control the final location of the islets. Additional control groups were included in the study. Islet transplantation to the kidney capsule (KC) was included as a positive control as it is widely used as an extrahepatic islet transplantation location in small animals. However, KC transplantation is not performed in humans due to anatomical differences (*15*). Furthermore, intraportal islet transplantation was used as an additional positive control, as this is the clinically used site of transplantation in humans. However, with this site, it is not possible to retrieve the transplanted islets while keeping the animal alive to ensure that any changes in blood glucose (BG) are a result of the transplanted islets and not residual pancreatic function.

Diabetes incidence was defined as consecutive (two or more) BG measurements >200 mg/dl. When transplanting 8200 islets equivalent (IEQ) per kilogram body weight (BW) (*37*), animals in all three groups achieved euglycemia the day following the transplantation procedure (Fig. 4, B and C). Euglycemia was maintained in all four groups until day 104 after transplantation, at which point a second survival surgery was performed to remove the transplanted islet graft to confirm the source of insulin production (with the exception of the intraportal group). After recovering from the surgery, hyperglycemia was detected in all animals, confirming that the islets contained within the fat pad (or KC) were responsible for maintaining euglycemia during the course of the study (Fig. 4B).

Intraperitoneal glucose tolerance tests (IPGTTs) were performed 30 days after transplantation. The PPCN group had the lowest mean quantified area under the curve (AUC). A significant difference in AUC was observed between the fat pad with PPCN and intraportal groups using 8200 IEQ islets for transplantation (Fig. 4, D and E). At this islet dose, significance was not achieved between the fat pad with the PPCN group versus the KC group or the fat pad with the PPCN group versus the BS group.

Given the complex morphology of a pancreatitis patient’s pancreas, islet isolation is technically challenging. Thus, the islet yield from a pancreatitis patient is significantly lower compared with a standard donor (*38*). To investigate the ability of PPCN to preserve islet function and maintain normoglycemia under conditions associated with pancreatitis, a marginal mass of islets was transplanted for the subsequent study. When 4100 IEQ/kg BW was used (less than one donor per animal) (*39*), the restoration of normoglycemia took longer as compared to the transplant with 8200 IEQ/kg. Animals transplanted with islets in the liver (intraportal), KC, abdominal fat pad via BS, and fat pad via PPCN achieved sustained euglycemia within an average (mean) of 5, 4, 39, and 16 days, respectively (Fig. 4, F and G). In the fat pad with the PPCN group, diabetes incidences subsided on postoperative days (POD) 12, 13, 17, 19, and 19. In the fat pad with the BS group, reversal occurred later and with more variability; sustained normoglycemia started on POD 15, 19, 31, 39, and 93. An unpaired *t* test on the time to normoglycemia between the fat pad with BS and fat pad with PPCN groups showed a trending significant difference (*P* = 0.1366). IPGTT performed 1 month after transplant shows that animals transplanted with BS had higher BG at the 15-, 30- and 60-min time points, while BG values for the PPCN group were comparable to those of the KC control (Fig. 4H). The AUC for the BS group is significantly larger than those of the PPCN and KC groups (*P* < 0.01) (Fig. 4I). The islet graft was explanted 120 days after transplantation. Upon graft removal, euglycemic animals that had received islets via PPCN or BS to the fat pad or in suspension to the KC all reverted to the hyperglycemia state within 48 hours.

### PPCN reduces inflammation, mitigates DNA oxidative damage, and supports neovascularization of transplanted islets

**Fig. 5.**
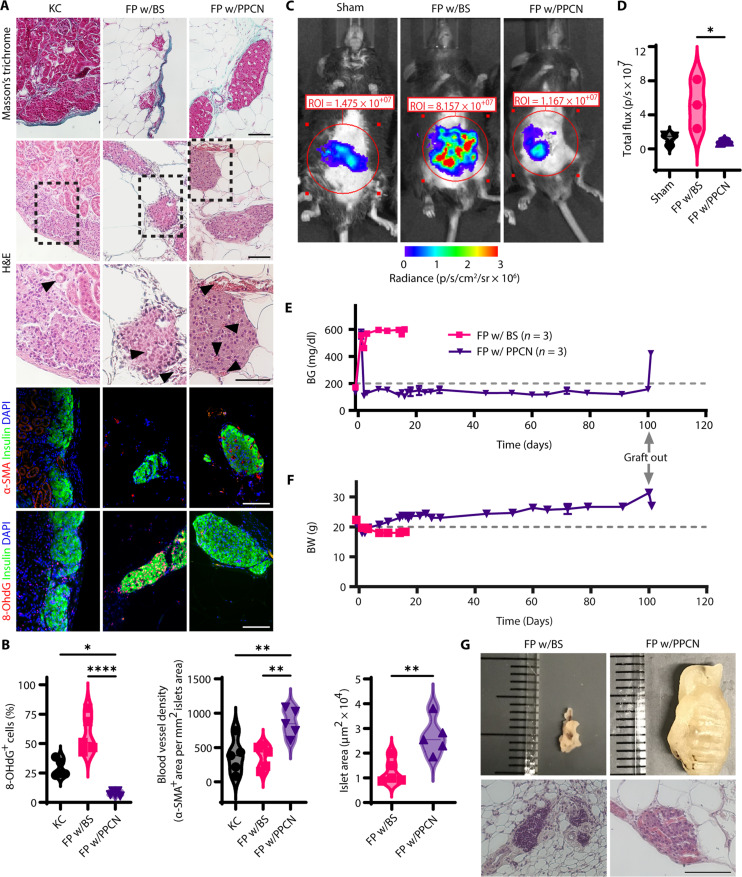
PPCN protects transplanted islets against oxidation-induced DNA damage in vivo. (**A**) Representative histology and immunofluorescence images of islets transplanted to the KC, FP w/BS, or FP w/PPCN, including, from top to bottom: Masson’s trichrome, hematoxylin and eosin (H&E), anti–smooth muscle actin (α-SMA; red), and anti–8-OHdG (red) with anti-insulin (green) and nuclear dye 4′,6-diamidino-2-phenylindole (DAPI; blue) counterstain. For H&E, arrows indicate blood vessels. Scale bars, 100 μm. (**B**) Islet size (by area) in the FP groups (w/BS or PPCN) from H&E images. (**C**) Intra-islet vascular density (α-SMA^+^ structures) in immunofluorescence images. (**D**) Nuclear oxidation (8-OHdG^+^ cells) in immunofluorescence images. (**E** and **F**) Digital images (E) and quantification (F) of reactive oxidative and nitrogen species in vivo 24 hours after transplantation as measured via IVIS by the total flux of L-012 activity. (**G** to **I**) To evaluate protective antioxidative effects ex vivo, islets were prestressed with 10 μM H_2_O_2_ for 5 min in vitro before transplantation. (G) Nonfasting BG concentration (mg/dl) of mice transplanted with ~8200 IEQ/kg BW of prestressed islets to the FP w/BS or PPCN. (H) BW of mice transplanted with ~8200 IEQ/kg BW of prestressed islets to the FP w/BS or PPCN. Mice in the FP w/BS group were euthanized on day 18 after transplantation due to severe weight loss. (I) Digital images (top) and H&E histology (bottom) of the fat pad explanted at 18 days for the BS group or 100 days for the PPCN group. Scale bar, 100 μm. All data are presented as means ± SD with *P < 0.05; **P < 0.01; ****P < 0.0001 relative to PPCN. Statistical significance was determined by *t* test (B) or one-way ANOVA with Tukey’s multiple comparisons test [(C), (D), and (F)] (*n* = 3 to 5).

Histological and immunofluorescence staining was performed on the explanted islet grafts (fat pad and KC). Masson’s trichrome (MT) and hematoxylin and eosin (H&E) staining were used to assess collagen production and islet graft morphology (Fig. 5A). Antibody probes against insulin and α–smooth muscle actin (α-SMA) confirmed the production of insulin, the presence of intact islet structures, and intra-islet neovascularization in the transplanted islets (Fig. 5A). Both BS and PPCN were completely absorbed, leaving islets surrounded by native adipose tissue. Few collagen fibrils and inflammatory cells were observed in the MT-stained sections at the transplant site, suggesting the absence of BS- or PPCN-induced chronic foreign body response. The favorable islet engraftment due to PPCN is supported by the larger size of the islets relative to islets transplanted using BS as quantified by H&E staining (Fig. 5, A and B). There was significantly (*P* < 0.05) more vascularization throughout the islets engrafted using PPCN relative to BS according to α-SMA staining (Fig. 5, A and C). Because increased oxidative stress has previously been reported to be closely associated with pancreatitis-related islet damage (*13*, *40*, *41*), costaining of the 8-OHdG marker was conducted to evaluate oxidation-induced DNA damage in the islet grafts (Fig. 5A). The expression of 8-OHdG was significantly higher in islets engrafted in the fat pad using BS, whereas no signal was observed from islets engrafted with PPCN (Fig. 5D). Although many 8-OHdG–positive cells were also observed in the KC group, unlike the BS group, the majority of the 8-OHdG–positive cells in the KC group were present in the native kidney tissue and not in the grafted islets (Fig. 5A).

Given that it has been reported that initial oxidative stress on the islets has been shown to negatively affect engraftment (*13*), we assessed reactive oxidative and nitrogen species 24 hours after transplantation, in real time, using an L-012 probe (*34*). Analysis by IVIS revealed that islet transplantation with BS caused a significant (*P* < 0.05) increase in reactive species at the site of transplantation, whereas reactive species in the PPCN treatment group resembled the sham condition (Fig. 5, E and F). Animals that received islets transplanted via PPGN, the non-antioxidant version of PPCN, exhibited higher oxidative stress within the fat pad than those that received islets using PPCN (fig. S8).

To further evaluate PPCN for the protection of islets against oxidative tissue damage in vivo, freshly isolated islets were exposed to H_2_O_2_ in a cell culture medium, at a concentration (10 μM) mimicking that secreted by pancreatitis-induced acinar cells (*35*). After 5 min of H_2_O_2_ exposure, the H_2_O_2_-containing medium was removed, and the islets were transplanted into the fat pad of syngeneic hyperglycemic recipient mice that had previously undergone chemical [streptozotocin (STZ)] pancreatectomy. Nonfasting BG levels of these graft recipients were closely monitored before and after the transplantation. Although no significant morphological changes and oxidation damage were observed after 5 min of exposure to H_2_O_2_ according to in vitro culture studies, the in vivo performance of these H_2_O_2_-exposed islets is significantly different (Fig. 5G). Euglycemia was established in the recipients that received islets entrapped using PPCN within 24 hours after transplantation, similar to the mice that received 8200 IEQ/kg BW (Fig. 4A). In contrast, all animals with BS islets grafts exposed to H_2_O_2_ remained hyperglycemic and had to be euthanized 15 days afer transplantation due to significant weight loss (Fig. 5, G and H). A marked difference in the tissue volume at the transplant site was also observed at the time of graft removal. Islets grafted using PPCN were approximately 10 times the size of the islets grafted using BS (Fig. 5I). These results highlight the destructive effects of oxidative stress on the in vivo function of islets posttransplantation and PPCN’s capacity to protect islets against oxidative damage and associated loss of function.

### PPCN is well tolerated, does not elicit a deleterious foreign body response, and is resorbed when applied to the omentum of NHPs

**Fig. 6.**
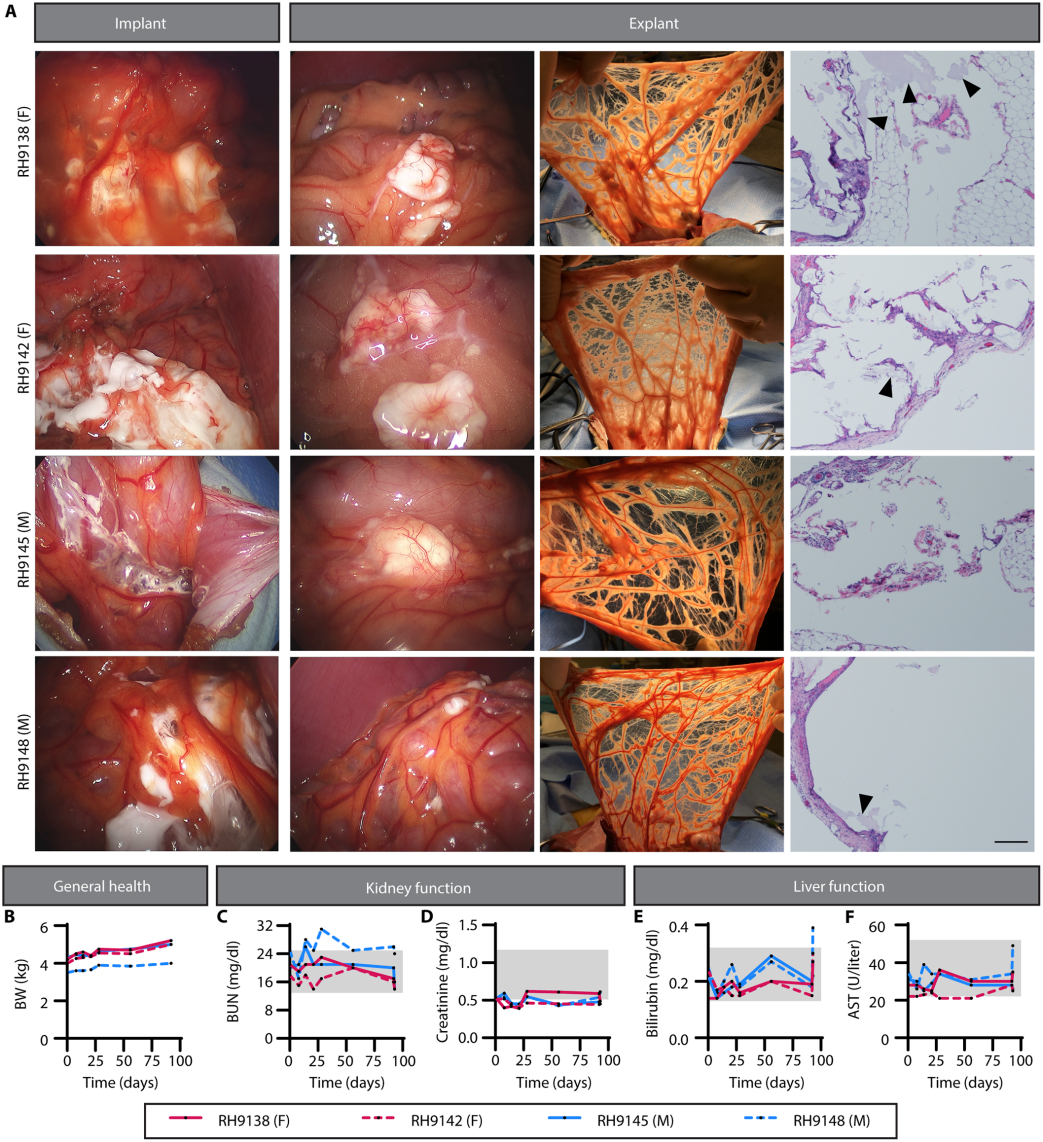
PPCN is biocompatible with the nonhuman primate omentum. Approximately 4 ml of PPCN was implanted via laparotomy in the omentum of each of the rhesus macaque NHPs [*n* = 4; two female (F), two male (M)]. Two batches of PPCN were used. Batch A was implanted in RH9138 (F) and RH9145 (M). Batch B was implanted in RH9142 (F) and RH9148 (M). Explantation occurred approximately 3 months after implantation. (**A**) Implant: Laparoscopic digital photos of PPCN implantation in the omentum of four rhesus macaques (two female, two male). Explant: Left: Laparoscopic digital photos of PPCN within the omentum of four rhesus macaques (two female, two male) approximately 3 months after implantation. Middle: Digital photos of the omentum upon explanation. Right: H&E histology of explanted omentum following 3-month implantation of PPCN. Scale bar, 200 μm. (**B** to **F**) Assessment of general health, kidney function, and liver function in NHPs with omentum PPCN implant. (B) BW (in kilograms) was used to assess general health. [(C) and (D)] Kidney function was monitored via blood urea nitrogen (BUN; in milligrams per deciliter) (C) and creatinine (in milligrams per deciliter) (D) concentration in serum. [(E) and (F)] Liver function was assessed via bilirubin (in milligrams per deciliter) (E) and aspartate aminotransferase (AST; in units per liter) (F) concentration in serum. Day 0 values were measured preoperatively and served as a baseline. Gray boxes represent the normal ranges for each parameter. See tables S2 to S5 for complete blood counts, blood chemistries, urinalysis, and body temperature for all animals over the course of the study.

Unlike the abdominal fat pad found in small rodents, the omentum in large animals such as NHPs and humans is a natural defense mechanism for the pathophysiology of intra-abdominal diseases due to its well-vascularized structure and angiogenic properties (*36*). The pro-inflammatory environment of the omentum in a human could affect islet function; hence, the compatibility of the scaffold with this tissue is of utmost importance (*22*). The similarity in islet architecture and function, as well as the size and anatomy of the omentum between humans and NHPs, motivated us to investigate the tissue response to PPCN in NHPs, specifically rhesus macaques (*42*). PPCN application to the omentum via laparotomy is shown in Fig. 6A. PPCN can easily be applied as a liquid through syringes and rapidly transitions into an opaque hydrogel within seconds upon contact with the tissue at body temperature. The graft was secured and covered with surrounding omentum tissue without the need for sutures or staples. Over the course of the 3-month study, the health of the animals, including disposition, BW, complete blood count (CBC), and chemistry, was carefully monitored. BW was maintained or increased (Fig. 6B). Kidney health, as assessed by blood urea nitrogen (BUN) and creatinine concentrations, showed no sustained impairment in function (Fig. 6, C and D). Similarly, liver function, as measured by bilirubin and aspartate aminotransferase (AST), was maintained throughout the study (Fig. 6, E and F). CBCs, blood chemistries, urinalysis, and body temperature for all animals over the course of the study are documented in tables S2 to S5. At approximately 3 months after implantation, the implantation site was surgically accessed and inspected for any signs of inflammation or a foreign body response to the PPCN (Fig. 6A, explant left). Internal inspection of the implant via laparoscopic camera was normal. In all animals, a small amount of white matter that appeared to be remaining PPCN (~20% of originally applied material) was observed. The entire omentum was extended from the body cavity for gross inspection (Fig. 6A, explant middle). No signs of a foreign body response were noted. The entire omentum was explanted for histopathology. The histopathology report confirmed the absence of inflammation, fibrosis, or tissue abnormalities, except for a few areas that showed signs of remaining PPCN. Tissue composition, including vascularization, also appeared normal (Fig. 6A, explant right). In all, PPCN applied to the omentum was well tolerated by large NHPs, as the animals maintained their baseline health throughout the study with no changes in behavior, blood parameters, or omental inflammation.

### PPCN enables islet function following autologous islet transplantation to the omentum of NHPs

**Fig. 7.**
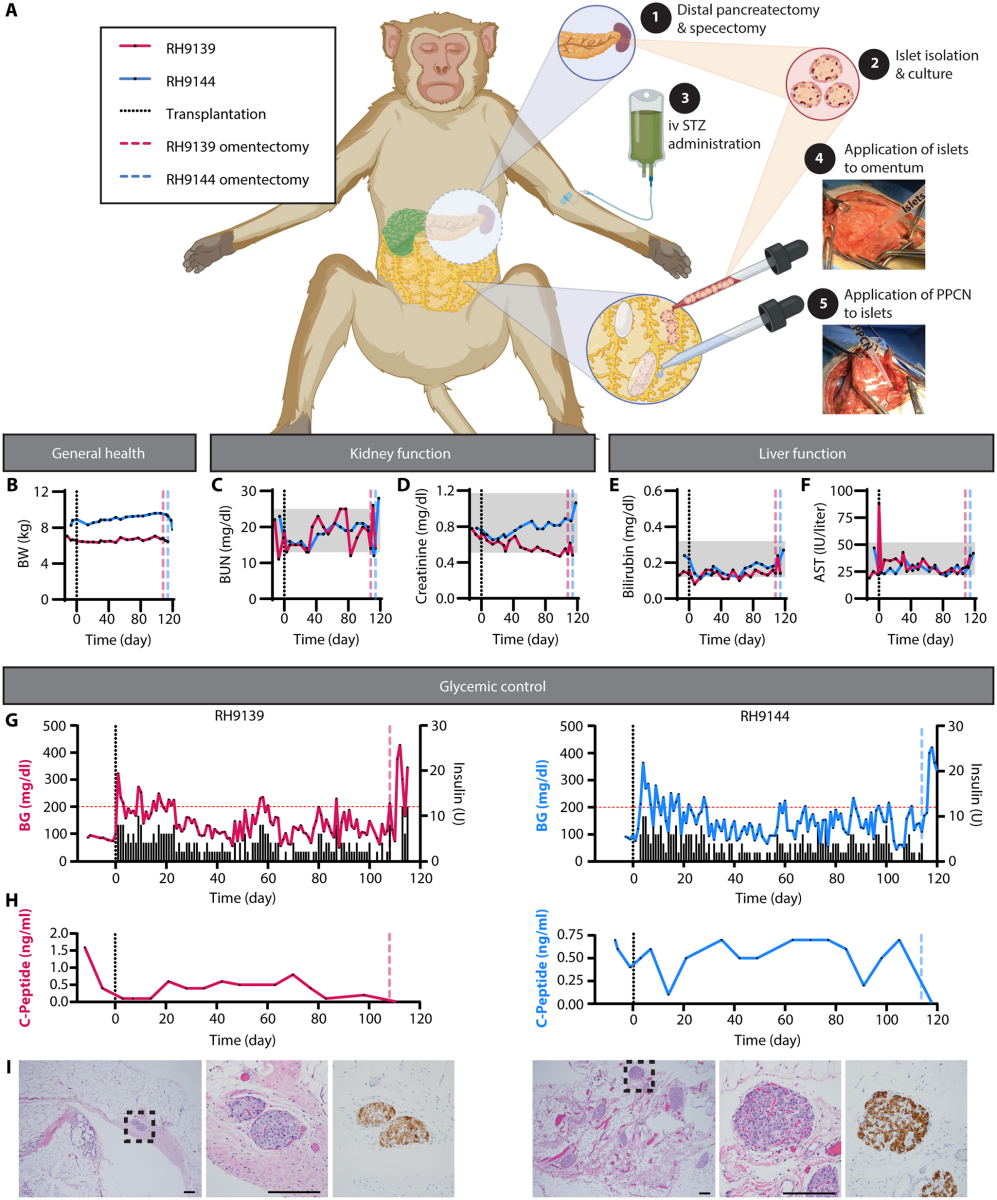
PPCN enables autologous omentum islet transplantation in a nonhuman primate model. (**A**) Schematic and digital photos detailing distal pancreatectomy-autologous islet transplantation to the omentum using PPCN in rhesus macaques [*n* = 2; 1 female (F), 1 male (M)]. On POD −1, to ensure complete beta cell destruction, a distal pancreatectomy was performed followed by intravenous (iv) STZ (80 mg/kg). Islets were isolated and cultured overnight. On POD 0, autologous islet transplantation was performed via laparotomy in the omentum using approximately 4 ml of PPCN. NHPs were monitored for 110 to 120 days. BG was monitored via continuous glucose monitor and/or BG meter. Insulin glargine was given on a sliding scale based on BG. (**B** to **I**) Assessment of general health, kidney function, and liver function in NHPs that underwent TP-IAT in the omentum with PPCN, including BW (in kilograms) (B), BUN (in milligrams per deciliter) (C), creatinine (in milligrams per deciliter) (D), bilirubin concentration (in milligrams per deciliter) (E), and AST (in units per liter) (F) in serum. Day −7 values (for RH9139) and −6 values (for RH9144) served as a baseline. Gray boxes represent the normal ranges for each parameter. (G) Average (daily) BG (in milligrams per deciliter; left axis) and daily exogenous insulin glargine (in units) requirements (right axis) over the course of the study. (H) C-peptide concentration in whole blood (in nanograms per milliliter; left axis) throughout the study. The vertical black dotted lines at day 0 indicate the time of islet transplantation. The colored vertical dashed lines indicate the time of omentectomy, for each NHP. (I) H&E (left and middle: scale bars, 200 μm) and anti-insulin immunohistochemistry (right) staining of omentum tissue containing embedded islets (from omentectomy). See tables S7 to S9 for complete blood counts, blood chemistries, and urinalysis for both animals over the course of the study.

To model the treatment for CP with TP-IAT in an NHP model (Fig. 7A), first, a distal pancreatectomy was performed, followed by chemical induction of diabetes using STZ (*43*, *44*). Isolated islets were cultured overnight and stained to assess morphology, viability, and purity (fig. S9). The viability was 75 ± 32% for RH9139 and 93 ± 15% for RH9144. Purity was more than 95% for both animals. A GSIS assay was performed. The stimulated index was 1.0 ± 0.1 and 2.5 ± 0.5 for RH9139 and RH9144, respectively (table S6). The transplanted islet dose was 39,473 IEQ and 49,638 IPN (6,073 IEQ/kg) for RH9139 and 68,685 IEQ and 61,811 IPN (7,717 IEQ/kg) for RH9144. H&E staining of the residual pancreatic tissue upon necropsy confirmed the absence of native, endogenous islets (fig. S10). The day after the pancreatectomy, autologous islet transplantation was performed in the omentum via laparotomy using approximately 4 ml of PPCN (Fig. 7A). NHPs were monitored closely for over 100 days. Over the course of the study, the health of the animals, including disposition, BW, blood chemistry, and BG, was carefully monitored. BW was maintained or increased (Fig. 7B). Kidney health, as assessed by BUN and creatinine concentrations, showed no sustained impairment in function (Fig. 7, C and D). Similarly, liver function, as measured by bilirubin and AST, was maintained throughout the study (Fig. 7, E and F). CBCs, blood chemistries, and urinalysis for both animals over the course of the study are documented in tables S7 to S9. BG was monitored (Fig. 7G, left axis) and insulin glargine was given on a sliding scale (Fig. 7G, right axis) as needed. For RH9139, for the first month following transplantation, BG was maintained at 186 mg/dl (mean) with 5.1 U (mean) of insulin administered per day. After 1 month, the average BG decreases to 123 mg/dl (mean) and the exogenous insulin requirement drops to 2.8 U (mean) per day. For RH9144, for the first month following transplantation, BG averages 190 mg/dl (mean) with 5.2 U (mean) of insulin administered. After 1 month, the average BG fell to 134 mg/dl (mean) and the exogenous insulin requirement dropped to 3 U (mean) per day. C-peptide was detectable in both animals throughout the course of the study. Upon a survival omentectomy, C-peptide was no longer detectable, confirming that C-peptide secretion was due to autologous islets transplanted on the omentum and not due to any remaining native, endogenous islets post-pancreatectomy and post-STZ administration (Fig. 7H). Upon explantation, functional insulin-producing islets were found within the omentum of both animals (Fig. 7I).

## DISCUSSION

Since first performed in 1977, TP-IAT has been a final hope for people suffering from CP (*45*). A reduction in pain occurs in 85% of patients following this procedure (*4*, *46*). This procedure has enabled patients with CP to reduce their dependence on narcotics to manage pain and improve their quality of life (*46*). Nonaddictive strategies for pain management are particularly important today with the growing opioid epidemic in the United States (*47*). Despite improvements in islet isolation techniques, long-term graft survival and sustained insulin independence remain challenges with this procedure in the CP population (*6*). Patients with CP have a smaller islet mass (*2*) and the inflamed acinar tissue surrounding the islets secretes H_2_O_2_, promoting islet-damaging oxidative stress (*35*). With intraportal islet transplantation, up to 50 to 80% of islets are lost upon infusion (*9*). Given the reduced islet reserves of patients with CP, additional islet loss often leads to diabetes. Three years after transplant, 70% of patients with TP-IAT require exogenous insulin (*46*). Thus, research into alternative, safe, and efficient extrahepatic transplantation sites that meet the needs of this patient population is warranted and the motivation for our study (*48*).

Our laboratory pioneered the development of citrate-based biomaterials (CBBs) for regenerative engineering and regenerative medicine applications (*25*). These biomaterials, which can be engineered to have tailored mechanical and degradation properties, have been investigated for several applications including the regeneration or reconstruction of cardiovascular, bladder, dermal, and musculoskeletal tissues (*32*, *49*–*57*).

CBBs have reached a major translational milestone as they are used in bioresorbable implantable medical devices recently cleared by the U.S. Food and Drug Administration for use in musculoskeletal surgeries with commercial applications in ankle, knee, and shoulder reconstruction (*57*, *58*). We have developed a temperature-responsive, phase-changing, easy-to-use, citrate-based macromolecule, PPCN, with intrinsic anti-inflammatory and antioxidant properties. We hypothesized that PPCN protects islets isolated from patients with CP against oxidative stress and prolongs their function in vitro and during extrahepatic islet transplantation (Fig. 1A).

The islet isolation process and in vitro cell culture have been shown to expose islets to oxidative stress, both by facilitating the generation of reactive oxygen species (ROS) and by hindering the adaptive up-regulation of cellular antioxidants (*13*). Islets isolated from the patients with CP are subject to ROS secreted by the surrounding inflamed acinar tissue. Furthermore, insulin-producing beta cells have significantly lower levels of antioxidant enzymes catalase, superoxide dismutase, and glutathione peroxidase, which make them particularly vulnerable to oxidative cell damage, especially during ischemia-reperfusion injury (*59*). These findings have therefore prompted studies that use antioxidant peptides and oxygen-generating strategies to improve islet function. An example is the addition of the peptide carnosine during ex vivo islet culture and oxygen-generating polydimethylsiloxane (PDMS)-CaO_2_ scaffolds for islet encapsulation (*60*). Although intriguing in vitro results have been reported, antioxidant scaffold approaches have not been evaluated in vivo for islet transplantation (*61*). One potential explanation for the observed superior performance of PPCN may be its intrinsic antioxidant property, which is due to the polyethylene oxide citrate moieties present within the polymer backbone. Reduced DNA oxidative damage was consistently observed in islets engrafted with PPCN as per the 8-OHdG staining results (Fig. 5A).

To determine whether there is a link between reducing oxidative stress and the preservation of islet viability and insulin secretion function, oxidative stress reporter islets were created using roGFP overexpression (Fig. 3). Intracellularly expressed roGFP has been used in several studies as an effective reporter protein to allow real-time nondestructive monitoring of the cellular redox state (*62*). We hereby report the use of this redox probe technology to evaluate the protective properties of an antioxidant biomaterial. When exposed to H_2_O_2_-induced oxidative stress, representative of CP, both human and mouse islets entrapped in PPCN experienced a significant delay in the progression of oxidation, thereby better-preserving viability, and GSIS response. To further demonstrate the impact of oxidation damage during the ex vivo culture period on islet performance posttransplantation, oxidative stress was induced for 5 min in the same way as the in vitro studies through low-dose H_2_O_2_ treatment before the transplantation. After the transplantation, no sign of islet damage was observed in the PPCN group as euglycemia was achieved the next day after transplantation similar to results from the original study (8200 IEQ/kg). However, in the case of the BS group, hyperglycemia persisted after the transplantation, indicating the complete loss of insulin secretion function in those islets (Fig. 5G). Our results provide compelling evidence regarding the link between oxidative stress in vitro and islet insulin secretion function in vivo and that an antioxidant microenvironment can preserve the insulin secretion function of the isolated islets.

From the clinical perspective, the great omentum is an ideal location for islet transplantation due to its large, well-vascularized area and accessibility via minimally invasive procedures (*6*, *39*). However, since its physiological role involves protecting the peritoneal cavity from invading infectious diseases, the pro-inflammatory environment at the omentum site may result in unexpected severe inflammatory responses toward the transplanted islets and the biomaterial used to deliver them (*22*). To date, several natural and synthetic biomaterials have been investigated as vehicles to facilitate the engraftment of islets in the omentum (*63*). Pedraza *et al*. (*60*) used a PDMS porous scaffold in an STZ-induced diabetic rat model to restore euglycemia with 10,000 IEQ/kg BW (1800 IEQ/rat). However, a fibrous capsule developed around the graft area according to the histology data, due to the foreign body response elicited by the PDMS. Modifications to the PDMS with the angiogenic growth factor platelet-derived growth factor (PDGF-BB) or fibrin gel were able to slightly reduce the number of islets to 8333 IEQ/kg BW (250 IEQ per mouse); however, it took 19 days for the recipient to achieve euglycemia (*64*). Berman *et al*. (*43*) demonstrated the use of a porous polyglactin and poly-p-dioxanone scaffold (Codman Ethisorb Duan Patch) to achieve minimum exogenous insulin requirements (0.3 to 0.4 IU/kg per day) with autologous islets (5093 IEQ/kg) transplanted to the omentum of cynomolgus macaques. Immunofluorescence and histological staining of the explanted islet graft demonstrated elevated host cell infiltration around the graft area. Stendahl *et al.* (*65*) investigated the use of vascular endothelial growth factor (VEGF) and fibroblast growth factor 2 (FGF-2) with heparin-binding peptide amphiphile nanofibers in a poly(l-lactic acid) scaffold. In their study, only 78% of the mice receiving the VEGF/FGF-2–releasing scaffold achieved euglycemia within 54 days after transplantation. Furthermore, bilayered PEG and PEG-VEGF islet encapsulation systems have recently been described (*66*). However, islet transplantation with 17,391 IEQ/kg (4000 IEQ per rat) did not resolve hyperglycemia, indicating insufficient insulin secretion. A 2016 study published by Berman *et al*. (*39*) evaluated an autologous BS hydrogel as a vehicle to deliver islets to the omentum with a proportionally clinically relevant number of islets [8200 IEQ/kg BW (1300 IEQ per rat)] and achieved euglycemia within a week of the transplantation. The authors also evaluated the BS for omentum allogeneic islet transplantation (9347 IEQ/kg) in diabetic NHPs and reported the presence of C-peptide in the blood following transplantation and viable islets within the omentum upon explantation. However, insulin independence was not achieved (*39*). Recently, Deng *et al*. (*44*) reported the achievement of euglycemia in STZ-induced diabetic NHP following allogeneic islet transplantation to the omentum with BS. Although two out of three NHPs achieved exogenous insulin-free normoglycemia, an average of 16,800 IEQ/kg, more than twice a clinically relevant dose, was used in these studies (*44*). To obtain this large IEQ relative to BW, large donors (8.4 to 9.1 kg) and small recipients (5.6 to 6.5 kg) were used. Given that patients with CP must rely on their own islets for TP-IAT and islet yields from these patients are significantly lower than those obtained from donors of islets for allotransplantation (*38*), studies with high IEQ islet transplants relative to BW are not clinically viable option for patients with CP. Therefore, a biomaterial that provides an adequate microenvironment to preserve islet viability and function is required to improve the outcomes of patients with CP who undergo TP-IAT.

To develop a clinically useful material to enable extrahepatic transplantation of pancreatic islets for patients with CP, BS and PPCN were both evaluated in this study. In mice, we evaluated two islet doses: 8200 IEQ/kg BW and 4100 IEQ/kg BW per recipient (Fig. 4). These islet masses represent 50 and 25% of the total islets found in healthy mouse pancreas, the latter being substantial reduction in islet dose when compared to previous reports (*17*, *67*–*69*). For these studies, diabetes incidence was defined as two consecutive BG readings >200 mg/dl. Using 8200 IEQ/kg BW, euglycemia was achieved in both the PPCN and BS groups confirming the noninferiority of PPCN to BS. However, in the marginal islet study, PPCN demonstrated consistent restoration of normoglycemia and superior glycemic control, as indicated by the response to a glucose challenge via the IPGTT. Diabetes reversal occurred in mice transplanted to the fat pad with PPCN between POD 12 and 19, whereas mice transplanted with BS had more variable outcomes, with normoglycemia restored between POD 15 and 93 (Fig. 4G). Delayed insulin response was also observed in the BS group when the transplanted islets underwent a glucose challenge via the IPGTT, indicating insufficient glucose control. Furthermore, unlike scaffolds reported by others, PPCN was completely resorbed by the time of explantation. No cell infiltration or fibrosis was observed around the graft area and islets were incorporated into the surrounding adipose tissue with enhanced intra-islets vasculature (Fig. 5A).

Given the anatomical and immunological differences between mice and humans, we investigated the biocompatibility of PPCN within the NHP omentum. Unlike mice, both NHP and humans are bipeds that stand and walk upright. Furthermore, both species have an omentum with the physiological capability to produce an inflammatory response. The PPCN implantation procedure was performed via laparotomy. PPCN is applied at room temperature as a clear liquid and immediately forms a white gel upon contact with the omentum due to the polymer’s thermoresponsive nature. Using body temperature to gel a macromolecule is advantageous over other methods that use enzyme- or light-activated in situ polymerized scaffolds, as these methods result in insufficient encapsulation due to the uneven polymerization or additional damage to the encapsulated islets as well as the surrounding native tissue (*70*). Procedures that involve long gelation times risk allowing islets to leak into the intraperitoneal cavity. Unlike methods used to date, delivery of PPCN can be easily achieved via an endoscope-enabled procedure. Three months following implantation, approximately 80% of the PPCN was reabsorbed. Signs of systemic inflammation were not evident, as per blood tests and gross examination or histopathology, demonstrating the safety of PPCN when applied to the greater omentum of a large NHP.

Given the promising results of the in vivo biocompatibility studies, we assessed the use of PPCN for IAT in the omentum in NHPs. Total elimination of the resident islets was achieved via distal pancreatectomy, followed by chemical induction of diabetes via IV STZ. This method of pancreatectomy decreased surgical time, as resection from the duodenum was not required while ensuring no viable islets remained. The islet doses of 6073 IEQ/kg for RH9139 and 7717 IEQ/kg for RH9144 align with this reduced yield. Isolated islets were secured onto the omentum using the PPCN gel. One month after the procedure, BG averages of 123 and 134 mg/dl for RH9139 and RH9144, respectively, fall within the normal range for rhesus macaques (58 to 153 mg/dl). Minimal insulin was required to maintain normoglycemia. After the 1-month engraftment period, a mean of 0.41 and 0.32 U/kg per day was required for RH9139 and RH9144, respectively. These results confirm the engraftment of functional islets within PPCN in the NHP omentum.

While other groups have performed autologous islet transplants in NHPs (*71*–*75*), the restrictive nature of NHP research, due to high costs, ethical considerations, regulations, and species-specific surgical knowhow, limits the number of studies and number of animals available to benchmark our results. Furthermore, many NHP islet transplantation studies focus on the application of T1D and thus use allogeneic or xenogeneic islets (*43*, *44*, *71*). In addition, transplant site–specific differences (*71*, *72*, *75*), inconsistencies in the diabetic state (*71*, *72*, *75*, *76*), and nonstandardized methods of reporting islet dose (*73*, *74*) also confound comparison.

Researchers from the University of Pennsylvania Perelman School of Medicine Pancreatic Islet Cell Transplant Program described a partial pancreatectomy and autologous islet transplant to the subcutaneous space of the abdominal wall in two NHPs (*71*, *72*). Of note, for the first animal, STZ treatment was not performed after partial pancreatectomy; thus, some functional islets likely remained within the residual pancreas. This NHP received 24,000 IEQ/kg, had a mean BG of 48 mg/dl at 2 months post-op, and did not require exogenous insulin. While this animal demonstrated exogenous insulin-independent glycemic control, its outcomes cannot be properly compared to those of our study due to the large number of islets used in the subcutaneous engraftment site relative to the islet number we used for the omentum engraftment site (~24,000 versus ~6900 IEQ/kg). In addition, the presence of residual native islets due to the partial pancreatectomy without STZ further complicates comparisons to our study. The second NHP from University of Pennsylvania study provides a better comparison to our NHP omentum islet engraftment via PPCN study as STZ was given following partial pancreatectomy, and a smaller islet dose was used by the researchers (~12,000 IEQ). At 2 months after transplantation, they reported that the NHP had a mean BG of 234 mg/dl with exogenous insulin requirements of 2 U per day (0.5 U/kg per day). Therefore, islet transplant to the omentum using PPCN seems advantageous as a much lower dose of islets results in better BG control with lower exogenous insulin requirements (islet dose: 12,000 IEQ/kg versus ~6900 IEQ/kg, BG: 234 mg/dl versus 123 to 134 mg/dl, and exogenous insulin requirements: 0.5 U/kg per day versus 0.32 to 0.41 U/kg per day for the subcutaneous space islet transplant and PPCN-mediated omentum islet transplantation, respectively).

Although we have demonstrated the translational potential of PPCN as an additional treatment tool for patients with CP, there are limitations to our study. Our animal models focus on the impact of CP on the islets and do not reflect the systemic inflammation that patients with CP undergo after years of living with this disease. Generally, patients with CP experience elevated inflammatory cytokines in their blood, including interleukin-1β (IL-1β), IL-6, IL-8, interferon-γ (IFN-γ), macrophage inhibitory cytokine, neutrophil gelatinase-associated ligand, transforming growth factor–β (TGF-β), and tumor necrosis factor–α (TNF-α) (*77*, *78*). This inflammation likely plays a role in islet engraftment and potentially contributes to the negative outcomes of TP-IAT experienced by patients with CP. Future work should also focus on the development of models of CP that can be used to test clinically relevant approaches for CP therapies. One may consider the use of an in vitro model with elevated cytokine concentrations mimicking that of a patient with CP. For in vivo work, important considerations include the use of upright animals and chemical or surgical induction of CP that accurately models pancreatic inflammation, islet loss, and systemic inflammation. Furthermore, consideration must be given to current surgical practices for TP-IAT. In the clinical setting, pancreatectomy, islet isolation, and intraportal infusion all occur on the same day within the span of a few hours (*1*, *3*, *5*, *7*, *8*, *38*, *46*, *79*). The islets are minimally purified and infused with acinar tissue. For the NHP model presented herein, due to the need to give STZ to establish TP, an overnight washout period is required to ensure that the STZ will not damage the transplanted islets (*71*, *72*). Thus, the partial pancreatectomy, islet isolation, and STZ treatment occur on the first day and the transplantation occurs the following day. Following isolation, the islets are fully purified, removing acinar tissue, and then cultured overnight. Modification of the NHP model or clinical practices may be required to ensure consistency. To facilitate the clinical use of PPCN in TP-IAT, a head-to-head study comparing intraportal islet delivery and PPCN-mediated omentum islet transplantation should be conducted in NHPs using the same islet dose and clinical standards. Given our results in mice and NHPs that demonstrate improved islet transplantation outcomes using PPCN and the practical and biological challenges with intraportal islet delivery such as liver thrombosis and IBMIRs, phase-changing antioxidant biomaterials such as PPCN can play an important role in islet function preservation, extrahepatic engraftment, and improving the quality of life of patients with CP.

## MATERIALS AND METHODS

### Study design

The overall objective of this study was to assess the ability of PPCN to preserve the viability and function of insulin-producing islets in vitro and in vivo, under oxidative stress conditions associated with CP. To achieve this goal, first, we performed in vitro studies with murine and human islets. Islets were encapsulated in PPCN or control materials before culture. We used Live/Dead staining and GSIS assay to measure viability and function, respectively. Transduction of murine and human islets with an oxidation state reporter enabled us to assess redox status over time as a function of culture conditions. Next, we assessed PPCN in an STZ-induced syngeneic islet transplant murine model. PPCN was used to secure islets to the mouse fat pad. Evaluation of PPCN was assessed upon restoration of normoglycemia, response to a glucose challenge, and terminal histopathology. PPCN-enabled islet transplant to the murine fat pad was compared against fat pad transplant with BS, KC, and intraportal islet transplant using various islet does and oxidative stress conditions. Biocompatibility of PPCN was evaluated in the omentum of four NHPs (two female, two male) for 3 months. Outcomes were assessed on the basis of BW, CBC, white blood cell differential, blood chemistry, urinalysis, gross observation upon explantation, histopathology upon explantation, and full necropsy. To mimic a TP procedure, we performed a distal pancreatectomy followed by chemical induction of diabetes via STZ. Islets were isolated, cultured overnight, and transplanted to the omentum of two NHPs with PPCN (one female, one male). NHPs were monitored for over 100 days. BG was assessed daily. Insulin was given on a sliding scale. Outcomes were measured using the same criteria as for the biocompatibility studies with the addition of normoglycemia, insulin requirements, intravenous dextrose tolerance test (IVDTT), and glucagon test. Histopathology, CBCs, blood chemistry, and urinalysis were conducted in a blinded manner.

### Human tissue

Human islets were obtained from Northwestern University Human Islet Transplant Program (Institutional Review Board exemption: STU00207825) or from Prodo Laboratories Inc. All human tissue was obtained with informed consent from the patient or their family.

### Animals

Eight- to 12-week-old, male C57BL/6 mice were purchased from The Jackson Laboratory. Mice were housed in the Center for Comparative Medicine at Northwestern University. Northwestern University’s Institutional Animal Care and Use Committee (IACUC) approved all animal protocols.

Four- to 10-kg rhesus macaques were purchased from approved animal vendors. Animals were negative for herpes B, tuberculosis, simian immunodeficiency virus, simian retrovirus, and simian T-lymphotropic virus. All animal protocols were approved by University of Illinois Chicago and Northwestern University’s IACUC.

### Materials

All chemicals used in the study including citric acid, PEG, glycerol 1,3-diglycerolate diacrylate, pNIPAAm, collagenase (type XI), dextran, and thrombin from murine plasma were purchased from Sigma-Aldrich.

### PPCN synthesis, characterization, and solution preparation

PPCN was synthesized from citric acid, PEG, glycerol 1,3-diglycerolate diacrylate, and pNIPAAm following the previously published method (*25*). The resulting PPCN was characterized via ^1^H-NMR and ATR-FTIR, neutralized to pH 7 with sodium hydroxide, sterilized with ethylene oxide gas sterilization, and properly vented before use. A PPCN solution (100 mg/ml) was made by dissolving lyophilized PPCN in sterile PBS.

### Murine islet isolation

Mice were first anesthetized with an intraperitoneal injection of ketamine and xylene. After a midline abdominal incision, cold collagenase solution was injected into the pancreas via the cannulated bile duct. The collagenase-infused pancreas was then dissected and incubated at 37°C for 15 min. After the digestion, the large undigested connective tissue was removed by passing the digested pancreas through a mesh screen. The filtrate was then applied to a discontinuous dextran gradient to separate islets from the remaining connective tissue fragments. After two gradient washes, the purified islets were hand-picked and counted under the microscope. All mouse data are obtained from at least three different islet isolations.

### In vitro islet entrapment, viability, and insulin secretion study

Islets were purified, counted, and sorted into cell strainers (10 IEQ per strainer). Cell culture media were drained from the cell strainer immediately before adding a gel-forming solution to the islets. For pNIPAAm PPCN treatments, 40 μl of room-temperature liquid was applied. For the BS group, blood was collected from syngeneic (autologous) donor mice in sodium citrate tubes on the morning of the procedure. The blood was spun down at 1500*g* for 10 min and plasma was collected. Islets were dispersed with 20 μl of autologous plasma to mimic the “islet/plasma slurry” described in (*39*). Thrombin (Sigma-Aldrich) was dissolved in Dulbecco’s PBS with Ca^2+/^Mg^2+^ (Gibco) at 1000 U/ml (*18*, *39*). Thrombin solution (20 μl) was layered on the islet/plasma slurry to form a gel (*18*, *39*). The total volume of PPCN and BS were both 40 μl. The islets from all treatment groups were then incubated at 37°C for 5 min to allow the thermoresponsive gels (pNIPAAm or PPCN) to solidify before culture media were added to the wells. After the culture medium was added, all plates were returned to the 37°C incubator.

The viability of the islets was assessed after 24 days of incubation using two different methods: the resazurin assay for quantification (Sigma-Aldrich) and the Live/Dead assay for visualization (Life Technologies). Both assays were performed following the manufacturer’s protocol.

Low (2.8 mM) and high (28 mM) glucose solutions were prepared in Kreb’s buffer for the GSIS test. The concentrations were determined on the basis of the NIH human islets standard operating procedure. Briefly, after the removal of growth media from the encapsulated islets, the islets were first washed with the low glucose solution and then sequentially incubated in (i) low glucose equilibration solution, (ii) low glucose solution, and (iii) high glucose solution for 1 hour each. After the incubation, the solution from (ii) and (iii) were collected and measured using an insulin ELISA kit (Thermo Fisher Scientific for mouse islets, and Mercodia for human islets). The stimulation index was defined as the ratio of stimulated (high glucose) to baseline (low glucose) insulin secretion.

The test was done by comparing the different amounts of insulin secreted by the islets when subject to low (2.8 mM) and high (28 mM) glucose concentrations. The results were reported as stimulation index, which is acquired by dividing the amount of insulin produced by the islets in the high glucose solution by the insulin amount produced in the low glucose solution. Secreted insulin concentrations in response to the low and high glucose environments can be found in figs. S4 to S7.

### roGFP transduction and oxidation inhibition study

To access the oxidation status of the islets under varied culture conditions, freshly isolated islets were treated with engineered lentivirus encoding the roGFP gene. roGFP expression was monitored using a fluorescent microscope after the transduction. The expression level of the roGFP protein was monitored for 96 hours after the transduction of the roGFP viral vector. The reduced protein signal (488 nm) was observed to be gradually increasing and reached a maximum at 72 hours, while the oxidized protein signal remained at a minimum level at that point. Once the roGFP protein expression reached its maximum in the viral vector-treated islets, these roGFP-islets were either cultured in standard media suspension, BS, pNIPAAm homopolymer, or PPCN.

The oxidation inhibition study was carried out in 15-well glass bottom slides (ibidi). roGFP islets were split into each well before treatment with various conditions (PPCN, pNIPAAm, or BS). Baseline (0 min) confocal images of the islets under each condition were taken with two excitation wavelengths (405 and 475 nm) and one fixed emission wavelength of 509 nm. Hydrogen peroxide was then added to the islets culture with a final concentration of 10 μM. The oxidation status of the islets was monitored under confocal microscopy at each time point. The oxidation percentage was quantified on the basis of the fluorescent intensity under the two wavelengths using ImageJ.

### Murine syngeneic islet transplantation

Recipient animals were pre-treated with intraperitoneal STZ (190 mg/kg) 1 week before transplantation to induce diabetes. Diabetes was confirmed by BG > 400 mg/dl before transplantation (*80*). Three different transplant locations were applied in the study. The abdominal fat pad is used for BS and PPCN islet transplantation. Intraportal islet transplantation to the liver and KC islet transplantation were used as positive controls. All procedures were conducted following previously published procedures (*17*, *39*, *80*). For the abdominal fat pad model, methods were based on the work of Berman *et al *(*39*). A small incision was first created on the abdominal part of the animal to expose the abdominal fat pad. The fat pad was laid out flat. Packed islets were applied along a blood vessel, as shown in Fig. 4A. For the BS group, blood was collected from syngeneic (autologous) donor mice in sodium citrate tubes on the morning of the procedure. The blood was spun down at 1500*g* for 10 min and plasma was collected. Islets were dispersed with 20 μl of autologous plasma to mimic the “islet/plasma slurry” described in (*39*). Thrombin (Sigma-Aldrich) was dissolved in Dulbecco’s PBS with Ca^2+/^Mg^2+^ (Gibco) at 1000 U/ml (*18*, *39*). Thrombin solution (20 μl) was layered on the islet/plasma slurry to form a gel, adhering the islets to the omentum, as described in (*39*) and (*18*). For the PPCN group, 40 μl of PPCN was applied, forming an opaque gel, and securing the islets into the omentum (Fig. 4A). For both groups, once islets were secured in place, the fat pad was folded over onto itself as depicted in (*39*) (Fig. 4A, right). For consistency between treatment groups, no sutures or gel was used to seal the fat pad. The folded fat pad was returned to the intraperitoneal cavity. After the transplantation surgery, the nonfasting BG of the animals was monitored daily for the first 2 weeks after surgery, and then once a week afterward. Diabetes incidence was defined as consecutive (two or more) BG readings >200 mg/dl. At the end of the study, the graft was explanted via a survival surgery. Animals were allowed to recover from the surgery, and their BG levels were monitored for another 48 hours, after which time the mice were euthanized.

### Murine intraperitoneal glucose tolerance testing

IPGTT was performed 1 month after transplantation. The animals were fasted for 16 hours before receiving an intraperitoneal injection of 2 g/kg BW of 50% dextrose (Abbott Laboratories) solution. BG was measured at 0, 15, 30, 60, and 120 min after the glucose injection.

### Tissue collection and immunofluorescent staining

Islets containing fat pad and kidney were harvested and processed for paraffin sectioning. Immunofluorescent staining for blood vessels, insulin, cell death, and oxidation marker (8-OHdG) was performed following the manufacturer’s protocol. Digital images were acquired with a Nikon fluorescent microscope. Images were then processed with ImageJ.

### Biocompatibility and safety of PPCN in the omentum of NHPs

Rhesus macaques were placed under general anesthesia. PPCN (4 ml) was applied to the greater omentum via laparotomy. Body temperature and weight, CBCs, white blood cell differential, and blood chemistry were measured before and at several intervals, after the surgery, until euthanasia to assess for infection, liver function, kidney function, and blood composition. At approximately 90 days after implantations, the animals were euthanized. A full necropsy was performed. The Veterinary Diagnostic Laboratory at the College of Veterinary Medicine at University of Illinois Chicago performed histology. A full histopathology report was provided.

### Autologous islet transplantation to the omentum using PPCN in NHPs

Baseline BG, IVDTT, glucagon test, CBC, white blood cell differential, blood chemistries, urinalysis, body temperature and weight, and serum C-peptide were performed on rhesus macaques (*n* = 2; one female, one male). NHPs were placed under general anesthesia, and a distal pancreatectomy was performed, followed by intravenous (iv) administration of STZ (80 mg/kg). The islets were isolated and purified using previously established methods. Following overnight culture, islets were counted and assessed for viability using fluorescein diacetate/propidium iodide, purity using dithizone staining, and function via GSIS assay (fig. S9 and table S6). Under anesthesia, the purified islets were transplanted via laparotomy to the omentum on NHPs. Approximately, 4 ml of PPCN was applied to secure the islets into place on the omentum. BG was monitored daily using an AccuCheck Guide BG meter or FreeStyle Libre2 continuous glucose monitor. Insulin glargine (Lantus, Sanofi, 100 U/ml) was administered on a sliding scale twice per day. Baseline assessments were repeated at regular intervals throughout the study. At over 100 days after transplant, the animals were euthanized. A full necropsy was performed. The Veterinary Diagnostic Laboratory at the College of Veterinary Medicine at University of Illinois Chicago performed histology, including anti-insulin immunohistochemistry on residual pancreas tissue to confirm beta cell depletion (fig. S10). A full histopathology report was provided.

### Statistical analysis

Statistical analyses were performed using GraphPad Prism 6, and two-way analysis of variance (ANOVA) was used to measure differences for experiments with multiple data sets with a Tukey test performed between groups with significant differences to correct for the multiple pair-wise comparisons. A value of *P* ≤ 0.05 was considered to be statistically significant. Values are reported as the means ± SD.
