# Supplementary material for: Phase-changing citrate macromolecule combats oxidative pancreatic islet damage, enables islet engraftment and function in the omentum
Source: Sci Adv. 2024 Jun 7;10(23):eadk3081. doi: 10.1126/sciadv.adk3081 (PMC11160476; doi:10.1126/sciadv.adk3081)
Supplement: Supplementary file 1 — Figs. S1 to S10 Tables S1 to S9 [file sciadv.adk3081_sm.pdf]

Science Advances
AAAS

Supplementary Materials for

Phase-changing citrate macromolecule combats oxidative pancreatic islet damage, enables islet engraftment and function in the omentum

Jacqueline A. Burke *et al.*

Corresponding author: Guillermo A. Ameer, g-ameer@northwestern.edu

Sci. Adv. **10**, eadk3081 (2024)
DOI: 10.1126/sciadv.adk3081

This PDF file includes:

Figs. S1 to S10
Tables S1 to S9

Supplementary Figures

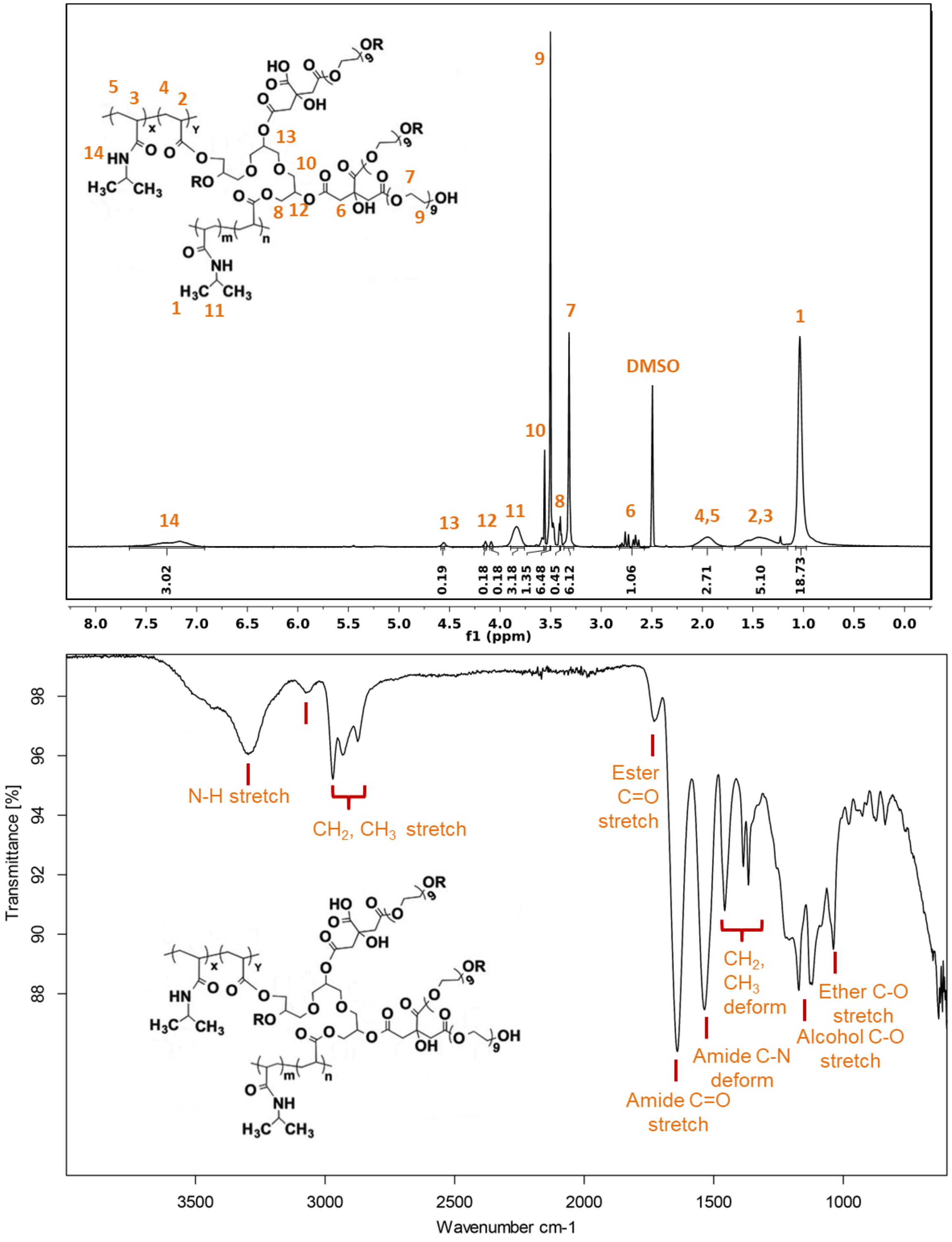

Fig. S1. ^{1}H-NMR and ATR-FTIR spectra confirm the formation of poly(polyethylene glycol citrate-co-N-isopropylacrylamide) (PPCN).

(Top) Proton nuclear magnetic resonance spectroscopy (^{1}**H-NMR**); (Bottom) Attenuated total reflection Fourier transform inferred spectroscopy (**ATF-FTIR**).

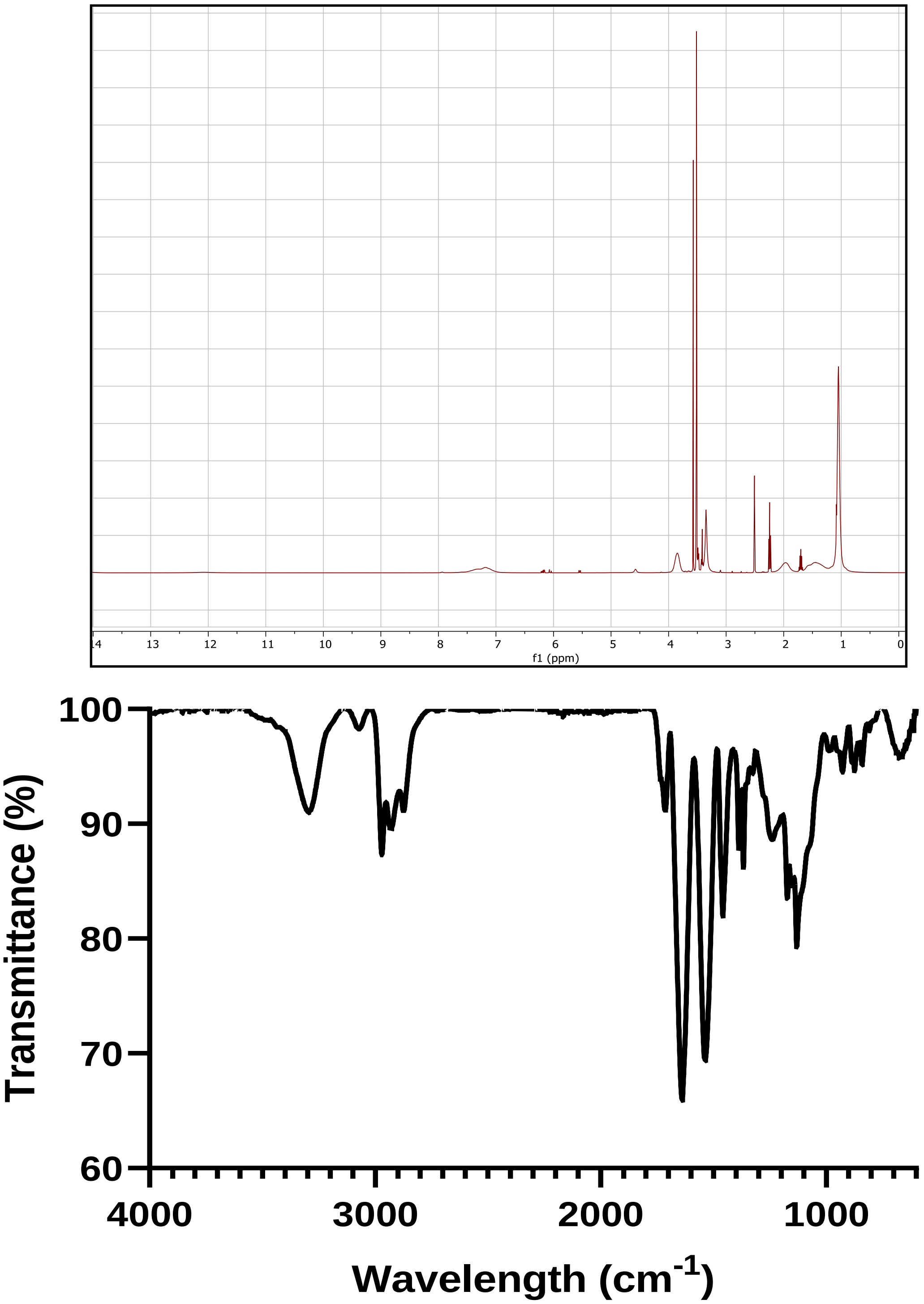

Fig. S2. ^{1}H-NMR and ATR-FTIR spectra confirm the formation of poly(polyethylene glycol glutarate-co-N-isopropylacrylamide) (PPGN).

(Top) Proton nuclear magnetic resonance spectroscopy (**^{1}H-NMR**); (Bottom) Attenuated total reflection Fourier transform inferred spectroscopy (**ATF-FTIR**).

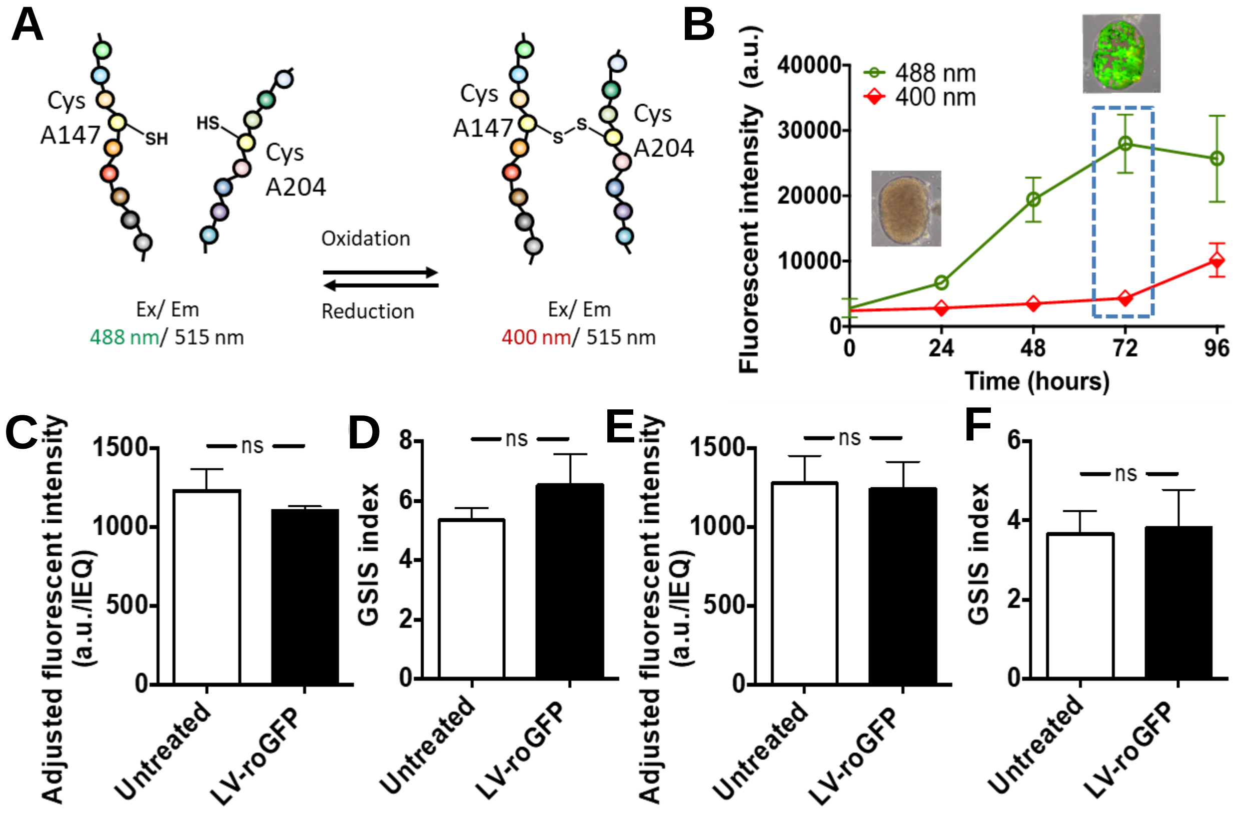

Fig. S3. Overexpression of roGFP does not affect islet viability and insulin secretion function.
(**A**) Schematic of the transition between the reduced and oxidized forms of roGFP. (**B**) The fluorescence intensity of reduced and oxidized RoGFP signals over time (insert: RoGFP overexpressing islets at time 0 and time 72 hours after the addition of the viral vector). **(C-F)** Viability and islet insulin secretion function were preserved after roGFP overexpression for both mouse (**C,D**) and human (**E,F**) islets. All data are presented as mean ± SD ($n \geq 3$; ns: $p>0.05$).

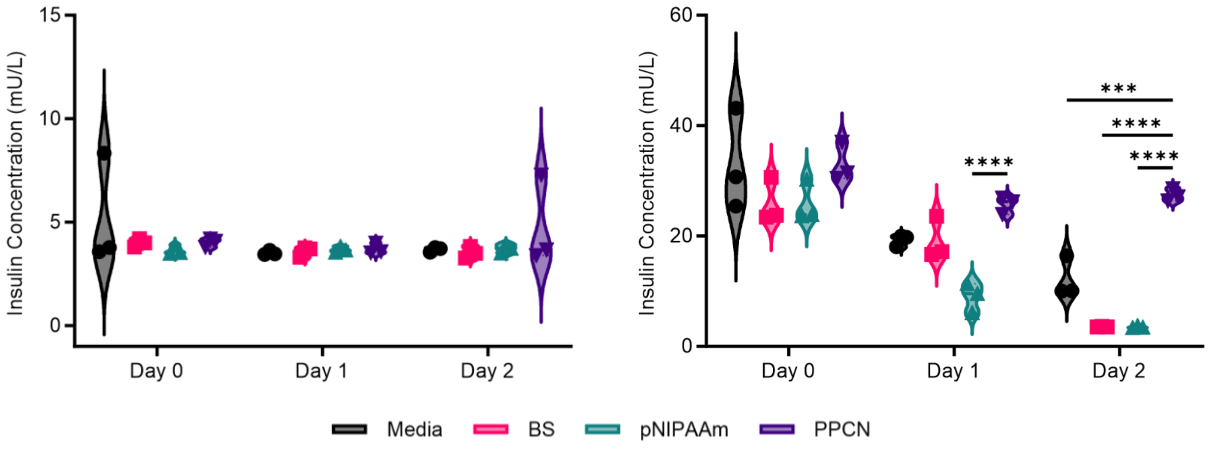

Fig. S4. Insulin concentrations during glucose-stimulated insulin secretion (GSIS) assay for mouse islets.

Secreted insulin concentrations during GSIS assay in response to low glucose (2.8 mM; left) and high glucose (28 mM) environments. Insulin concentration was measured via species-specific ELISA. All data are presented as mean ± SD with *** p<0.001, **** p<0.0001 relative to PPCN. Statistical significance was determined by two-way ANOVA with Tukey's multiple comparisons test. (n = 3-5).

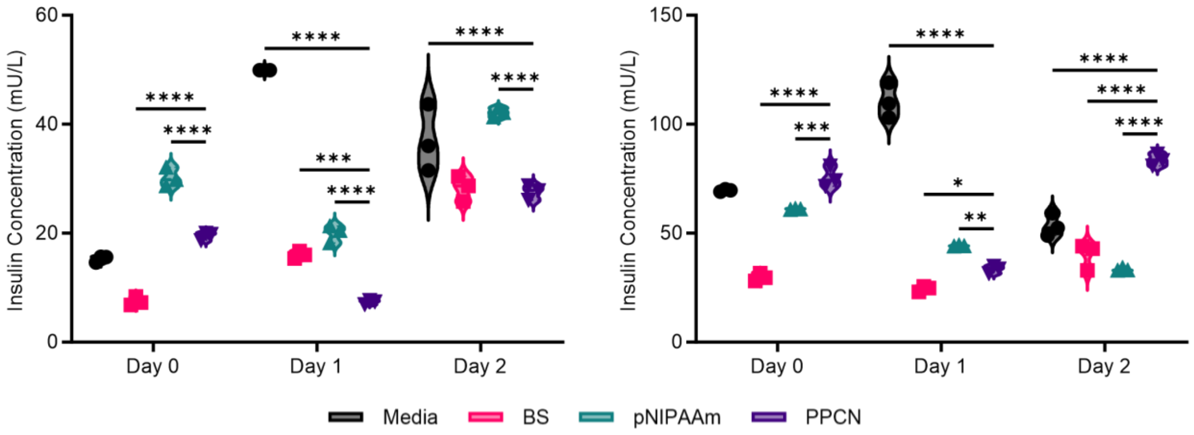

Fig. S5. Insulin concentrations during glucose-stimulated insulin secretion (GSIS) assay for human islets.

Secreted insulin concentrations during GSIS assay in response to low glucose (2.8 mM; left) and high glucose (28 mM) environments. Insulin concentration was measured via species-specific ELISA. All data are presented as mean ± SD with * p<0.05, ** p<0.01, *** p<0.001, **** p<0.0001 **** p<0.0001 relative to PPCN. Statistical significance was determined by two-way ANOVA with Tukey's multiple comparisons test. (n = 3-5).

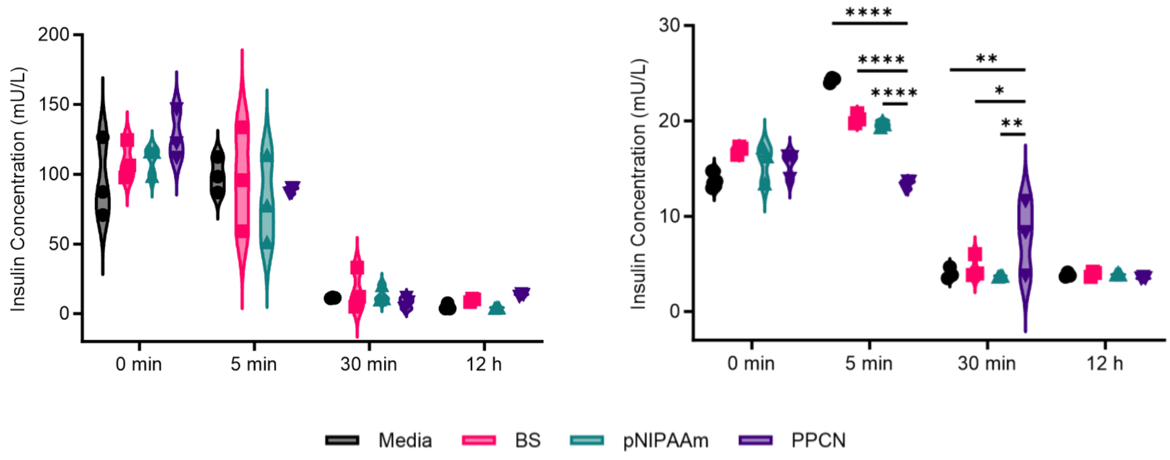

Fig. S6. Insulin concentrations during glucose-stimulated insulin secretion (GSIS) assay for mouse islets treated with H_2O_2. Secreted insulin concentrations during GSIS assay in response to low glucose (2.8 mM; left) and high glucose (28 mM) environments. Insulin concentration was measured via species-specific ELISA. All data are presented as mean ± SD with *p<0.05, **p<0.01, **** p<0.0001 relative to PPCN. Statistical significance was determined by two-way ANOVA with Tukey's multiple comparisons test. (n = 3-5).

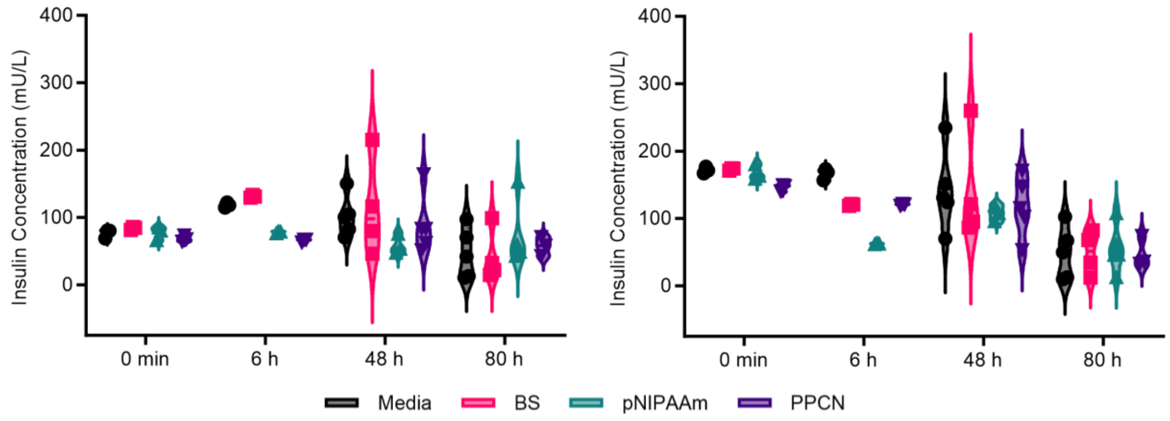

Fig. S7. Insulin concentrations during glucose-stimulated insulin secretion (GSIS) assay for human islets treated with H_2O_2. Secreted insulin concentrations during GSIS assay in response to low glucose (2.8 mM; left) and high glucose (28 mM) environments. Insulin concentration was measured via species-specific ELISA. All data are presented as mean ± SD. Two-way ANOVA with Tukey's multiple comparisons test was performed. (n = 3-5).

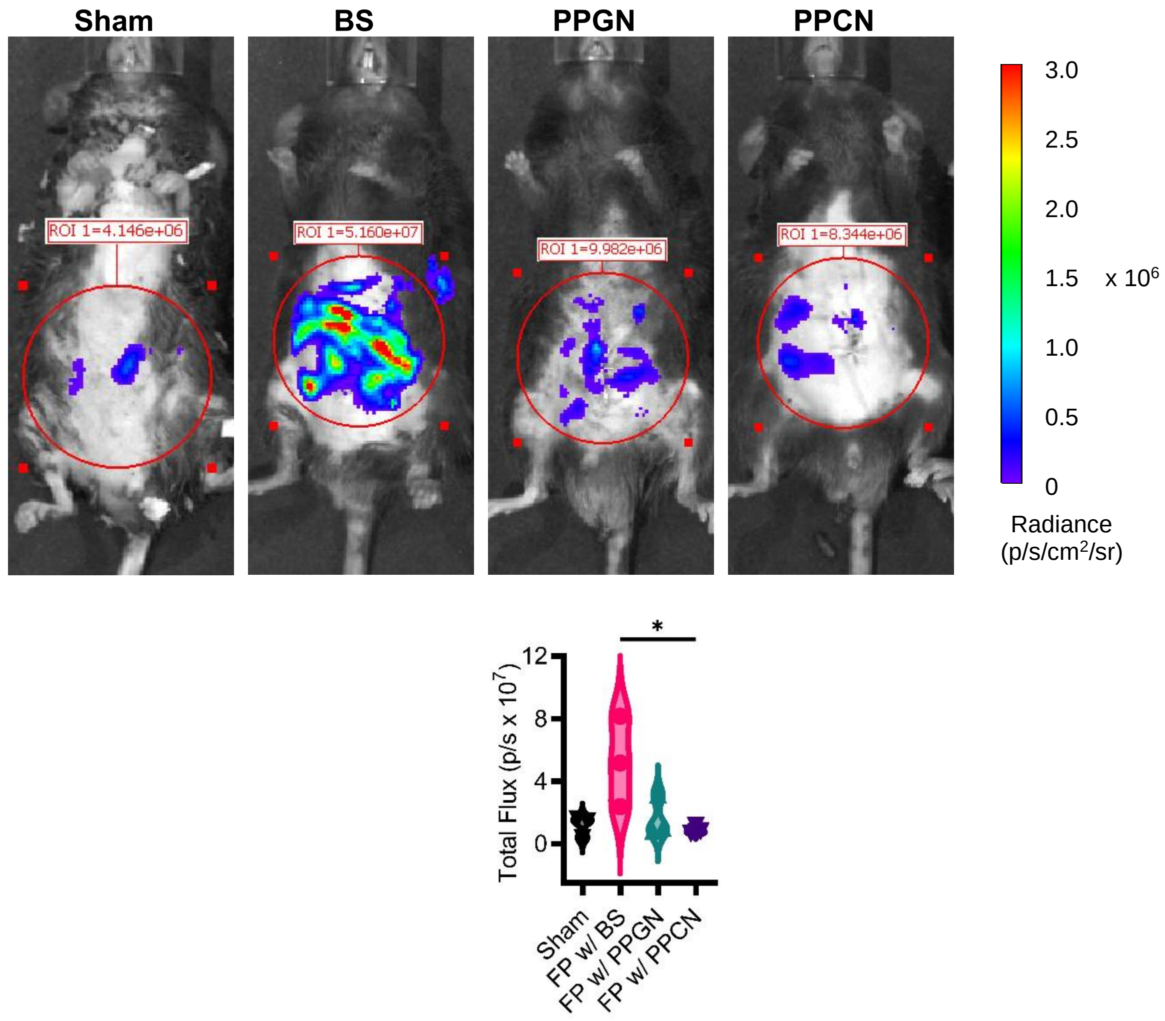

Fig. S8. Assessment of reactive oxygen species (ROS) following islet transplantation including fat pad transplantation with PPGN. IVIS images (**top**) and quantification (**botto,**) of reactive oxygen and nitrogen species *in vivo* 24 hours post-transplantation as measured via IVIS by the total flux of L-012 activity. All data are presented as mean ± SD with $*p<0.05$. Statistical significance was determined by one-way ANOVA with Tukey's multiple comparisons test. ($n = 3$).

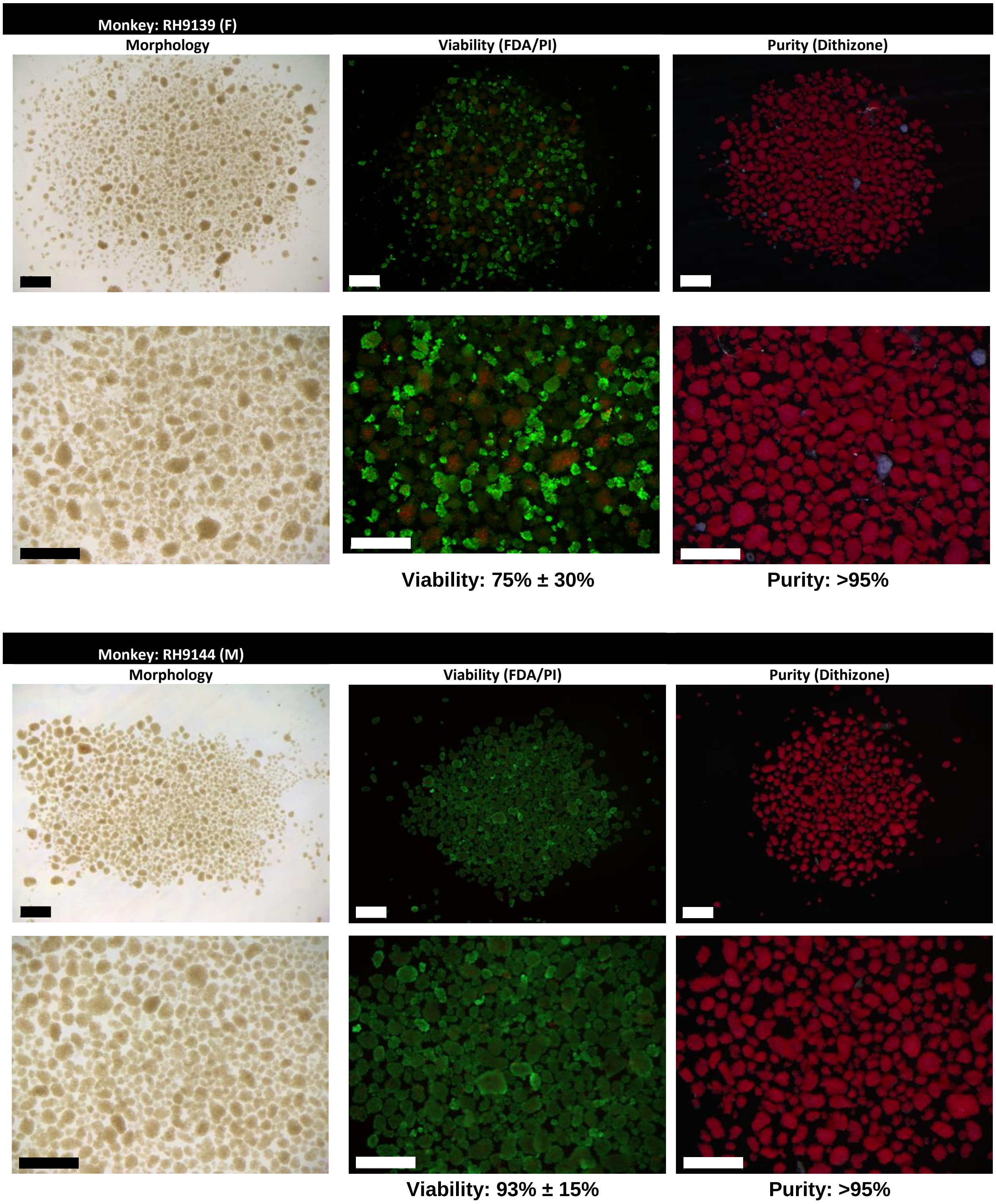

Fig. S9. Isolated nonhuman primate (NHP) islets for TP-IAT to the omentum with PPCN study. (n = 2; 1 female (F), 1 male (M)) Purified islets after overnight culture. Left: light microscope images for assessment of islet morphology, center: fluorescein diacetate (FDA; green/live) and propidium iodine (PI; red/dead) staining for assessment of islet viability; right: dithizone staining for assessment of islet purity. (scale bars: 500 µm).

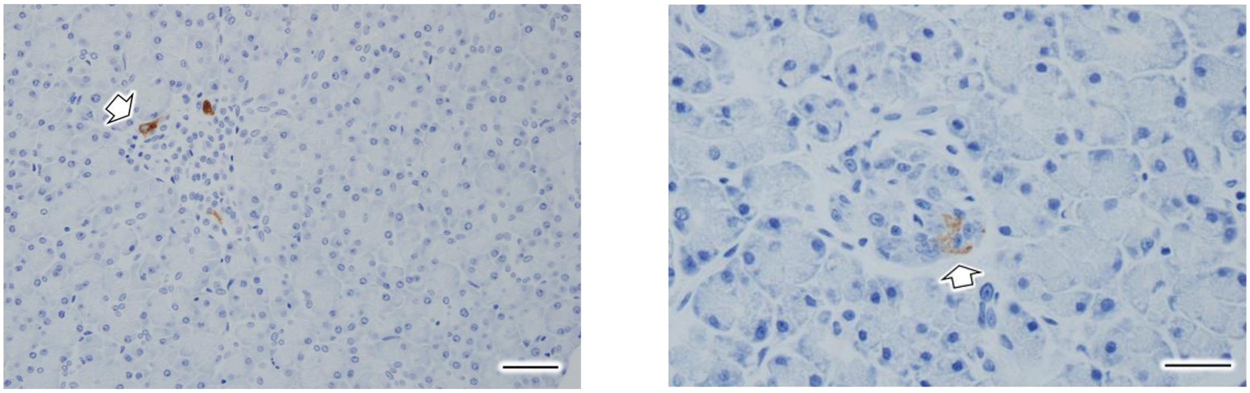

Fig. S10. Confirmation of beta cell depletion in the pancreas of nonhuman primates (NHPs). (n = 2; 1 female (F), 1 male (M)) Anti-insulin immunohistochemistry of pancreas tissue following necropsy. Left: RH9139 (F); Right: RH9144 (M). Arrows indicate rare, scattered insulin-producing (beta) cells with a granular cytoplasmic staining pattern. (scale bar: 50 µm).

Table S1. Human islet characteristics by isolation. The table format was modified from N.J. Hart, A.C. Powers, *Diabetologia* (2019).

	2017-03-19	2017-05-27	2018-05-12	2020-03-11
**Unique identifier**	ACEL461	AEEK023	AFEH099	AHCH342
**Age (years)**	30	46	45	42
**Sex (M/F)**	F	F	M	M
**BMI (kg/m²)**	No data	No data	25.90	27.97
**Ethnicity**	No data	No data	Hispanic	White
**HbA1c (%)**	5.6	No data	5.1	4.9
**Cause of death**	No data	No data	Anoxia	Head Trama
**Diabetes? (Y/N)**	N	No data	N	N
**Pancreas origin/source**	Gift of Hope	Gift of Hope	OneLegacy	Donor Network of Arizona
**Warm ischemia time (h)**	No data	No data	No data	No data
**Cold ischemia time (h)**	3.02	8.31	8.53	11.77
**Islet handling and use**	For research			
**Isolation center**	Northwestern University Transplant Surgery Human Islet Center		Prodo Labs	
**Estimated purity (%)**	87	90	90	90
**Estimated viability (%)**	95	95	95	95
**Total culture time (h)**	38	No data in record<br>Estimate > 30	72	120
**Functional measurement**	GSIS<br>Average Index 2.11	GSIS<br>Average Index 2.11	GSIS<br>Glucose Concentration (nM)/ Average Insulin Secretion (ng/ng/h)<br>3/0.076<br>12/0.098<br>28/0.126<br>28+IBMX/0.246<br>3/0.045	GSIS<br>Glucose Concentration (nM)/ Average Insulin Secretion (ng/ng/h)<br>3/0.031<br>12/0.178<br>28/0.199<br>28+IBMX/0.280<br>3/0.019
**Description of purification and quantification**	Purification: COBE 2991 Procedure<br><br>Quantification:<br>High purity: 85,577 (87%)<br>Middle purity 44,357 (45%)<br>Total: 129,934	Purification: COBE 2991 Procedure<br><br>Quantification:<br>High purity: 385,781 (90%)	No data	No data
**Additional note**	Because the total IEQ of islets was not sufficient for clinical patient transplantation, the islets were converted to research. The islets given for research were high purity.	This isolation was for BLA tests and research only. The donor info and isolation records were not given in full.	None	None

Table S2. Complete blood counts (CBCs) for nonhuman primates (NHPs) over the course of the PPCN biocompatibility study.

(n = 4; 2 female (F), 2 male (M)); WBC = white blood cells; RBC = red blood cells; HGB = hemoglobin; HCT = hematocrit; MCV = mean corpuscular volume; MCH = mean corpuscular hemoglobin; MCHC = mean corpuscular hemoglobin concentration; PLT = platelets; MPV = mean platelet volume; RDW = red cell distribution width; NEUT = neutrophils; LYMPH = lymphocytes; MONO = monocytes; EOS = eosinophils; BASO = basophils; LUC = large unstained cells; RETIC =reticulocytes; MACRO = macrocytosis; HC-VAR = hemoglobin concentration variance; PLATCLMP = platelet clumps; HYPER = hyperchromia.

**Monkey:**	**RH9138 (F)**								
**Condition:**	Day of Tx							Day of Retrieval	**Units**
**Date:**	5/18/2020	5/26/2020	6/1/2020	6/8/2020	6/15/2020	7/13/2020	8/10/2020	8/18/2020	
**Day:**	0	8	14	21	28	56	84	92	
**WBC**	7.8	10.17	8.29	7.22	16.19	7.84	6.76	7.39	x10^3/µL
**RBC**	4.73	4.81	4.91	4.7	5.13	5.26	4.87	4.41	x10^6/µL
**HGB**	11.2	11.3	11.3	11.1	12.1	12.3	11.6	10.4	g/dL
**HCT**	34.8	34.7	36.3	34.4	39.4	37.2	35.6	32.5	%
**MCV**	73.6	72.1	73.9	73.1	76.8	70.6	73.1	73.8	fL
**MCH**	23.6	23.6	23.0	23.7	23.6	23.4	23.8	23.7	pg
**MCHC**	32.1	32.7	31.1	32.4	30.8	33.2	32.6	32.1	g/dL
**PLT**	356	353	353	352	403	277	327	257	x10^3/µL
**MPV**	7.5	7.9	7	6.9	7.4	6.8	7.2	6.9	fL
**RDW**	13.1	12.8	13.1	14.4	12.5	12.6	13.9	14	%
**%NEUT**	38.6	44.8	34.6	46.7	62.6	52.4	42.2	50.3	%
**%LYMPH**	57.7	49.3	57.2	48.2	34.3	43.4	53.5	46.1	%
**%MONO**	1.1	2.0	1.8	2.0	1.5	1.3	2.1	1.6	%
**%EOS**	1.90	3.10	5.40	2.40	0.80	2.30	1.30	1.20	%
**%BASO**	0.2	0.2	0.3	0.1	0.3	0.2	0.2	0.2	%
**%LUC**	0.5	0.5	0.7	0.6	0.4	0.4	0.7	0.6	%
**#NEUT**	3.01	4.56	2.86	3.37	10.14	4.1	2.85	3.72	x10^3/µL
**#LYMPH**	4.50	5.02	4.74	3.48	5.56	3.41	3.62	3.41	x10^3/µL
**#MONO**	0.09	0.21	0.15	0.14	0.25	0.10	0.14	0.12	x10^3/µL
**#EOS**	0.15	0.31	0.45	0.18	0.14	0.18	0.09	0.09	x10^3/µL
**#BASO**	0.02	0.02	0.02	0.01	0.04	0.01	0.02	0.02	x10^3/µL
**#LUC**	0.04	0.05	0.06	0.04	0.06	0.03	0.05	0.04	x10^3/µL
**%RETIC**	2.21	2.18	2.13	2.12	2.07	1.29	2.15	2.81	%
**#RETIC**	104.6	104.9	104.5	99.5	106.2	68	104.8	123.9	x10^9/µL
**MACRO**				+	+				
**HC-VAR**				+					
**HYPER**				+		+++	+	+	

Table S2 (Continued). CBCs for NHPs over the course of the PPCN biocompatibility study. (n = 4; 2 female (F), 2 male (M)); WBC = white blood cells; RBC = red blood cells; HGB = hemoglobin; HCT = hematocrit; MCV = mean corpuscular volume; MCH = mean corpuscular hemoglobin; MCHC = mean corpuscular hemoglobin concentration; PLT = platelets; MPV = mean platelet volume; RDW = red cell distribution width; NEUT = neutrophils; LYMPH = lymphocytes; MONO = monocytes; EOS = eosinophils; BASO = basophils; LUC = large unstained cells; RETIC =reticulocytes; MACRO = macrocytosis; HC-VAR = hemoglobin concentration variance; PLATCLMP = platelet clumps; HYPER = hyperchromia.

Monkey:	RH9142 (F)								
Condition:	Day of Tx							Day of Retrieval	Units
Date:	5/18/2020	5/26/2020	6/1/2020	6/8/2020	6/15/2020	7/13/2020	8/10/2020	8/18/2020	
Day:	0	8	14	21	28	56	84	92	
WBC	6.82	14.97	11.6	8.79	8.67	11.73	7.55	5.88	x10^3/µL
RBC	4.52	4.72	4.69	4.44	4.37	4.62	4.45	4.12	x10^6/µL
HGB	10.8	11.3	11.1	10.5	10.3	11.2	10.9	9.9	g/dL
HCT	33.6	35.1	35.5	32.4	33.7	33.7	33.6	31.3	%
MCV	74.2	74.3	75.6	73.0	77.1	72.9	75.5	75.8	fL
MCH	23.9	24.0	23.7	23.8	23.6	24.2	24.4	24.1	pg
MCHC	32.2	32.3	31.4	32.6	30.6	33.2	32.4	31.8	g/dL
PLT	271	349	345	310	302	308	341	241	x10^3/µL
MPV	7.3	7.2	6.2	6.2	6.4	6	6.1	6.2	fL
RDW	13	12.7	13.2	13.1	13	12.8	13.4	13.6	%
%NEUT	59.2	52.9	29.8	38.0	40.2	53.5	42.7	67.2	%
%LYMPH	38.0	40.9	61.9	57.3	54.6	41.8	51.5	29.9	%
%MONO	1.8	4.4	3.6	2.3	3.2	2.9	2.9	1.7	%
%EOS	0.70	1.20	4.10	1.70	1.40	0.80	2.00	0.80	%
%BASO	0.1	0.3	0.3	0.2	0.1	0.2	0.2	0.1	%
%LUC	0.2	0.4	0.4	0.5	0.5	0.8	0.7	0.3	%
#NEUT	4.04	7.91	3.45	3.34	3.49	6.27	3.22	3.95	x10^3/µL
#LYMPH	2.59	6.12	7.18	5.04	4.73	4.91	3.89	1.76	x10^3/µL
#MONO	0.12	0.66	0.41	0.20	0.27	0.34	0.22	0.10	x10^3/µL
#EOS	0.05	0.17	0.48	0.15	0.13	0.1	0.15	0.05	x10^3/µL
#BASO	0	0.04	0.04	0.02	0.01	0.02	0.02	0	x10^3/µL
#LUC	0.01	0.07	0.04	0.05	0.04	0.09	0.05	0.02	x10^3/µL
%RETIC	1.1	1.31	1.7	2.1	1.64	1.2	1.33	1.8	%
#RETIC	49.7	62	80	93.1	71.8	55.4	58.9	74.4	x10^9/µL
PLTCLMP									
HYPER				+		+	+		
MACRO					+		+	+	

Table S2 (Continued). CBCs for NHPs over the course of the PPCN biocompatibility study. (n = 4; 2 female (F), 2 male (M)); WBC = white blood cells; RBC = red blood cells; HGB = hemoglobin; HCT = hematocrit; MCV = mean corpuscular volume; MCH = mean corpuscular hemoglobin; MCHC = mean corpuscular hemoglobin concentration; PLT = platelets; MPV = mean platelet volume; RDW = red cell distribution width; NEUT = neutrophils; LYMPH = lymphocytes; MONO = monocytes; EOS = eosinophils; BASO = basophils; LUC = large unstained cells; RETIC =reticulocytes; MACRO = macrocytosis; HC-VAR = hemoglobin concentration variance; PLATCLMP = platelet clumps; HYPER = hyperchromia.

Monkey:	RH9145 (M)								
Condition:	Day of Tx							Day of Retrieval	Units
Date:	5/18/2020	5/26/2020	6/1/2020	6/8/2020	6/15/2020	7/13/2020	8/10/2020	8/18/2020	
Day:	0	8	14	21	28	56	84	92	
WBC	5.33	12.94	9.03	8.71	9.98	8.19	7.45	6.76	x10^3/µL
RBC	4.85	4.91	4.78	4.89	5.01	5.1	4.77	4.93	x10^6/µL
HGB	11.4	11.2	11.0	11.0	11.1	11.6	11.1	11.4	g/dL
HCT	34.9	35.4	34.4	34.4	36.6	35.2	34.4	35.7	%
MCV	71.9	72.1	72.0	70.3	73.1	69.1	72.2	72.4	fL
MCH	23.6	22.9	22..9	22.6	22.2	22.7	23.2	23.1	pg
MCHC	32.8	31.8	31.8	32.1	30.4	32.9	32.2	31.9	g/dL
PLT	322	389	352	369	384	347	394	315	x10^3/µL
MPV	6.6	6.8	5.6	5.6	5.8	5.6	5.7	5.9	fL
RDW	12.5	12.5	12.7	12.5	12.4	13.1	13.2	13.4	%
%NEUT	24.8	54.4	32.2	23.9	18.6	24.3	18.3	27.8	%
%LYMPH	70.8	42.0	62.2	68.6	75.2	70.6	77.7	65.8	%
%MONO	2.3	1.9	2.5	1.6	1.6	1.3	1.2	1.2	%
%EOS	1.20	1.00	2.00	4.70	3.40	2.50	1.50	4.10	%
%BASO	0.2	0.3	0.2	0.3	0.4	0.3	0.3	0.1	%
%LUC	0.7	0.4	0.8	0.9	0.8	1	1.1	1	%
#NEUT	1.32	7.04	2.91	2.08	1.86	1.99	1.36	1.88	x10^3/µL
#LYMPH	3.77	5.44	5.62	5.98	7.51	5.79	5.79	4.45	x10^3/µL
#MONO	0.12	0.25	0.23	0.14	0.16	0.10	0.09	0.08	x10^3/µL
#EOS	0.07	0.13	0.18	0.41	0.34	0.2	0.11	0.27	x10^3/µL
#BASO	0.01	0.04	0.02	0.03	0.04	0.03	0.02	0.01	x10^3/µL
#LUC	0.04	0.06	0.07	0.08	0.08	0.08	0.08	0.07	x10^3/µL
%RETIC	1.22	0.95	0.95	1.2	1.23	1.09	1.17	1.34	%
#RETIC	59.1	46.4	45.4	58.7	61.7	55.6	55.7	66.2	x10^9/µL
PLTCLMP	+								
HYPER						++			

Table S2 (Continued). CBCs for NHPs over the course of the PPCN biocompatibility study. (n = 4; 2 female (F), 2 male (M)); WBC = white blood cells; RBC = red blood cells; HGB = hemoglobin; HCT = hematocrit; MCV = mean corpuscular volume; MCH = mean corpuscular hemoglobin; MCHC = mean corpuscular hemoglobin concentration; PLT = platelets; MPV = mean platelet volume; RDW = red cell distribution width; NEUT = neutrophils; LYMPH = lymphocytes; MONO = monocytes; EOS = eosinophils; BASO = basophils; LUC = large unstained cells; RETIC =reticulocytes; MACRO = macrocytosis; HC-VAR = hemoglobin concentration variance; PLATCLMP = platelet clumps; HYPER = hyperchromia.

**Monkey:**	**RH9148 (M)**								
**Condition:**	Day of Tx							Day of Retrieval	**Units**
**Date:**	5/18/2020	5/26/2020	6/1/2020	6/8/2020	6/15/2020	7/13/2020	8/10/2020	8/18/2020	
**Day:**	0	8	14	21	28	56	84	92	
**WBC**	8.78	13.47	11.05	9.54	16.08	10.35	11.69	10.09	x10^3/µL
**RBC**	5.47	5.49	5.4	5.41	5.3	5.45	5.59	5.49	x10^6/µL
**HGB**	12.9	12.7	12.6	12.5	12.1	12.7	13.1	12.6	g/dL
**HCT**	40.3	40.5	40.2	39.2	40.6	38.7	41.4	40.8	%
**MCV**	73.6	73.8	74.4	72.5	76.6	70.9	74.0	74.3	fL
**MCH**	23.6	23.2	23.3	23.1	22.9	23.4	23.5	22.9	pg
**MCHC**	32	31.4	31.3	31.9	29.9	32.9	31.8	30.9	g/dL
**PLT**	357	387	347	349	382	282	332	342	x10^3/µL
**MPV**	7.5	7.7	7.3	6.8	7.2	6.7	7.3	7	fL
**RDW**	12.6	12.3	12.7	12.8	12.6	13.2	13.4	13.5	%
**%NEUT**	22.0	35.1	19.0	17.5	35.7	18.6	16.6	68.6	%
**%LYMPH**	75.4	62.0	77.4	79.7	60.5	79.1	80.8	29.7	%
**%MONO**	1.2	1.4	1.6	0.9	2.0	0.8	0.8	0.8	%
**%EOS**	0.40	0.70	0.90	1.00	0.80	0.30	0.40	0.60	%
**%BASO**	0.3	0.3	0.4	0.3	0.3	0.3	0.4	0.2	%
**%LUC**	0.6	0.5	0.7	0.6	0.6	0.8	1.1	0.2	%
**#NEUT**	1.93	4.72	2.1	1.67	5.74	1.93	1.94	6.91	x10^3/µL
**#LYMPH**	6.63	8.35	8.55	7.60	9.74	8.19	9.44	3.00	x10^3/µL
**#MONO**	0.11	0.19	0.17	0.09	0.33	0.09	0.09	0.08	x10^3/µL
**#EOS**	0.04	0.09	0.1	0.1	0.13	0.03	0.05	0.06	x10^3/µL
**#BASO**	0.02	0.05	0.04	0.03	0.06	0.03	0.04	0.02	x10^3/µL
**#LUC**	0.05	0.06	0.08	0.05	0.09	0.09	0.13	0.02	x10^3/µL
**%RETIC**	1.3	0.83	1.54	1.57	1.5	1.09	1.03	1.07	%
**#RETIC**	71.1	45.8	83.1	85.2	79.7	59.3	57.6	58.9	x10^9/µL
**PLTCLMP**					+				
**HYPER**						++			

Table S3. Blood chemistry panel for nonhuman primates (NHPs) over the course of the PPCN biocompatibility study.

(n = 4; 2 female (F), 2 male (M)); ALT = alanine transaminase; AST = aspartate aminotransferase; BUN = blood urea nitrogen; GGT = gamma-glutamyl transferase; I = inorganic; LIP = lipemia; ICH = icterus; HEM = hemolysis.

**Monkey:**	**RH9138 (F)**								
**Condition:**	Day of Tx							Day of Retrieval	**Units**
**Date:**	5/18/2020	5/26/2020	6/1/2020	6/8/2020	6/15/2020	7/13/2020	8/10/2020	8/18/2020	
**Day:**	0	8	14	21	28	56	84	92	
Albumin	4.008	3.605	3.692	4.149	4.343	4.613	4.149	3.733	g/dL
Alkaline Phosphatase	251	274	274	297	318	236	219	233	U/L
ALT	29	23	20	30	26	29	24	24	U/L
AST	28	28	26	25	36	30	30	35	U/L
Urea Nitrogen (BUN)	21	19	21	21	23	20	17	16	mg/dL
Calcium	8.85	8.91	8.43	9.09	9.25	9.21	8.81	8.39	mg/dL
Creatinine	0.5402	0.3978	0.4171	0.3939	0.6184	0.6079	0.5911	0.4869	mg/dL
GGT	63	68	65	65	67	59	48	45	U/L
Glucose	68	60	74	77	76	74	68	60	mg/dL
I. Phosphorous	6.4	5.9	5.6	5.2	6	4.7	4	3.9	mg/dL
Total Bilirubin	0.14	0.14	0.18	0.2	0.16	0.2	0.19	0.27	mg/dL
Total Protein	6.072	5.573	5.412	6.489	6.585	7.012	6.2	5.545	g/dL
Sodium	144	143	142	144	145	145	144	145	mmol/L
Potassium	3.3	3.2	3.2	3.6	3.3	3.3	3.4	3.3	mmol/L
Chloride	104	105	104	108	107	104	106	108	mmol/L
LIP	N	N	N	N	N	N	N	N	
ICH	N	N	N	N	N	N	N	N	
HEM	N	N	N	N	N	N	N	N	
Globulin	2.064	1.968	1.72	2.34	2.242	2.399	2.051	1.812	g/dL
Alb/Glob Ratio	2	2	2	2	2	2	2	2	

Table S3 (Continued). Blood chemistry panel for NHPs over the course of the PPCN biocompatibility study.

(n = 4; 2 female (F), 2 male (M)); ALT = alanine transaminase; AST = aspartate aminotransferase; BUN = blood urea nitrogen; GGT = gamma-glutamyl transferase; I = inorganic; LIP = lipemia; ICH = icterus; HEM = hemolysis.

**Monkey:**	**RH9142 (F)**								
**Condition:**	Day of Tx							Day of Retrieval	**Units**
**Date:**	5/18/2020	5/26/2020	6/1/2020	6/8/2020	6/15/2020	7/13/2020	8/10/2020	8/18/2020	
**Day:**	0	8	14	21	28	56	84	92	
Albumin	4.04	3.98	4.148	3.987	4.007	4.449	4.063	3.773	g/dL
Alkaline Phosphatase	364	391	377	359	306	248	213	249	U/L
ALT	19	20	25	28	20	24	39	25	U/L
AST	22	22	23	24	21	21	28	25	U/L
Urea Nitrogen (BUN)	18	15	18	14	17	20	16	14	mg/dL
Calcium	8.38	8.78	8.82	8.84	9.24	8.97	8.83	8.06	mg/dL
Creatinine	0.5171	0.5155	0.4403	0.4017	0.4636	0.4523	0.4421	0.4481	mg/dL
GGT	53	53	54	49	47	46	46	40	U/L
Glucose	61	64	79	65	75	58	55	63	mg/dL
I. Phosphorous	5.7	5.5	5.9	6.2	4.6	5.4	5	4.6	mg/dL
Total Bilirubin	0.24	0.17	0.18	0.15	0.15	0.2	0.15	0.3	mg/dL
Total Protein	5.81	6.054	5.963	5.915	5.889	6.745	6.17	5.553	g/dL
Sodium	145	143	142	144	144	145	145	145	mmol/L
Potassium	3.2	3	3.2	3.3	3.4	3.3	3.4	3.1	mmol/L
Chloride	107	104	104	106	108	106	108	111	mmol/L
LIP	N	N	N	N	N	N	N	N	
ICH	N	N	N	N	N	N	N	N	
HEM	N	N	N	N	N	N	N	N	
Globulin	1.77	2.074	1.815	1.928	1.862	2.296	2.107	1.78	g/dL
Alb/Glob Ratio	2	2	2	2	2	2	2	2	

Table S3 (Continued). Blood chemistry panel for NHPs over the course of the PPCN biocompatibility study.

(n = 4; 2 female (F), 2 male (M)); ALT = alanine transaminase; AST = aspartate aminotransferase; BUN = blood urea nitrogen; GGT = gamma-glutamyl transferase; I = inorganic; LIP = lipemia; ICH = icterus; HEM = hemolysis.

Monkey:	RH9145 (M)								
Condition:	Day of Tx							Day of Retrieval	Units
Date:	5/18/2020	5/26/2020	6/1/2020	6/8/2020	6/15/2020	7/13/2020	8/10/2020	8/18/2020	
Day:	0	8	14	21	28	56	84	92	
Albumin	4.106	4.142	4.127	4.211	4.33	4.3	4.082	3.965	g/dL
Alkaline Phosphatase	442	403	366	428	428	423	356	419	U/L
ALT	31	31	36	35	40	36	35	38	U/L
AST	31	30	25	29	34	28	28	26	U/L
Urea Nitrogen (BUN)	20	21	26	21	21	21	20	14	mg/dL
Calcium	9.17	9.35	9.32	9.19	9.81	9.11	8.97	8.95	mg/dL
Creatinine	0.5171	0.5939	0.4557	0.4485	0.5487	0.4367	0.4794	0.4481	mg/dL
GGT	69	70	65	67	70	77	68	66	U/L
Glucose	56	91	83	77	80	72	95	61	mg/dL
I. Phosphorous	6.3	5.3	5.1	5.6	5.2	5.8	5.7	6	mg/dL
Total Bilirubin	0.22	0.14	0.16	0.18	0.19	0.29	0.2	0.22	mg/dL
Total Protein	6.065	6.267	6.222	6.818	6.712	6.482	6.165	5.98	g/dL
Sodium	145	144	141	144	144	144	143	143	mmol/L
Potassium	3.5	3.3	3.2	3.3	3.3	3.3	3.7	3.3	mmol/L
Chloride	104	102	102	105	103	105	106	105	mmol/L
LIP	N	N	N	N	N	N	N	N	
ICH	N	N	N	N	N	N	N	N	
HEM	N	N	N	N	N	N	N	N	
Globulin	2.049	2.125	2.095	2.607	2.382	2.182	2.083	2.015	g/dL
Alb/Glob Ratio	2	2	2	2	2	2	2	2	

Table S3 (Continued). Blood chemistry panel for NHPs over the course of the PPCN biocompatibility study.

(n = 4; 2 female (F), 2 male (M)); ALT = alanine transaminase; AST = aspartate aminotransferase; BUN = blood urea nitrogen; GGT = gamma-glutamyl transferase; I = inorganic; LIP = lipemia; ICH = icterus; HEM = hemolysis.

**Monkey:**	**RH9148 (M)**								
**Condition:**	Day of Tx							Day of Retrieval	
**Date:**	5/18/2020	5/26/2020	6/1/2020	6/8/2020	6/15/2020	7/13/2020	8/10/2020	8/18/2020	
**Day:**	0	8	14	21	28	56	84	92	**Units**
Albumin	3.833	3.8	4.041	4.015	3.918	3.952	3.876	3.886	g/dL
Alkaline Phosphatase	723	391	671	759	664	681	598	704	U/L
ALT	30	35	27	34	29	28	30	38	U/L
AST	35	26	39	34	34	31	34	49	U/L
Urea Nitrogen (BUN)	25	17	28	25	31	25	26	24	mg/dL
Calcium	9.21	9.27	9.16	9.15	9.16	9.01	8.89	9.11	mg/dL
Creatinine	0.5017	0.539	0.4635	0.3939	0.5487	0.4289	0.5389	0.6108	mg/dL
GGT	63	74	60	62	58	62	60	59	U/L
Glucose	61	61	65	60	63	67	65	78	mg/dL
I. Phosphorous	7.3	6	6.9	6.4	6.2	6.5	6.4	6.8	mg/dL
Total Bilirubin	0.25	0.16	0.2	0.26	0.18	0.27	0.18	0.39	mg/dL
Total Protein	5.8	6.33	5.878	6.157	5.808	5.829	5.829	5.749	g/dL
Sodium	143	145	142	144	143	144	144	146	mmol/L
Potassium	3.4	3.7	3.4	3.4	3.4	3.5	3.4	3.4	mmol/L
Chloride	103	106	103	106	103	106	105	105	mmol/L
LIP	N	N	N	N	N	N	N	N	
ICH	N	N	N	N	N	N	N	N	
HEM	N	N	N	N	N	N	N	N	
Globulin	1.967	2.53	1.837	2.142	1.89	1.877	1.953	1.863	g/dL
Alb/Glob Ratio	2	2	2	2	2	2	2	2	

Table S4. Urinalysis for nonhuman primates (NHPs) over the course of the PPCN biocompatibility study.
(n = 4; 2 female (F), 2 male (M)); RBC = red blood cells; WBC = white blood cells.

	Monkey:	RH9138 (F)					
	Condition:	Day of Tx					
	Date:	5/18/2020	5/26/2020	6/1/2020	6/8/2020	6/15/2020	7/13/2020
	Day:	0	8	14	21	28	56
Physical	Appearance	Clear	Clear	Clear	Hazy	Clear	Clear
	Specific Gravity	1.025	1.005	1.021	1.025	1.026	1.02
	Color	Yellow	Light Yellow	Yellow	Light Yellow	Yellow	Yellow
Dipstick Evaluation	Leukocytes	Negative	Negative	Negative	Negative	Negative	Negative
	Nitrite	Negative	Negative	Negative	Negative	Negative	Negative
	pH	7	7	6	6	5	8
	Protein	Trace	Negative	Trace	Trace	Trace	Trace
	Glucose	Normal	Normal	Normal	Normal	Normal	Normal
	Ketones	Negative	Negative	Negative	Negative	Negative	Negative
	Urobilinogen	Normal	Normal	Normal	Normal	Normal	Normal
	Bilirubin	Negative	Negative	Negative	Negative	Negative	Negative
	Blood	Negative	Trace	Negative	Trace	Trace	Negative
Sediment Evaluation	Cast Type	-	-	-	-	None seen	None seen
	Cast Average/10x Field	0	0	0	0	-	-
	Cast Type	-	-	-	-	-	-
	Cast Average/10x Field	-	-	-	0	-	-
	RBC's Average/45x Field	0	0-2	0	3-6	0-1	0-1
	WBC's Average/45x Field	0-1	0	0	Rare	0	0
	Epi cells Type	Squamous	Squamous	Squamous	Squamous	None seen	Squamous
	Epi cells Average/45x Field	0-1	0-1	0-2	3-large clumps	-	0-2
	Epi cells Type	-	Transitional	-	Transitional	-	-
	Epi cells Average/10x Field	-	2.0-3.0	-	0-large clumps	-	-
	Crysal Type	-	-	-	-	-	-
	Crystal Severity/45x Field	0	0	0	0	0	0
	Crystal Type	-	-	-	-	-	-
	Crystal Severity/45x Field	-	-	0	0	-	-
	Bacteria Type	-	-	-	Cocci-motile	-	-
	Bacteria Severity 45x Field	0	0	1+	1+	0	0
	Sperm Severity 45x Field	-	0	0	0	0	0
	Mucus Severity 45x Field	-	0	0	0	0	0
	Yeast Severity 45x Field	-	0	0	0	0	0

Table S4 (Continued). Urinalysis for NHPs over the course of the PPCN biocompatibility study. (n = 4; 2 female (F), 2 male (M)); RBC = red blood cells; WBC = white blood cells.

	**Monkey:**	**RH9138 (F)**	
	**Condition:**		Day of Retrieval
	**Date:**	8/10/2020	8/18/2020
	**Day:**	84	92
Physical	**Appearance**	Clear	Clear
	**Specific Gravity**	1.022	1.014
	**Color**	Yellow	Yellow
Dipstick Evaluation	**Leukocytes**	Negative	Negative
	**Nitrite**	Negative	Negative
	**pH**	8	7
	**Protein**	Trace	+ (30)
	**Glucose**	Normal	Normal
	**Ketones**	Negative	Negative
	**Urobilinogen**	Normal	Normal
	**Bilirubin**	Negative	Negative
	**Blood**	Negative	Trace
Sediment Evaluation	**Cast Type**	None seen	-
	**Cast Average/10x Field**	-	0
	**Cast Type**	-	-
	**Cast Average/10x Field**	-	0
	**RBC's Average/45x Field**	0	1-6
	**WBC's Average/45x Field**	0	0
	**Epi cells Type**	Transitional	Squamous
	**Epi cells Average/45x Field**	0-1	0-3
	**Epi cells Type**	-	-
	**Epi cells Average/10x Field**	-	0
	**Crysal Type**	None seen	Amorphous debri
	**Crystal Severity/45x Field**	-	2+
	**Crystal Type**	-	-
	**Crystal Severity/45x Field**	-	0
	**Bacteria Type**	-	Cocci
	**Bacteria Severity 45x Field**	2+	0 - Rare
	**Sperm Severity 45x Field**	0	0
	**Mucus Severity 45x Field**	0	0
	**Yeast Severity 45x Field**	0	0

Table S4 (Continued). Urinalysis for NHPs over the course of the PPCN biocompatibility study. (n = 4; 2 female (F), 2 male (M)); RBC = red blood cells; WBC = white blood cells.

	Monkey:	RH9142 (F)				
	Condition:	Day of Tx				
	Date:	5/18/2020	5/26/2020	6/1/2020	6/8/2020	6/15/2020
	Day:	0	8	14	21	28
Physical	Appearance	Clear	Clear	Clear	Clear	Hazy
	Specific Gravity	1.016	1.01	1.005	1.004	1.008
	Color	Light Yellow	Yellow	Light Yellow	Pale Yellow	Light Yellow
Dipstick Evaluation	Leukocytes	Negative	Negative	Negative	Negative	Negative
	Nitrite	Negative	Negative	Negative	Positive	Negative
	pH	7	8	8	8	5
	Protein	Trace	Negative	Negative	Trace	Negative
	Glucose	Normal	Normal	Normal	Normal	Normal
	Ketones	Negative	Negative	Negative	Negative	Negative
	Urobilinogen	Normal	Normal	Normal	Normal	Normal
	Bilirubin	Negative	Negative	Negative	Negative	Negative
	Blood	Trace	Trace	250 Ery/µL	Trace	Trace
Sediment Evaluation	Cast Type	-	-	-	-	None seen
	Cast Average/10x Field	0	0	0	0	-
	Cast Type	-	-	-	-	-
	Cast Average/10x Field	-	-	-	0	-
	RBC's Average/45x Field	0-1	0	0	0-1	0
	WBC's Average/45x Field	0	0	0	0	0
	Epi cells Type	Squamous	Squamous	Squamous	Squamous	None seen
	Epi cells Average/45x Field	0-1	0-1	0-1	1-3	-
	Epi cells Type	-	-	Transitional	-	-
	Epi cells Average/10x Field	-	-	0-1	0	-
	Crysal Type	-	-	-	Amorphous	None seen
	Crystal Severity/45x Field	0	0	0	1+	-
	Crystal Type	-	-	-	-	-
	Crystal Severity/45x Field	-	-	-	0	-
	Bacteria Type	-	-	-	cocci-motile (clostridium)	-
	Bacteria Severity 45x Field	1+	0	1+	1+	0
	Sperm Severity 45x Field	0	0	-	0	0
	Mucus Severity 45x Field	0	0	-	0	0
	Yeast Severity 45x Field	0	0	-	0	0

Table S4 (Continued). Urinalysis for NHPs over the course of the PPCN biocompatibility study. (n = 4; 2 female (F), 2 male (M)); RBC = red blood cells; WBC = white blood cells.

	**Monkey:**	**RH9142 (F)**		
	**Condition:**			Day of Retrieval
	**Date:**	7/13/2020	8/10/2020	8/18/2020
	**Day:**	56	84	92
Physical	**Appearance**	Clear	Clear	Clear
	**Specific Gravity**	1.011	1.005	1.007
	**Color**	Light Yellow	Light Yellow	Light Yellow
Dipstick Evaluation	**Leukocytes**	Negative	Negative	Negative
	**Nitrite**	Negative	Negative	Negative
	**pH**	7	7	5
	**Protein**	Negative	Trace	Trace
	**Glucose**	Normal	Normal	Normal
	**Ketones**	Negative	Negative	Negative
	**Urobilinogen**	Normal	Normal	Normal
	**Bilirubin**	Negative	Negative	Negative
	**Blood**	Trace	Negative	Trace
Sediment Evaluation	**Cast Type**	None seen	None seen	-
	**Cast Average/10x Field**	-	-	0
	**Cast Type**	-	-	-
	**Cast Average/10x Field**	-	-	0
	**RBC's Average/45x Field**	1-2	0	0-2
	**WBC's Average/45x Field**	0	0	0
	**Epi cells Type**	Squamous	-	Squamous
	**Epi cells Average/45x Field**	0-1	0	0-2
	**Epi cells Type**	-	-	-
	**Epi cells Average/10x Field**	-	-	0
	**Crysal Type**	-	None seen	-
	**Crystal Severity/45x Field**	0	-	0
	**Crystal Type**	-	-	-
	**Crystal Severity/45x Field**	-	-	0
	**Bacteria Type**	-	-	Cocci-motile
	**Bacteria Severity 45x Field**	1+	1+	1+
	**Sperm Severity 45x Field**	0	0	0
	**Mucus Severity 45x Field**	0	0	0
	**Yeast Severity 45x Field**	0	0	0

Table S4 (Continued). Urinalysis for NHPs over the course of the PPCN biocompatibility study. (n = 4; 2 female (F), 2 male (M)); RBC = red blood cells; WBC = white blood cells.

	Monkey:	RH9145 (M)			
	Condition:	Day of Tx			
	Date:	5/18/2020	5/26/2020	6/1/2020	6/8/2020
	Day:	0	8	14	21
Physical	Appearance	Clear	Clear	Clear	Cloudy
	Specific Gravity	1.026	1.006	1.02	1.026
	Color	Yellow	Colorless	Light Yellow	Yellow
Dipstick Evaluation	Leukocytes	Negative	Negative	Negative	Negative
	Nitrite	Negative	Negative	Negative	Negative
	pH	7	8	8	9
	Protein	Trace	Negative	Trace	+ (30)
	Glucose	Normal	Normal	Normal	Normal
	Ketones	Negative	Negative	Negative	Negative
	Urobilinogen	Normal	Normal	Normal	Normal
	Bilirubin	Negative	Negative	Negative	Negative
	Blood	Negative	Negative	50	250 Ery/µL
Sediment Evaluation	Cast Type	-	-	-	-
	Cast Average/10x Field	0	0	0	0
	Cast Type	-	-	-	-
	Cast Average/10x Field	-	-	-	0
	RBC's Average/45x Field	0	0	0	>50
	WBC's Average/45x Field	0	0	0	0
	Epi cells Type	-	-	-	Transitional
	Epi cells Average/45x Field	0	-	0	0 -small clumps
	Epi cells Type	-	-	-	-
	Epi cells Average/10x Field	-	-	-	0
	Crysal Type	-	-	-	-
	Crystal Severity/45x Field	0	0	0	0
	Crystal Type	-	-	-	-
	Crystal Severity/45x Field	-	-	-	0
	Bacteria Type	-	-	-	Cocci-motile
	Bacteria Severity 45x Field	1+	0	1+	Rare
	Sperm Severity 45x Field	0	0	0	0
	Mucus Severity 45x Field	0	0	0	0
	Yeast Severity 45x Field	0	0	0	0

Table S4 (Continued). Urinalysis for NHPs over the course of the PPCN biocompatibility study. (n = 4; 2 female (F), 2 male (M)); RBC = red blood cells; WBC = white blood cells.

	Monkey:	RH9145 (M)			
	Condition:				Day of Retrieval
	Date:	6/15/2020	7/13/2020	8/10/2020	8/18/2020
	Day:	28	56	84	92
Physical	Appearance	Clear	Clear	-	Clear
	Specific Gravity	1.002	1.007	1.013	1.032
	Color	Colorless	Colorless	Yellow	Yellow
Dipstick Evaluation	Leukocytes	Negative	Negative	Negative	Negative
	Nitrite	Negative	Negative	Negative	Negative
	pH	7	8	8	6
	Protein	Trace	Trace	Trace	+ (30)
	Glucose	Normal	Normal	Normal	Normal
	Ketones	Negative	Negative	Negative	+ (Small)
	Urobilinogen	Normal	Normal	Normal	Normal
	Bilirubin	Negative	Negative	Negative	Negative
	Blood	250 Ery/µL	Trace	Trace	Negative
Sediment Evaluation	Cast Type	None seen	None seen	None seen	-
	Cast Average/10x Field	-	-	-	0
	Cast Type	-	-	-	-
	Cast Average/10x Field	-	-	-	0
	RBC's Average/45x Field	0-1	0	0-1	2-7
	WBC's Average/45x Field	0	0	0	0
	Epi cells Type	None seen	Squamous	None seen	-
	Epi cells Average/45x Field	-	1-2	-	0
	Epi cells Type	-	Transitional	-	-
	Epi cells Average/10x Field	-	0-1	-	0
	Crysal Type	None seen	-	None seen	-
	Crystal Severity/45x Field	-	0	0	0
	Crystal Type	-	-	-	-
	Crystal Severity/45x Field	-	-	-	0
	Bacteria Type	-	-	-	Cocci-motile
	Bacteria Severity 45x Field	0	1+	2+	2+
	Sperm Severity 45x Field	0	0	0	0
	Mucus Severity 45x Field	0	0	0	0
	Yeast Severity 45x Field	0	0	0	0

Table S4 (Continued). Urinalysis for NHPs over the course of the PPCN biocompatibility study. (n = 4; 2 female (F), 2 male (M)); RBC = red blood cells; WBC = white blood cells.

	**Monkey:**	**RH9148 (M)**			
	**Condition:**	Day of Tx			
	**Date:**	5/18/2020	5/26/2020	6/1/2020	6/8/2020
	**Day:**	0	8	14	21
Physical	**Appearance**	Clear	Clear	Clear	Hazy
	**Specific Gravity**	1.033	1.026	1.03	1.034
	**Color**	Yellow	Yellow	Yellow	Yellow
Dipstick Evaluation	**Leukocytes**	Negative	Negative	Negative	Negative
	**Nitrite**	Negative	Negative	Negative	Negative
	**pH**	6	7	8	6
	**Protein**	Trace	Trace	Trace	Trace
	**Glucose**	Normal	Normal	Normal	Normal
	**Ketones**	Negative	Negative	Negative	Negative
	**Urobilinogen**	Normal	Normal	Normal	Normal
	**Bilirubin**	Negative	Negative	Negative	Negative
	**Blood**	Negative	Negative	Trace	Negative
Sediment Evaluation	**Cast Type**	-	-	-	-
	**Cast Average/10x Field**	0	0	0	0
	**Cast Type**	-	-	-	-
	**Cast Average/10x Field**	-	-	-	0
	**RBC's Average/45x Field**	0	0	0	0
	**WBC's Average/45x Field**	0	0	0	0
	**Epi cells Type**	Squamous	Squamous	-	Transitional
	**Epi cells Average/45x Field**	0-2	0-1	0	0- large clumps
	**Epi cells Type**	-	-	-	Squamous
	**Epi cells Average/10x Field**	-	-	-	Rare
	**Crysal Type**	-	-	-	-
	**Crystal Severity/45x Field**	0	0	0	0
	**Crystal Type**	-	-	-	-
	**Crystal Severity/45x Field**	-	-	0	0
	**Bacteria Type**	-	-	-	Cocci-motile
	**Bacteria Severity 45x Field**	0	0	1+	3+
	**Sperm Severity 45x Field**	0	0	0	0
	**Mucus Severity 45x Field**	0	0	0	0
	**Yeast Severity 45x Field**	0	0	0	0

Table S4 (Continued). Urinalysis for NHPs over the course of the PPCN biocompatibility study. (n = 4; 2 female (F), 2 male (M)); RBC = red blood cells; WBC = white blood cells.

	Monkey:	RH9148 (M)			
	Condition:				Day of Retrieval
	Date:	6/15/2020	7/13/2020	8/10/2020	8/18/2020
	Day:	28	56	84	92
Physical	Appearance	Clear	Clear	Hazy	Clear
	Specific Gravity	1.017	1.025	1.027	1.026
	Color	Light Yellow	Yellow	Yellow	Yellow
Dipstick Evaluation	Leukocytes	Negative	Negative	Negative	Negative
	Nitrite	Negative	Negative	Negative	Negative
	pH	7	7	6	5
	Protein	Negative	Trace	Trace	Trace
	Glucose	Normal	Normal	Normal	Normal
	Ketones	Negative	Negative	Negative	+ (Small)
	Urobilinogen	Normal	Normal	Normal	Normal
	Bilirubin	Negative	Negative	Negative	Negative
	Blood	Trace	Trace	Negative	Negative
Sediment Evaluation	Cast Type	None seen	None seen	None seen	-
	Cast Average/10x Field	-	-	-	0
	Cast Type	-	-	-	-
	Cast Average/10x Field	-	-	-	0
	RBC's Average/45x Field	0-1	0-1	0	0-3
	WBC's Average/45x Field	0	0	0	0-1
	Epi cells Type	None seen	None seen	None seen	Transitional
	Epi cells Average/45x Field	-	-	-	0-3
	Epi cells Type	-	-	-	Squamous
	Epi cells Average/10x Field	-	-	-	0-4
	Crysal Type	None seen	None seen	None seen	-
	Crystal Severity/45x Field	-	-	-	0
	Crystal Type	-	-	-	-
	Crystal Severity/45x Field	-	-	-	0
	Bacteria Type	-	-	-	Cocci-motile
	Bacteria Severity 45x Field	0	1+	2+	1+
	Sperm Severity 45x Field	0	0	0	-
	Mucus Severity 45x Field	0	0	0	-
	Yeast Severity 45x Field	0	0	0	-

Table S5. Body temperature of nonhuman primates (NHPs) over the course of the PPCN biocompatibility study.
(n = 4; 2 female (F), 2 male (M)).

Body Temperature (°F)							
Condition:	Day of Tx						Day of Retrieval
Date:	5/18/2020	5/26/2020	6/1/2020	6/8/2020	6/15/2020	7/13/2020	8/18/2020
Day:	0	8	14	21	28	56	92
RH9138	100.7	99.9	101.1	101.4	101.4	102.2	100.4
RH9142	100.2	101.4	101.4	100.6	101.6	101.3	99.6
RH9145	98.8	102.1	101.1	101.5	101.7	102.3	99.4
RH9148	98.3	102.4	101.3	101.6	101.3	101.3	100.1

Table S6. Glucose stimulated insulin secretion (GSIS) assay on nonhuman primate (NHP) islets following total pancreatectomy (TP) and before islet autotransplantation (IAT) to the omentum with PPCN. (n = 2; 1 female (F), 1 male (M))

Monkey: RH9139 (F)				
Purified Islets (Post-Overnight Culture)				
	Insulin (µIU/mL) / 10 Islets / Hour			Insulin (µIU/mL)/ 10 Islets
	Low 2 mM Glucose	High 18 mM Glucose	SI	Acid Alcohol
1	361.2	377.3	1.0	17342.5
2	439.7	387.7	0.9	11465.7
3	353.1	384.7	1.1	14941.1
Average	384.7	383.2	1.0	14583.1
Std. Dev	47.8	5.4	0.1	2954.7

Monkey: RH9144 (M)				
Purified Islets (Post-Overnight Culture)				
	Insulin (µIU/mL) / 10 Islets / Hour			Insulin (µIU/mL)/ 10 Islets
	Low 2 mM Glucose	High 18 mM Glucose	SI	Acid Alcohol
1	151.4	403.7	2.7	8079.1
2	151.4	418.1	2.8	9184.0
3	154.7	299.4	1.9	6492.5
Average	152.5	373.7	2.5	7918.5
Std. Dev	1.9	64.8	0.5	1352.9

Table S7. Complete blood counts (CBCs) for nonhuman primates (NHPs) over the course of the total pancreatectomy (TP) followed by islet autotransplantation (TP-IAT) to the omentum with PPCN study.

(n = 2; 1 female (F), 1 male (M)); WBC = white blood cells; RBC = red blood cells; HGB = hemoglobin; HCT = hematocrit; MCV = mean corpuscular volume; MCH = mean corpuscular hemoglobin; MCHC = mean corpuscular hemoglobin concentration; PLT = platelets; MPV = mean platelet volume; RDW = red cell distribution width; NEUT = neutrophils; LYMPH = lymphocytes; MONO = monocytes; EOS = eosinophils; BASO = basophils; LUC = large unstained cells; RETIC =reticulocytes; MACRO = macrocytosis; HC-VAR = hemoglobin concentration variance; PLATCLMP = platelet clumps; HYPER = hyperchromia.

Monkey:	RH9139 (F)							
Condition:	Baseline	IVDTT Baseline	Pre-Transplant	Day of Transplant	Post-Transplant			Units
Date:	8/4/2022	8/9/2022	8/15/2022	8/16/2022	8/19/2022	8/23/2022	8/30/2022	
Day:	-12	-7	-1	0	3	7	14	
WBC	6.03	7.23	6.08	11.35	9.83	11.04	8.78	x10^3/µL
RBC	4.83	4.50	4.24	4.35	4.24	4.18	4.27	x10^6/µL
HGB	11.7	11.2	10.4	10.6	10.1	10.1	10.5	g/dL
HCT	38.3	35.5	33.0	34.4	33.4	33.8	33.4	%
MCV	79.4	78.9	77.8	79.0	78.9	80.8	78.2	fL
MCH	24.3	24.9	24.6	24.5	23.9	24.1	24.5	pg
MCHC	30.6	31.6	31.6	31	30.3	29.8	31.3	g/dL
PLT	291	264	334	340	372	576	369	x10^3/µL
MPV	7.3	8.1	7.2	7.1	8.8	7.8	8.0	fL
RDW	12.9	12.6	12.6	12.7	12.8	13.0	12.9	%
%NEUT	31.6	56.2**	40.6	80.5*****	63.0*****	62.1*****	48.2	%
%LYMPH	61.4	39.3	53.5	12.1***	30.1	29.1	41.4	%
%MONO	2.9	1.2	0.9**	1.6	3.8	1.2	1.8	%
%EOS	3.0	2.4	4.0	5.3	2.1	6.6***	7.5**	%
%BASO	0.2	0.4	0.1	0.1	0.1	0.4	0.2	%
%LUC	0.9	0.4	0.9	0.4	0.9	0.7	0.9	%
#NEUT	1.91	4.07	2.47	9.13*****	6.19	6.85*****	4.23	x10^3/µL
#LYMPH	3.70	2.84	3.25	1.37***	2.96	3.21	3.63	x10^3/µL
#MONO	0.17	0.09	0.05	0.18	0.38	0.13	0.16	x10^3/µL
#EOS	0.18	0.17	0.24	0.60***	0.21	0.72***	0.66**	x10^3/µL
#BASO	0.01	0.03	0.01	0.01	0.01	0.04	0.02	x10^3/µL
#LUC	0.06	0.03	0.06	0.05	0.08	0.08	0.08	x10^3/µL
%RETIC	1.21	1.67	2.03	2.02	2.68	2.61	3.1	%
#RETIC	58.4	75.0****	86.2	87.8	113.5	109.1	132.1	x10^9/µL

* Elevated/Low Levels, Comparable to baseline
** Elevated/Low Levels, Not of clinical concern
***Elevated/Low Levels likely due to stress response associated with surgery
****Reticulocytes not considered significant unless animal anemic; RBCs normal
*****Neutrophils: elevated due to surgical inflammation
. Elevated/ Low Levels possibly due to Immunosuppression/implanted biomaterial/encapsulated cells/cell products
_ Elevated levels possibly due to neutrophils being counted as eosinophils

Table S7 (Continued). CBCs for NHPs over the course of the TP-IAT to the omentum with PPCN study.

(n = 2; 1 female (F), 1 male (M)); WBC = white blood cells; RBC = red blood cells; HGB = hemoglobin; HCT = hematocrit; MCV = mean corpuscular volume; MCH = mean corpuscular hemoglobin; MCHC = mean corpuscular hemoglobin concentration; PLT = platelets; MPV = mean platelet volume; RDW = red cell distribution width; NEUT = neutrophils; LYMPH = lymphocytes; MONO = monocytes; EOS = eosinophils; BASO = basophils; LUC = large unstained cells; RETIC =reticulocytes; MACRO = macrocytosis; HC-VAR = hemoglobin concentration variance; PLATCLMP = platelet clumps; HYPER = hyperchromia.

Monkey:	RH9139 (F)		
Condition:	Post-Transplant		Units
Date:	9/6/2022	9/13/2022	
Day:	21	28	
WBC	7.95	7.69	x10^3/µL
RBC	4.55	4.43	x10^6/µL
HGB	11.0	10.8	g/dL
HCT	36.0	35.4	%
MCV	79.1	79.9	fL
MCH	24.2	24.3	pg
MCHC	30.6	30.4	g/dL
PLT	313	335	x10^3/µL
MPV	8.9	8.3	fL
RDW	13.1	14.0	%
%NEUT	49.6	40.4	%
%LYMPH	43.9	52.9	%
%MONO	3.7	1.9	%
%EOS	1.4	3.4	%
%BASO	0.3	0.4	%
%LUC	1.1	1	%
#NEUT	3.94	3.11	x10^3/µL
#LYMPH	3.49	4.07	x10^3/µL
#MONO	0.29	0.15	x10^3/µL
#EOS	0.12	0.26	x10^3/µL
#BASO	0.02	0.03	x10^3/µL
#LUC	0.08	0.08	x10^3/µL
%RETIC	3.3	2.72	%
#RETIC	150.2	120.8	x10^9/µL

* Elevated/Low Levels, Comparable to baseline
** Elevated/Low Levels, Not of clinical concern
***Elevated/Low Levels likely due to stress response associated with surgery
****Reticulocytes not considered significant unless animal anemic; RBCs normal
*****Neutrophils: elevated due to surgical inflammation
. Elevated/ Low Levels possibly due to Immunosuppression/implanted biomaterial/encapsulated cells/cell products
_ Elevated levels possibly due to neutrophils being counted as eosinophils

Table S7 (Continued). CBCs for NHPs over the course of the TP-IAT to the omentum with PPCN study.

(n = 2; 1 female (F), 1 male (M)); WBC = white blood cells; RBC = red blood cells; HGB = hemoglobin; HCT = hematocrit; MCV = mean corpuscular volume; MCH = mean corpuscular hemoglobin; MCHC = mean corpuscular hemoglobin concentration; PLT = platelets; MPV = mean platelet volume; RDW = red cell distribution width; NEUT = neutrophils; LYMPH = lymphocytes; MONO = monocytes; EOS = eosinophils; BASO = basophils; LUC = large unstained cells; RETIC =reticulocytes; MACRO = macrocytosis; HC-VAR = hemoglobin concentration variance; PLATCLMP = platelet clumps; HYPER = hyperchromia.

Monkey:	RH9139 (F)								
Condition:	Post-Transplant		IVDTT Post-Transplant	Glucagon Post-Transplant	Post-Transplant		IVDTT Post-Transplant	Post-Transplant	Units
Date:	9/6/2022	9/13/2022	9/15/2022	9/20/2022	9/27/2022	10/4/2022	10/10/2022	10/18/2022	
Day:	21	28	30	35	42	49	55	63	
WBC	7.95	7.69	9.18	8.01	10.26	8.75	6.59	13.02	x10^3/µL
RBC	4.55	4.43	4.39	4.57	4.57	4.59	4.47	4.27	x10^6/µL
HGB	11.0	10.8	10.9	11.0	11.3	11.1	11.0	10.5	g/dL
HCT	36.0	35.4	34.6	36.0	36.6	36.3	35.4	34.6	%
MCV	79.1	79.9	79.0	78.8	80.0	79.1	79.3	81.0	fL
MCH	24.2	24.3	24.8	24.1	24.7	24.2	24.6	24.7	pg
MCHC	30.6	30.4	31.4	30.6	30.9	30.6	31	30.4	g/dL
PLT	313	335	332	377	406	363	347	427	x10^3/µL
MPV	8.9	8.3	8.1	8.1	8.1	8.0	7.7	8.1	fL
RDW	13.1	14.0	14.2	14.3	13.8	14.2	14.2	14.1	%
%NEUT	49.6	40.4	51.7	50.3	37.9	46.7	74.2**	68.7**	%
%LYMPH	43.9	52.9	43.4	42.4	52.8	47.5	23**	27.5	%
%MONO	3.7	1.9	1.4	3.3	2.3	2.5	1.8	1.9	%
%EOS	1.4	3.4	2.3	2.7	5.7	1.9	0.2	1.1	%
%BASO	0.3	0.4	0.2	0.3	0.3	0.4	0	0.2	%
%LUC	1.1	1	0.9	1	0.9	1	0.7	0.5	%
#NEUT	3.94	3.11	4.75	4.03	3.89	4.09	4.89	8.95**	x10^3/µL
#LYMPH	3.49	4.07	3.98	3.4	5.42	4.16	1.52	3.59	x10^3/µL
#MONO	0.29	0.15	0.13	0.26	0.23	0.22	0.12	0.25	x10^3/µL
#EOS	0.12	0.26	0.21	0.22	0.58**	0.17	0.02	0.14	x10^3/µL
#BASO	0.02	0.03	0.02	0.02	0.03	0.03	0	0.02	x10^3/µL
#LUC	0.08	0.08	0.08	0.08	0.09	0.09	0.04	0.07	x10^3/µL
%RETIC	3.3	2.72	1.98	2.17	2.37	2.7	2.13	2.69	%
#RETIC	150.2	120.8	86.7	98.9	108.4	123.8	94.9	114.6	x10^9/µL

* Elevated/Low Levels, Comparable to baseline
** Elevated/Low Levels, Not of clinical concern
***Elevated/Low Levels likely due to stress response associated with surgery
****Reticulocytes not considered significant unless animal anemic; RBCs normal
*****Neutrophils: elevated due to surgical inflammation
. Elevated/ Low Levels possibly due to Immunosuppression/implanted biomaterial/encapsulated cells/cell products
_ Elevated levels possibly due to neutrophils being counted as eosinophils

Table S7 (Continued). CBCs for NHPs over the course of the TP-IAT to the omentum with PPCN study.

(n = 2; 1 female (F), 1 male (M)); WBC = white blood cells; RBC = red blood cells; HGB = hemoglobin; HCT = hematocrit; MCV = mean corpuscular volume; MCH = mean corpuscular hemoglobin; MCHC = mean corpuscular hemoglobin concentration; PLT = platelets; MPV = mean platelet volume; RDW = red cell distribution width; NEUT = neutrophils; LYMPH = lymphocytes; MONO = monocytes; EOS = eosinophils; BASO = basophils; LUC = large unstained cells; RETIC =reticulocytes; MACRO = macrocytosis; HC-VAR = hemoglobin concentration variance; PLATCLMP = platelet clumps; HYPER = hyperchromia.

Monkey:	RH9139 (F)							
Condition:	Post-Transplant					IVDTT Post-Transplant	Survival Omentectomy Post-Transplant	
Date:	10/25/2022	11/1/2022	11/7/2022	11/15/2022	11/22/2022	11/29/2022	12/2/2022	
Day:	70	77	83	91	98	105	108	Units
WBC	13.6	11.16	6.69	7.98	9.24	8.97	5.68	x10^3/µL
RBC	4.56	4.46	4.44	4.47	4.66	4.69	4.34	x10^6/µL
HGB	11.2	11.1	11.0	10.8	11.4	11.3	10.7	g/dL
HCT	35.8	35.3	34.3	35.3	37.0	37.4	34.2	%
MCV	78.5	79.0	77.2	79.0	79.4	79.6	78.7	fL
MCH	24.4	24.9	24.8	24.2	24.5	24.1	24.6	pg
MCHC	31.1	31.5	32.2	30.6	30.9	30.2	31.3	g/dL
PLT	429	362	388	393	376	386	356	x10^3/µL
MPV	7.8	7.9	7.9	8.4	7.9	8.1	8.0	fL
RDW	14.5	14.4	14.4	14.5	14.5	14.5	14.8	%
%NEUT	68.1**	62.4**	48.9	69.3**	55.5**	54.4**	43.5	%
%LYMPH	29.0	32.1	44.6	26.5	40.3	40.7	50.7	%
%MONO	2.1	1.9	1.5	2.1	2.8	2.6	2.9	%
%EOS	0.3	2.6	3.9	1.3	0.4	1.2	1.2	%
%BASO	0.2	0.2	0.2	0	0.1	0.1	0.1	%
%LUC	0.3	0.8	0.9	0.8	0.9	1	1.6	%
#NEUT	9.27**	6.97**	3.27	5.53	5.13	4.88	2.47	x10^3/µL
#LYMPH	3.95	3.59	2.98	2.11	3.72	3.65	2.88	x10^3/µL
#MONO	0.29	0.21	0.1	0.17	0.26	0.23	0.16	x10^3/µL
#EOS	0.04	0.29	0.26	0.10	0.03	0.11	0.07	x10^3/µL
#BASO	0.02	0.02	0.02	0	0.01	0.01	0.01	x10^3/µL
#LUC	0.04	0.08	0.06	0.06	0.09	0.09	0.09	x10^3/µL
%RETIC	2.13	2.35	1.69	2.95	1.92	2.13	2.27	%
#RETIC	97.2	104.7	75.2****	131.9	89.4	99.8	98.5	x10^9/µL

* Elevated/Low Levels, Comparable to baseline
** Elevated/Low Levels, Not of clinical concern
***Elevated/Low Levels likely due to stress response associated with surgery
****Reticulocytes not considered significant unless animal anemic; RBCs normal
*****Neutrophils: elevated due to surgical inflammation
. Elevated/ Low Levels possibly due to Immunosuppression/implanted biomaterial/encapsulated cells/cell products
_ Elevated levels possibly due to neutrophils being counted as eosinophils

Table S7 (Continued). CBCs for NHPs over the course of the TP-IAT to the omentum with PPCN study.

(n = 2; 1 female (F), 1 male (M)); WBC = white blood cells; RBC = red blood cells; HGB = hemoglobin; HCT = hematocrit; MCV = mean corpuscular volume; MCH = mean corpuscular hemoglobin; MCHC = mean corpuscular hemoglobin concentration; PLT = platelets; MPV = mean platelet volume; RDW = red cell distribution width; NEUT = neutrophils; LYMPH = lymphocytes; MONO = monocytes; EOS = eosinophils; BASO = basophils; LUC = large unstained cells; RETIC =reticulocytes; MACRO = macrocytosis; HC-VAR = hemoglobin concentration variance; PLATCLMP = platelet clumps; HYPER = hyperchromia.

Monkey:	RH9139 (F)		
Condition:	IVDTT Post - Omentectomy	Termination & Necropsy	
Date:	11/29/2022	12/2/2022	
Day:	105	108	Units
WBC	8.97	5.68	x10^3/µL
RBC	4.69	4.34	x10^6/µL
HGB	11.3	10.7	g/dL
HCT	37.4	34.2	%
MCV	79.6	78.7	fL
MCH	24.1	24.6	pg
MCHC	30.2	31.3	g/dL
PLT	386	356	x10^3/µL
MPV	8.1	8.0	fL
RDW	14.5	14.8	%
%NEUT	54.4**	43.5	%
%LYMPH	40.7	50.7	%
%MONO	2.6	2.9	%
%EOS	1.2	1.2	%
%BASO	0.1	0.1	%
%LUC	1	1.6	%
#NEUT	4.88	2.47	x10^3/µL
#LYMPH	3.65	2.88	x10^3/µL
#MONO	0.23	0.16	x10^3/µL
#EOS	0.11	0.07	x10^3/µL
#BASO	0.01	0.01	x10^3/µL
#LUC	0.09	0.09	x10^3/µL
%RETIC	2.13	2.27	%
#RETIC	99.8	98.5	x10^9/µL

* Elevated/Low Levels, Comparable to baseline
** Elevated/Low Levels, Not of clinical concern
***Elevated/Low Levels likely due to stress response associated with surgery
****Reticulocytes not considered significant unless animal anemic; RBCs normal
*****Neutrophils: elevated due to surgical inflammation
. Elevated/ Low Levels possibly due to Immunosuppression/implanted biomaterial/encapsulated cells/cell products
_ Elevated levels possibly due to neutrophils being counted as eosinophils

Table S7 (Continued). CBCs for NHPs over the course of the TP-IAT to the omentum with PPCN study.

(n = 2; 1 female (F), 1 male (M)); WBC = white blood cells; RBC = red blood cells; HGB = hemoglobin; HCT = hematocrit; MCV = mean corpuscular volume; MCH = mean corpuscular hemoglobin; MCHC = mean corpuscular hemoglobin concentration; PLT = platelets; MPV = mean platelet volume; RDW = red cell distribution width; NEUT = neutrophils; LYMPH = lymphocytes; MONO = monocytes; EOS = eosinophils; BASO = basophils; LUC = large unstained cells; RETIC =reticulocytes; MACRO = macrocytosis; HC-VAR = hemoglobin concentration variance; PLATCLMP = platelet clumps; HYPER = hyperchromia.

Monkey:	RH9144 (M)							
Condition:	Baseline	Pre-Transplant	Post-Transplant					
Date:	-6	0	7	7/21/2022	21	28	30	
Day:	6/15/2022	6/20/2022	6/28/2022	8.95	7/12/2022	7/19/2022	7/21/2022	Units
WBC	7.1	10.19	27.72***	5.05	10.56	11.39	8.95	x10^3/µL
RBC	5.31	4.80	4.38	11.8	4.94	5.22	5.05	x10^6/µL
HGB	12.3	11.1	10.1	38.0	11.3	12.1	11.8	g/dL
HCT	38.8	34.7	33.0	75.1	36.7	38.9	38.0	%
MCV	73.0	72.4	75.3	23.3	74.2	74.5	75.1	fL
MCH	23.2	23.1	23.1	31	23.0	23.1	23.3	pg
MCHC	31.8	32	30.7	500	30.9	31	31	g/dL
PLT	393	398	882***	8.1	639	511	500	x10^3/µL
MPV	7.5	7.0	6.8	12.7	7.3	7.5	8.1	fL
RDW	12.2	12.0	12.9	42.1	12.8	12.6	12.7	%
%NEUT	55.5	63.5*	89.2*****	45.3	60.9*	71.9*.*	42.1	%
%LYMPH	39.4	31.2	6.1***	3.9	28.1	22.4*.*	45.3	%
%MONO	3.7	2.8	2.4	6.2	3.1	2.3	3.9	%
%EOS	0.2	1.2	1.6	0.2	5.9	2.0	6.2	%
%BASO	0.2	0.2	0.1	2.3	0.5	0.1	0.2	%
%LUC	1	1.1	0.6	3.77	1.5	1.4	2.3	%
#NEUT	3.94	6.47**	24.72*****	4.05	6.44*.*	8.19*.*	3.77	x10^3/µL
#LYMPH	2.80	3.18	1.69	0.35	2.97	2.55	4.05	x10^3/µL
#MONO	0.27	0.28	0.68	0.56*.*	0.33	0.26	0.35	x10^3/µL
#EOS	0.01	0.13	0.44	0.02	0.63*.*	0.23	0.56*.*	x10^3/µL
#BASO	0.02	0.02	0.03	0.2	0.05	0.01	0.02	x10^3/µL
#LUC	0.07	0.11	0.17	2.53	0.16	0.16	0.2	x10^3/µL
%RETIC	1.03	1.25	3.66****	127.9	2.77	2.39	2.53	%
#RETIC	54.4	59.7**	160.6	30	137.1	124.9	127.9	x10^9/µL

* Elevated/Low Levels, Comparable to baseline
**Elevated/ Low Levels, Not of clinical concern
***Elevated or Low Levels likely due to stress response associated with surgery
****Reticulocytes not considered significant unless animal anemic; RBCs normal
*****Neutrophils: elevated due to surgical inflammation
. Elevated/ Low Levels possibly due to Immunosuppression/implanted biomaterial/encapsulated cells/cell products
_ Elevated levels possibly due to neutrophils being counted as eosinophils

Table S7 (Continued). CBCs for NHPs over the course of the TP-IAT to the omentum with PPCN study.

(n = 2; 1 female (F), 1 male (M)); WBC = white blood cells; RBC = red blood cells; HGB = hemoglobin; HCT = hematocrit; MCV = mean corpuscular volume; MCH = mean corpuscular hemoglobin; MCHC = mean corpuscular hemoglobin concentration; PLT = platelets; MPV = mean platelet volume; RDW = red cell distribution width; NEUT = neutrophils; LYMPH = lymphocytes; MONO = monocytes; EOS = eosinophils; BASO = basophils; LUC = large unstained cells; RETIC =reticulocytes; MACRO = macrocytosis; HC-VAR = hemoglobin concentration variance; PLATCLMP = platelet clumps; HYPER = hyperchromia.

Monkey:	RH9144 (M)							
Condition:	Post-Transplant							
Date:	42	49	63	70	77	84	91	
Day:	8/2/2022	8/9/2022	8/23/2022	8/30/2022	9/6/2022	9/13/2022	9/20/2022	Units
WBC	9.72	12.25	11.64	11.8	10.91	12.37	12.69	x10^3/µL
RBC	5.11	5.36	5.13	5.39	5.35	5.40	5.25	x10^6/µL
HGB	12.1	12.7	12.1	12.7	12.4	12.6	12.2	g/dL
HCT	38.5	40.8	39.1	39.8	39.6	40.9	38.9	%
MCV	75.4	76.1	76.1	73.8	74.1	75.7	74.0	fL
MCH	23.7	23.6	23.5	23.6	23.3	23.3	23.3	pg
MCHC	31.4	31	30.8	32	31.4	30.8	31.4	g/dL
PLT	549	492	492	503	493	460	462	x10^3/µL
MPV	7.1	9.0	8.0	8.3	9.0	8.8	7.9	fL
RDW	12.7	12.5	12.7	12.6	12.7	14.3	14.5	%
%NEUT	49.2	51.6	48.1	44.4	44.8	58.6*	55.9*	%
%LYMPH	41.5	41.3	45.5	48.5	48.0	34.8	36.1	%
%MONO	3.5	2.3	2.0	3.0	2.5	2.0	2.7	%
%EOS	3.9	3.0	2.8	2.8	2.7	2.9	4.2	%
%BASO	0.3	0.5	0.4	0.2	0.4	0.4	0.2	%
%LUC	1.6	1.3	1.2	1.1	1.5	1.3	1	%
#NEUT	4.79	6.32*.*	5.59	5.24	4.89	7.25*.*	7.09*.*	x10^3/µL
#LYMPH	4.04	5.06	5.3	5.73	5.24	4.3	4.57	x10^3/µL
#MONO	0.34	0.28	0.23	0.35	0.28	0.25	0.34	x10^3/µL
#EOS	0.37	0.37	0.32	0.33	0.29	0.36	0.54**	x10^3/µL
#BASO	0.03	0.06	0.04	0.03	0.05	0.05	0.02	x10^3/µL
#LUC	0.15	0.16	0.14	0.13	0.16	0.16	0.13	x10^3/µL
%RETIC	2.25	2.16	2.1	1.74	1.86	1.43	1.22	%
#RETIC	115	115.5	107.9	93.7	99.8	77.5	64****	x10^9/µL

* Elevated/Low Levels, Comparable to baseline
**Elevated/ Low Levels, Not of clinical concern
***Elevated or Low Levels likely due to stress response associated with surgery
****Reticulocytes not considered significant unless animal anemic; RBCs normal
*****Neutrophils: elevated due to surgical inflammation
. Elevated/ Low Levels possibly due to Immunosuppression/implanted biomaterial/encapsulated cells/cell products
_ Elevated levels possibly due to neutrophils being counted as eosinophils

Table S7 (Continued). CBCs for NHPs over the course of the TP-IAT to the omentum with PPCN study.

(n = 2; 1 female (F), 1 male (M)); WBC = white blood cells; RBC = red blood cells; HGB = hemoglobin; HCT = hematocrit; MCV = mean corpuscular volume; MCH = mean corpuscular hemoglobin; MCHC = mean corpuscular hemoglobin concentration; PLT = platelets; MPV = mean platelet volume; RDW = red cell distribution width; NEUT = neutrophils; LYMPH = lymphocytes; MONO = monocytes; EOS = eosinophils; BASO = basophils; LUC = large unstained cells; RETIC =reticulocytes; MACRO = macrocytosis; HC-VAR = hemoglobin concentration variance; PLATCLMP = platelet clumps; HYPER = hyperchromia.

Monkey:	**RH9144** (M)				
**Condition:**	Post-Transplant			Post-Omentectomy	**Units**
**Date:**	98	105	112	118	
**Day:**	9/27/2022	10/4/2022	10/11/2022	10/17/2022	
**WBC**	11.16	11.65	10.61	11.11	x10^3/µL
**RBC**	5.39	5.71	5.66	5.55	x10^6/µL
**HGB**	12.8	13.6	13.3	13.0	g/dL
**HCT**	40.5	42.7	41.9	42.6	%
**MCV**	75.2	74.8	74.0	76.9	fL
**MCH**	23.8	23.9	23.5	23.4	pg
**MCHC**	31.7	31.9	31.8	30.5	g/dL
**PLT**	473	535	476	501	x10^3/µL
**MPV**	9.4	9.1	7.5	8.4	fL
**RDW**	14.1	14.6	14.2	13.7	%
**%NEUT**	44.4	44	55.8*	83*****	%
**%LYMPH**	45.1	47.3	38.8	10.7***	%
**%MONO**	3.7	3.4	3.0	3.7	%
**%EOS**	5.2	3.0	0.6	1.0	%
**%BASO**	0.2	0.5	0.2	0.1	%
**%LUC**	1.4	1.8	1.6	1.5	%
**#NEUT**	4.96	5.13	5.92	9.22*****	x10^3/µL
**#LYMPH**	5.03	5.51	4.12	1.19***	x10^3/µL
**#MONO**	0.41	0.4	0.32	0.41	x10^3/µL
**#EOS**	0.59**	0.35	0.06	0.11	x10^3/µL
**#BASO**	0.02	0.05	0.02	0.01	x10^3/µL
**#LUC**	0.16	0.21	0.17	0.16	x10^3/µL
**%RETIC**	1.6	1.76	1.35	1.35	%
**#RETIC**	86.4	100.3	76.7****	75.1****	x10^9/µL

* Elevated/Low Levels, Comparable to baseline
**Elevated/ Low Levels, Not of clinical concern
***Elevated or Low Levels likely due to stress response associated with surgery
****Reticulocytes not considered significant unless animal anemic; RBCs normal
*****Neutrophils: elevated due to surgical inflammation
. Elevated/ Low Levels possibly due to Immunosuppression/implanted biomaterial/encapsulated cells/cell products
_ Elevated levels possibly due to neutrophils being counted as eosinophils

Table S7 (Continued). CBCs for NHPs over the course of the TP-IAT to the omentum with PPCN study.
(n = 2; 1 female (F), 1 male (M)); WBC = white blood cells; RBC = red blood cells; HGB = hemoglobin; HCT = hematocrit; MCV = mean corpuscular volume; MCH = mean corpuscular hemoglobin; MCHC = mean corpuscular hemoglobin concentration; PLT = platelets; MPV = mean platelet volume; RDW = red cell distribution width; NEUT = neutrophils; LYMPH = lymphocytes; MONO = monocytes; EOS = eosinophils; BASO = basophils; LUC = large unstained cells; RETIC =reticulocytes; MACRO = macrocytosis; HC-VAR = hemoglobin concentration variance; PLATCLMP = platelet clumps; HYPER = hyperchromia.

Monkey:	RH9144 (M)		
Condition:	Post-Transplant	Post-Omentectomy	
Date:	112	118	
Day:	10/11/2022	10/17/2022	Units
WBC	10.61	11.11	x10^3/µL
RBC	5.66	5.55	x10^6/µL
HGB	13.3	13.0	g/dL
HCT	41.9	42.6	%
MCV	74.0	76.9	fL
MCH	23.5	23.4	pg
MCHC	31.8	30.5	g/dL
PLT	476	501	x10^3/µL
MPV	7.5	8.4	fL
RDW	14.2	13.7	%
%NEUT	55.8*	83*****	%
%LYMPH	38.8	10.7***	%
%MONO	3.0	3.7	%
%EOS	0.6	1.0	%
%BASO	0.2	0.1	%
%LUC	1.6	1.5	%
#NEUT	5.92	9.22*****	x10^3/µL
#LYMPH	4.12	1.19***	x10^3/µL
#MONO	0.32	0.41	x10^3/µL
#EOS	0.06	0.11	x10^3/µL
#BASO	0.02	0.01	x10^3/µL
#LUC	0.17	0.16	x10^3/µL
%RETIC	1.35	1.35	%
#RETIC	76.7****	75.1****	x10^9/µL

* Elevated/Low Levels, Comparable to baseline
**Elevated/ Low Levels, Not of clinical concern
***Elevated or Low Levels likely due to stress response associated with surgery
****Reticulocytes not considered significant unless animal anemic; RBCs normal
*****Neutrophils: elevated due to surgical inflammation
. Elevated/ Low Levels possibly due to Immunosuppression/implanted biomaterial/encapsulated cells/cell products
_ Elevated levels possibly due to neutrophils being counted as eosinophils

Table S8. Blood chemistry panel for nonhuman primates (NHPs) over the course of the total pancreatectomy (TP) followed by islet autotransplantation (TP-IAT) to the omentum with PPCN study.

(n = 2; 1 female (F), 1 male (M)); ALT = alanine transaminase; AST = aspartate aminotransferase; BUN = blood urea nitrogen; GGT = gamma-glutamyl transferase; I = inorganic.

**Monkey:**	**RH9139 (F)**								
**Condition:**	Baseline	IVDTT Baseline	Pre-Transplant	Day of Transplant	Post-Transplant				
**Date:**	8/4/2022	8/9/2022	8/15/2022	8/16/2022	8/19/2022	8/23/2022	8/30/2022	9/6/2022	
**Day:**	-12	-7	-1	0	3	7	14	21	**Units**
Albumin	4.198	4.374	4.047	3.516	3.361	3.788	3.806	3.856	g/dL
Alkaline Phosphatase	126	135	123	115	126	151	140	157	U/L
ALT	20	37*****	32*****	51*****	44*****	93*****	72*****	75*****	U/L
AST	19	25	22	88***	24	36	35	37	U/L
Urea Nitrogen (BUN)	22	11	17	19	13	15	15	15	mg/dL
Calcium	9.88	9.17	9.14	8.39	9.06	9.14	9.73	9.23	mg/dL
Creatinine	0.7784	0.7131	0.663	0.7321	0.7134	0.6466	0.6045	0.636	mg/dL
GGT	38	39	34	33	31	37	40	46	U/L
Glucose	102	78	89	55	136	96	98	175	mg/dL
I. Phosphorous	3.6	5.3	5.3	3.5	4.4	4.7	3.9	4.3	mg/dL
Total Bilirubin	0.13	0.15	0.14	0.16	0.12	0.08	0.12	0.16	mg/dL
Total Protein	6.087	6.268	5.987	5.047***	5.493***	6.149	5.625	5.911	g/dL
Sodium	144	142	144	145	143	153	144	141	mmol/L
Potassium	3.8	3.1*	3.4*	3.0**	4.1	3.9	3.5	3.5	mmol/L
Chloride	105	103*	104*	108	103*	108	105*	103*	mmol/L
Globulin	1.889	1.894*	1.94*	1.531**	2.132**	2.361**	1.819*	2.055*	
Alb/Glob Ratio	2	2	2	2	2	2	2	2	

* Elevated/Low Levels, Comparable to baseline

** Elevated/Low Levels, Not of clinical concern

***Elevated/Low Levels likely due to stress response associated with surgery

****Muscle component, indicative of possible damage to muscle - may be related to anesthesia injections or damage to muscle during surgery

*****Elevated ALT levels are considered of clinical concern only if the levels are more than 100 IU/L.

******Elevated Globulin levels than Albumin levels are indicative of inflammation, possibly due to implanted microcapsules, encapsulated cells or cell products

*******Elevated levels, Not of clinical concern; Possibly mild inflammation of liver but transient

. Elevated/ Low Levels likely due to Immunosuppression/implanted biomaterial/encapsulated cells/cell products

_ Elevated due to diabetic condition of the animal

Table S8 (Continued). Blood chemistry panel for NHPs over the course of the TP-IAT to the omentum with PPCN study.
(n = 2; 1 female (F), 1 male (M)); ALT = alanine transaminase; AST = aspartate aminotransferase; BUN = blood urea nitrogen; GGT = gamma-glutamyl transferase; I = inorganic.

Monkey:	RH9139 (F)							
Condition:	Post-Transplant	IVDTT Post-Transplant	Glucagon Post-Transplant	Post-Transplant		IVDTT Post-Transplant	Post-Transplant	
Date:	9/13/2022	9/15/2022	9/20/2022	9/27/2022	10/4/2022	10/10/2022	10/18/2022	
Day:	28	30	35	42	49	55	63	Units
Albumin	3.724	3.764	3.342	3.351	3.621	3.895	3.559	g/dL
Alkaline Phosphatase	141	138	122	130	113	117	126	U/L
ALT	57*****	81*****	36*****	25*****	36*****	39*****	45*****	U/L
AST	32	43	25	31	27	37	29	U/L
Urea Nitrogen (BUN)	13	13	20	23	20	18	21	mg/dL
Calcium	9.21	9.38	8.52	9.15	8.7	8.62	9.03	mg/dL
Creatinine	0.5912	0.5242	0.6451	0.6927	0.5705	0.5513	0.526	mg/dL
GGT	41	42	33	32	33	34	37	U/L
Glucose	123	142	80	118	113	153	206	mg/dL
I. Phosphorous	4.7	5.8	4.9	3.9	5.2	5.3	3.8	mg/dL
Total Bilirubin	0.16	0.14	0.15	0.11	0.11	0.16	0.1	mg/dL
Total Protein	5.621	5.652	5.157*	5.217*	5.529*	5.728	6.739	g/dL
Sodium	144	143	142	157	144	131*	140	mmol/L
Potassium	3.4	3.7	3.1*	4	3.2*	3.4	4	mmol/L
Chloride	105*	106*	103*	112	103*	97*	105*	mmol/L
Globulin	1.897*	1.888*	1.815*	1.866*	1.908*	1.833*	-	
Alb/Glob Ratio	2	2	2	2	2	2	-	

* Elevated/Low Levels, Comparable to baseline
** Elevated/Low Levels, Not of clinical concern
***Elevated/Low Levels likely due to stress response associated with surgery
****Muscle component, indicative of possible damage to muscle - may be related to anesthesia injections or damage to muscle during surgery
*****Elevated ALT levels are considered of clinical concern only if the levels are more than 100 IU/L.
******Elevated Globulin levels than Albumin levels are indicative of inflammation, possibly due to implanted microcapsules, encapsulated cells or cell products
*******Elevated levels, Not of clinical concern; Possibly mild inflammation of liver but transient
. Elevated/ Low Levels likely due to Immunosuppression/implanted biomaterial/encapsulated cells/cell products
_ Elevated due to diabetic condition of the animal

Table S8 (Continued). Blood chemistry panel for NHPs over the course of the TP-IAT to the omentum with PPCN study.
(n = 2; 1 female (F), 1 male (M)); ALT = alanine transaminase; AST = aspartate aminotransferase; BUN = blood urea nitrogen; GGT = gamma-glutamyl transferase; I = inorganic.

**Monkey:**	**RH9139 (F)**						
**Condition:**	Post-Transplant					IVDTT Post-Transplant	
**Date:**	10/25/2022	11/1/2022	11/7/2022	11/15/2022	11/22/2022	11/29/2022	
**Day:**	70	77	83	91	98	105	**Units**
Albumin	3.434	3.395	3.486	3.403	3.289	3.306	g/dL
Alkaline Phosphatase	104	103	111	101	96	116	U/L
ALT	30*****	27*****	32*****	36*****	29*****	31*****	U/L
AST	28	23	24	28	23	28	U/L
Urea Nitrogen (BUN)	25	25	12	17	20	19	mg/dL
Calcium	9.16	8.36	8.38	8.28	8.26	8.22	mg/dL
Creatinine	0.5724	0.5328	0.5241	0.4902	0.4688	0.5528	mg/dL
GGT	32	32	33	32	30	34	U/L
Glucose	121	110	73	68	142	149	mg/dL
I. Phosphorous	4	3.7	5.8	5.4	4.3	4.5	mg/dL
Total Bilirubin	0.14	0.12	0.16	0.14	0.13	0.16	mg/dL
Total Protein	5.16*	5.201*	5.559*	5.2*	5.008*	4.94*	g/dL
Sodium	142	142	143	143	139*	140	mmol/L
Potassium	3.6	3.3	3.3	3.1*	3.3	3.2*	mmol/L
Chloride	105*	106*	104*	104*	103*	104*	mmol/L
Globulin	1.726*	1.806*	2.073*	1.797*	1.719*	1.634**	
Alb/Glob Ratio	2	2	2	2	2	2	

* Elevated/Low Levels, Comparable to baseline
** Elevated/Low Levels, Not of clinical concern
***Elevated/Low Levels likely due to stress response associated with surgery
****Muscle component, indicative of possible damage to muscle - may be related to anesthesia injections or damage to muscle during surgery
*****Elevated ALT levels are considered of clinical concern only if the levels are more than 100 IU/L.
******Elevated Globulin levels than Albumin levels are indicative of inflammation, possibly due to implanted microcapsules, encapsulated cells or cell products
*******Elevated levels, Not of clinical concern; Possibly mild inflammation of liver but transient
. Elevated/ Low Levels likely due to Immunosuppression/implanted biomaterial/encapsulated cells/cell products
_ Elevated due to diabetic condition of the animal

Table S8 (Continued). Blood chemistry panel for NHPs over the course of the TP-IAT to the omentum with PPCN study.
(n = 2; 1 female (F), 1 male (M)); ALT = alanine transaminase; AST = aspartate aminotransferase; BUN = blood urea nitrogen; GGT = gamma-glutamyl transferase; I = inorganic.

Monkey:	RH9139 (F)			
Condition:	Survival Omentectomy Post-Transplant	IVDTT Post - Omentectomy	Termination & Necropsy	
Date:	12/2/2022	12/5/2022	12/8/2022	
Day:	108	111	114	Units
Albumin	3.359	3.201	3.233	g/dL
Alkaline Phosphatase	107	166	117	U/L
ALT	39*****	43*****	43*****	U/L
AST	29	30	40	U/L
Urea Nitrogen (BUN)	14	26	14	mg/dL
Calcium	8.16	8.23	8.37	mg/dL
Creatinine	0.4947	0.6162	0.4789	mg/dL
GGT	34	34	35	U/L
Glucose	56	199	89	mg/dL
I. Phosphorous	5.6	6	5.1	mg/dL
Total Bilirubin	0.14	0.24	0.14	mg/dL
Total Protein	5.399*	5.574*	5.426*	g/dL
Sodium	141	137*	145	mmol/L
Potassium	3.1*	3.6	3.0**	mmol/L
Chloride	102*	94*	102*	mmol/L
Globulin	2.04*	2.373**	2.193**	
Alb/Glob Ratio	2	1	1	

* Elevated/Low Levels, Comparable to baseline
** Elevated/Low Levels, Not of clinical concern
***Elevated/Low Levels likely due to stress response associated with surgery
****Muscle component, indicative of possible damage to muscle - may be related to anesthesia injections or damage to muscle during surgery
*****Elevated ALT levels are considered of clinical concern only if the levels are more than 100 IU/L.
******Elevated Globulin levels than Albumin levels are indicative of inflammation, possibly due to implanted microcapsules, encapsulated cells or cell products
*******Elevated levels, Not of clinical concern; Possibly mild inflammation of liver but transient
. Elevated/ Low Levels likely due to Immunosuppression/implanted biomaterial/encapsulated cells/cell products
_ Elevated due to diabetic condition of the animal

Table S8 (Continued). Blood chemistry panel for NHPs over the course of the TP-IAT to the omentum with PPCN study.
(n = 2; 1 female (F), 1 male (M)); ALT = alanine transaminase; AST = aspartate aminotransferase; BUN = blood urea nitrogen; GGT = gamma-glutamyl transferase; I = inorganic.

Monkey:	RH9144 (M)							
Condition:	Pre-Transplant		Post-Transplant					
Date:	-6	0	7	14	21	28	30	
Day:	6/15/2022	6/20/2022	6/28/2022	7/5/2022	7/12/2022	7/19/2022	7/21/2022	Units
Albumin	4.771	4.426	3.693	3.912	4.096	4.367	4.283	g/dL
Alkaline Phosphatase	391	338	336	327	365	439	426	U/L
ALT	32*****	31*****	27*****	29*****	42*****	50*****	59*****	U/L
AST	47	24	23	28	23	31	39	U/L
Urea Nitrogen (BUN)	23	16	16	15	16	14	13	mg/dL
Calcium	10.1	9.9	10.02	10.48	10.37	10.02	9.78	mg/dL
Creatinine	0.7818	0.764	0.7105	0.6511	0.6658	0.7103	0.6814	mg/dL
GGT	75	59	45	53	66	73	68	U/L
Glucose	116	114	302*_*	62	147	56	103	mg/dL
I. Phosphorous	3.4	6.4	5	5.5	6.5	5.3	6.6	mg/dL
Total Bilirubin	0.24	0.22	0.14	0.11	0.14	0.13	0.18	mg/dL
Total Protein	6.814	6.234	6.015	6.64	6.607	6.529	6.44	g/dL
Sodium	144	144	143	143	144	143	145	mmol/L
Potassium	3.9	4.5	4.4	4.3	4	3.3	3.8	mmol/L
Chloride	103	103*	100*	102*	102*	101*	102*	mmol/L
Globulin	2.043	1.808*	2.322*	2.728	2.511*	2.162*	2.157*	
Alb/Glob Ratio	2	2	2	1	1	2	2	

* Elevated/Low Levels, Comparable to baseline
** Elevated/Low Levels, Not of clinical concern
***Elevated/Low Levels likely due to stress response associated with surgery
****Muscle component, indicative of possible damage to muscle - may be related to anesthesia injections or damage to muscle during surgery
*****Elevated ALT levels are considered of clinical concern only if the levels are more than 100 IU/L.
******Elevated Globulin levels than Albumin levels are indicative of inflammation, possibly due to implanted microcapsules, encapsulated cells or cell products
*******Elevated levels, Not of clinical concern; Possibly mild inflammation of liver but transient
. Elevated/ Low Levels likely due to Immunosuppression/implanted biomaterial/encapsulated cells/cell products
_ Elevated due to diabetic condition of the animal

Table S8 (Continued). Blood chemistry panel for NHPs over the course of the TP-IAT to the omentum with PPCN study.

(n = 2; 1 female (F), 1 male (M)); ALT = alanine transaminase; AST = aspartate aminotransferase; BUN = blood urea nitrogen; GGT = gamma-glutamyl transferase; I = inorganic.

**Monkey:**	**RH9144 (M)**					
**Condition:**	Post-Transplant					
**Date:**	42	63	70	77	84	
**Day:**	8/2/2022	8/23/2022	8/30/2022	9/6/2022	9/13/2022	**Units**
Albumin	4.433	4.616	4.403	4.289	4.302	g/dL
Alkaline Phosphatase	419	506	471	501	488	U/L
ALT	36*****	39*****	40*****	38*****	31*****	U/L
AST	26	25	31	23	21	U/L
Urea Nitrogen (BUN)	18	21	20	19	19	mg/dL
Calcium	10.03	10.65	10.36	10.25	10.21	mg/dL
Creatinine	0.7567	0.8618	0.7737	0.7748	0.8474	mg/dL
GGT	77	87	88	91	86	U/L
Glucose	106	150	110	109	229	mg/dL
I. Phosphorous	6	6.1	5.8	5.6	6.6	mg/dL
Total Bilirubin	0.14	0.12	0.17	0.18	0.2	mg/dL
Total Protein	6.656	6.717	6.305	6.45	6.546	g/dL
Sodium	143	151	144	141	147	mmol/L
Potassium	3.7	4	3.8	4.1	4.4	mmol/L
Chloride	101*	108	103*	102*	106*	mmol/L
Globulin	2.223*	2.101*	1.902*	2.161*	2.244*	
Alb/Glob Ratio	2	2	2	2	2	

* Elevated/Low Levels, Comparable to baseline
** Elevated/Low Levels, Not of clinical concern
***Elevated/Low Levels likely due to stress response associated with surgery
****Muscle component, indicative of possible damage to muscle - may be related to anesthesia injections or damage to muscle during surgery
*****Elevated ALT levels are considered of clinical concern only if the levels are more than 100 IU/L.
******Elevated Globulin levels than Albumin levels are indicative of inflammation, possibly due to implanted microcapsules, encapsulated cells or cell products
*******Elevated levels, Not of clinical concern; Possibly mild inflammation of liver but transient
. Elevated/ Low Levels likely due to Immunosuppression/implanted biomaterial/encapsulated cells/cell products
_ Elevated due to diabetic condition of the animal

Table S8 (Continued). Blood chemistry panel for NHPs over the course of the TP-IAT to the omentum with PPCN study.
(n = 2; 1 female (F), 1 male (M)); ALT = alanine transaminase; AST = aspartate aminotransferase; BUN = blood urea nitrogen; GGT = gamma-glutamyl transferase; I = inorganic.

**Monkey:**	**RH9144 (M)**					
**Condition:**	Post-Transplant				Post-Omentectomy	
**Date:**	91	98	105	112	118	
**Day:**	9/20/2022	9/27/2022	10/4/2022	10/11/2022	10/17/2022	**Units**
Albumin	4.242	4.57	4.536	4.527	4.686	g/dL
Alkaline Phosphatase	484	477	487	479	455	U/L
ALT	30*****	28*****	29*****	31*****	59*****	U/L
AST	25	31	23	29	42	U/L
Urea Nitrogen (BUN)	21	21	20	12	28**	mg/dL
Calcium	10.01	10.3	10.32	10.16	10.11	mg/dL
Creatinine	0.8133	0.8157	0.8883	0.8628	1.0647	mg/dL
GGT	82	90	88	84	79	U/L
Glucose	70	126	132	112	237*_*	mg/dL
I. Phosphorous	5.9	6.7	6.3	6.5	8	mg/dL
Total Bilirubin	0.19	0.17	0.18	0.2	0.27	mg/dL
Total Protein	6.307	6.685	6.698	6.5	7.228	g/dL
Sodium	143	155	144	143	144	mmol/L
Potassium	3.5	3.8	3.8	3.7	4.7	mmol/L
Chloride	102*	109	101*	102*	96*	mmol/L
Globulin	2.065*	2.115*	2.162*	2.062*	2.542	
Alb/Glob Ratio	2	2	2	2	2	

* Elevated/Low Levels, Comparable to baseline
** Elevated/Low Levels, Not of clinical concern
***Elevated/Low Levels likely due to stress response associated with surgery
****Muscle component, indicative of possible damage to muscle - may be related to anesthesia injections or damage to muscle during surgery
*****Elevated ALT levels are considered of clinical concern only if the levels are more than 100 IU/L.
******Elevated Globulin levels than Albumin levels are indicative of inflammation, possibly due to implanted microcapsules, encapsulated cells or cell products
*******Elevated levels, Not of clinical concern; Possibly mild inflammation of liver but transient

Table S9. Urinalysis for nonhuman primates (NHPs) over the course of the total pancreatectomy followed by islet autotransplantation (TP-IAT) to the omentum with PPCN study.
(n = 2; 1 female (F), 1 male (M)); RBC = red blood cells; WBC = white blood cells.

	Monkey:	RH9139 (F)						
	Condition:	IVDTT Baseline	Pre-Transplant	Day of Transplant	Post-Transplant			
	Date:	8/9/2022	8/15/2022	8/16/2022	8/19/2022	8/23/2022	8/30/2022	9/6/2022
	Day:	-7	-1	0	3	7	14	21
Physical	Appearance	Clear	Clear	Clear	Clear	Clear	Clear	Clear
	Specific Gravity	1.002	1.009	1.012	1.008	1.028	1.005	1.003
	Color	Colorless	Light Yellow	Light Yellow	Colorless	Yellow	Colorless	Colorless
Dipstick Evaluation	Leukocytes	Negative	Negative	Negative	Negative	Negative	Negative	Negative
	Nitrite	Negative	Negative	Negative	Negative	Negative	Negative	Negative
	pH	7	8	6	7	8	8	7
	Protein	Trace	Negative	Trace	Trace	Trace	Negative	Negative
	Glucose	Normal	Normal	Normal	100mg/dl	Normal	Normal	250 mg/dl
	Ketones	Negative	Negative	Negative	Negative	Negative	Negative	Negative
	Urobilinogen	Normal	Normal	Normal	Normal	Normal	Normal	Normal
	Bilirubin	Negative	Negative	Negative	Negative	Negative	Negative	Negative
	Blood	Negative	Trace	50	Negative	Negative	Negative	Negative

	Monkey:	RH9139 (F)						
	Condition:	Post-Transplant	Glucagon Post-Transplant	Post-Transplant		IVDTT Post-Transplant	Post-Transplant	
	Date:	9/13/2022	9/20/2022	9/27/2022	10/4/2022	10/10/2022	10/18/2022	10/25/2022
	Day:	28	35	42	49	55	63	70
Physical	Appearance	Hazy	Hazy	Clear	Clear	Clear	Clear	Clear
	Specific Gravity	1.003	1.01	1.01	1.003	1.003	1.007	1.004
	Color	Colorless	Light Yellow	Light Yellow	Colorless	Colorless	Colorless	Colorless
Dipstick Evaluation	Leukocytes	+	Negative	Negative	Negative	Negative	Negative	Negative
	Nitrite	Negative	Negative	Negative	Negative	Negative	Negative	Negative
	pH	8	8	8	8	5	7	7
	Protein	Trace	Trace	Trace	Trace	Negative	Negative	Trace
	Glucose	Normal	Normal	Normal	Normal	500 mg/dl	500 mg/dl	100mg/dl
	Ketones	Negative	Negative	Negative	Negative	Negative	Negative	Negative
	Urobilinogen	Normal	Normal	Normal	Normal	Normal	Normal	Normal
	Bilirubin	Negative	Negative	Negative	Negative	Negative	Negative	Negative
	Blood	Negative	250	Negative	Negative	Negative	Negative	Negative

Table S9 (Continued). Urinalysis for NHPs over the course of the TP-IAT to the omentum with PPCN study.

(n = 2; 1 female (F), 1 male (M)); RBC = red blood cells; WBC = white blood cells.

	Monkey:	RH9139 (F)					
	**Condition:**	Post-Transplant				IVDTT Post-Transplant	Survival Omentectomy Post-Transplant
	**Date:**	11/1/2022	11/7/2022	11/15/2022	11/22/2022	11/29/2022	12/2/2022
	**Day:**	77	83	91	98	105	108
Physical	**Appearance**	Clear	Clear	Clear	Clear	Clear	Clear
	**Specific Gravity**	1.005	1.004	1.012	1.003	1.012	1.023
	**Color**	Colorless	Light Yellow	Light Yellow	Colorless	Light Yellow	Yellow
Dipstick Evaluation	**Leukocytes**	Negative	Negative	+	Negative	Negative	Negative
	**Nitrite**	Negative	Negative	Negative	Negative	Negative	Negative
	**pH**	5	7	8	7	8	9
	**Protein**	Trace	Trace	Trace	Trace	Trace	100
	**Glucose**	Normal	Normal	Normal	50 mg/dl	250mg/dl	Normal
	**Ketones**	Negative	Negative	Negative	Negative	Negative	Negative
	**Urobilinogen**	Normal	Normal	Normal	Normal	Normal	Normal
	**Bilirubin**	Negative	Negative	Negative	Negative	Negative	Negative
	**Blood**	Negative	Negative	Trace	Negative	Trace	Negative

	Monkey:	RH9139 (F)	
	**Condition:**	IVDTT Post - Omentectomy	Termination & Necropsy
	**Date:**	12/5/2022	12/8/2022
	**Day:**	111	114
Physical	**Appearance**	Clear	Hazy
	**Specific Gravity**	1.025	1.014
	**Color**	Light Yellow	Light Yellow
Dipstick Evaluation	**Leukocytes**	Negative	Negative
	**Nitrite**	Negative	Negative
	**pH**	5	8
	**Protein**	Negative	Trace
	**Glucose**	1000mg/dl	Normal
	**Ketones**	++ (Moderate)	Negative
	**Urobilinogen**	Normal	Normal
	**Bilirubin**	Negative	Negative
	**Blood**	Negative	Negative

Table S9 (Continued). Urinalysis for NHPs over the course of the TP-IAT to the omentum with PPCN study.

(n = 2; 1 female (F), 1 male (M)); RBC = red blood cells; WBC = white blood cells.

	Monkey:	RH9144 (M)						
	Condition:	Pre-Transplant		Post-Transplant				
	Date:	0	7	14	21	28	42	49
	Day:	6/20/2022	6/28/2022	7/5/2022	7/12/2022	7/19/2022	8/2/2022	8/9/2022
Physical	Appearance	Clear	Clear	Clear	Clear	Clear	Clear	Clear
	Specific Gravity	1.004	1.016	1.02	1.003	1.007	1.008	1.004
	Color	Light Yellow	Colorless	Yellow	Light Yellow	Light Yellow	Yellow	Colorless
Dipstick Evaluation	Leukocytes	Negative	Negative	Trace	Negative	Negative	Negative	Negative
	Nitrite	Negative	Negative	Negative	Negative	Negative	Negative	Negative
	pH	7	7	8	7	8	8	7
	Protein	Trace	Trace	Trace	Trace	Trace	Trace	Trace
	Glucose	Normal	500mg/dl	Normal	50mg/dl	Normal	Normal	Normal
	Ketones	Negative	Negative	Negative	Negative	Negative	Negative	Negative
	Urobilinogen	Normal	Normal	Normal	Normal	Normal	Normal	Normal
	Bilirubin	Negative	Negative	Negative	Negative	Negative	Negative	Negative
	Blood	250Ery/µl	Trace	50Ery/µl	Negative	Negative	Negative	Negative

	Monkey:	RH9144 (M)						
	Condition:	Post-Transplant						
	Date:	63	70	77	84	91	98	105
	Day:	8/23/2022	8/30/2022	9/6/2022	9/13/2022	9/20/2022	9/27/2022	10/4/2022
Physical	Appearance	Clear	Clear	Clear	Clear	Hazy	Clear	Clear
	Specific Gravity	1.023	1.011	1.003	1.006	1.008	1.027	1.004
	Color	Light Yellow	Light Yellow	Colorless	Light Yellow	Light Yellow	Yellow	Light Yellow
Dipstick Evaluation	Leukocytes	Negative	Negative	Negative	Negative	Negative	Negative	Negative
	Nitrite	Negative	Negative	Negative	Negative	Negative	Negative	Negative
	pH	8	8	7	7	7	9	8
	Protein	Trace	Trace	Trace	Trace	Trace	30	Negative
	Glucose	Normal	Normal	Normal	500mg/dl	Normal	Normal	Normal
	Ketones	Negative	Negative	Negative	Negative	Negative	Negative	Negative
	Urobilinogen	Normal	Normal	Normal	Normal	Normal	Normal	Normal
	Bilirubin	Negative	Negative	Negative	Negative	Negative	Negative	Negative
	Blood	Negative	50	Negative	Negative	Negative	Negative	Negative

Table S9 (Continued). Urinalysis for NHPs over the course of the TP-IAT to the omentum with PPCN study.

(n = 2; 1 female (F), 1 male (M)); RBC = red blood cells; WBC = white blood cells.

	Monkey:	RH9144 (M)	
	Condition:	Post-Transplant	Post-Omentectomy
	Date:	112	118
	Day:	10/11/2022	10/17/2022
Physical	Appearance	Clear	Clear
	Specific Gravity	1.02	1.025
	Color	Yellow	Light Yellow
Dipstick Evaluation	Leukocytes	Negative	Negative
	Nitrite	Negative	Negative
	pH	9	5
	Protein	30	Trace
	Glucose	Normal	1000mg/dl
	Ketones	Negative	++
	Urobilinogen	Normal	Normal
	Bilirubin	Negative	Negative
	Blood	Negative	Negative
